# A Tutorial on Measurement of Electrochemical Water-Splitting Performance Parameters: Methods and Best Practices

**DOI:** 10.1021/acsmeasuresciau.6c00147

**Published:** 2026-08-21

**Authors:** Minakshi Sharma, Chandra Prakash, Vijay K. Singh, Ambesh Dixit

**Affiliations:** † Advanced Materials and Device (A-MAD) Laboratory, Department of Physics, 231955 Indian Institute of Technology Jodhpur, Jodhpur, Rajasthan-342030, India; ‡ Rishabh Centre for Research and Innovation in Clean Energy, Indian Institute of Technology Jodhpur, Jodhpur, Rajasthan-342030, India; § Department of Physics, 226963 Indian Institute of Technology Ropar, Rupnagar, Punjab-140001, India

**Keywords:** Water electrolysis, electrocatalysts, HER mechanism, OER mechanism, electrochemical performance, parameter protocols, alkaline water electrolyzer, industrial performance, large-scale electrode, gas-diffusion layer, anion exchange membrane

## Abstract

Electrochemical water splitting has emerged as a promising route to sustainable, green hydrogen production, playing a key role in advancing clean energy systems. The continued advancement of water electrolysis relies heavily on the rational design and evaluation of efficient, durable, and cost-effective electrocatalysts. However, a meaningful and fair comparison of electrocatalyst performance depends on accurate and standardized measurement protocols. This tutorial provides an in-depth overview of the best measurement practices for assessing performance parameters, including overpotential, Tafel slope, charge transfer, electrolyte resistance, electrochemical surface area, turnover frequency, stability, and Faradaic efficiency of electrocatalysts for water splitting. More emphasis is given on highlighting the strengths and limitations of various electrochemical methods, such as linear sweep voltammetry (LSV), cyclic voltammetry (CV), electrochemical impedance spectroscopy (EIS), chronoamperometry (CA), and chronopotentiometry (CP). Further, special attention is given to proper *iR* compensation, selection of appropriate scan rates, and identification of the non-Faradaic region for accurate determination of electrochemical surface area (ECSA). The role of redox peak integration in estimating active site densities, particularly for transition-metal-based electrocatalysts, is also a focus. The transition from standard three-electrode testing to a full-cell alkaline water electrolyzer is also discussed in detail, highlighting standard protocols to minimize the gap between lab-scale testing and commercial-scale electrolyzers. Thus, overall, this tutorial can be considered a practical guide for academicians and researchers, especially beginners in the field, aiming to enhance the reliability of electrochemical measurements and further catalyze the innovation and development of high-performance, energy-efficient water-splitting electrocatalysts for green hydrogen production.

## Graphical abstract

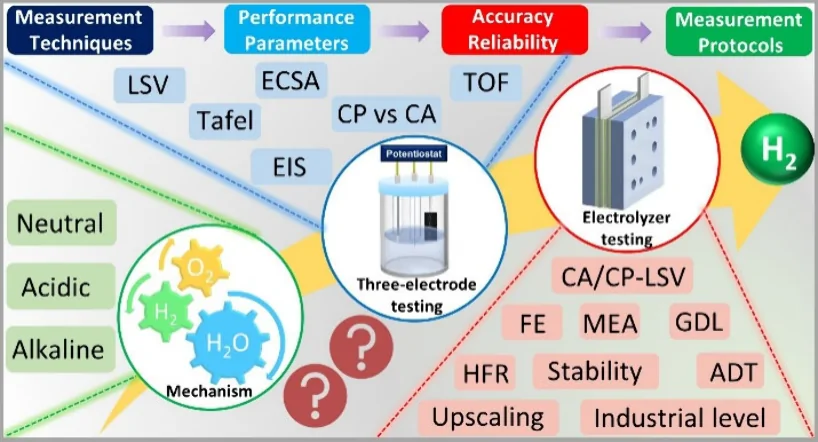

## 1 Introduction

The increasing global demand for clean energy, coupled with the urgent need to reduce greenhouse gas emissions, has brought green hydrogen (G-H_2_) to the forefront as a key enabler of a sustainable energy source for the future. Unlike gray hydrogen, which is derived from fossil fuels and emits a significant amount of CO_2_, G-H_2_ is generated by splitting water molecules using electricity produced from renewable sources known as electrochemical water splitting (EWS).^1,2^ Thus, EWS is a process that breaks water (H_2_O) into hydrogen (H_2_) and oxygen (O_2_) molecules by providing energy equivalent to, or greater than, the thermodynamic water-splitting potential, applied through electrodes immersed in water. In this process, H_2_ and O_2_ molecules are generated at the cathode and anode, respectively. The integrated device that facilitates these electrochemical reactions is commonly known as a water electrolyzer.^3^ This process emits no harmful byproducts, making it a clean and sustainable alternative. The need for G-H_2_ is in demand due to several factors, such as limited reserves of conventional fossil-based energy sources, the intermittent nature of renewable energy sources, and increasing commitment of governments and industries to achieve net-zero carbon targets.^4^ It offers a carbon-free solution for a variety of applications, including synthetic fuel, an energy source for H_2_-powered vehicles, power generation, a primary source for ammonia production as a fertilizer, etc.,^5^ as presented in Figure 1a.

**Figure 1:**
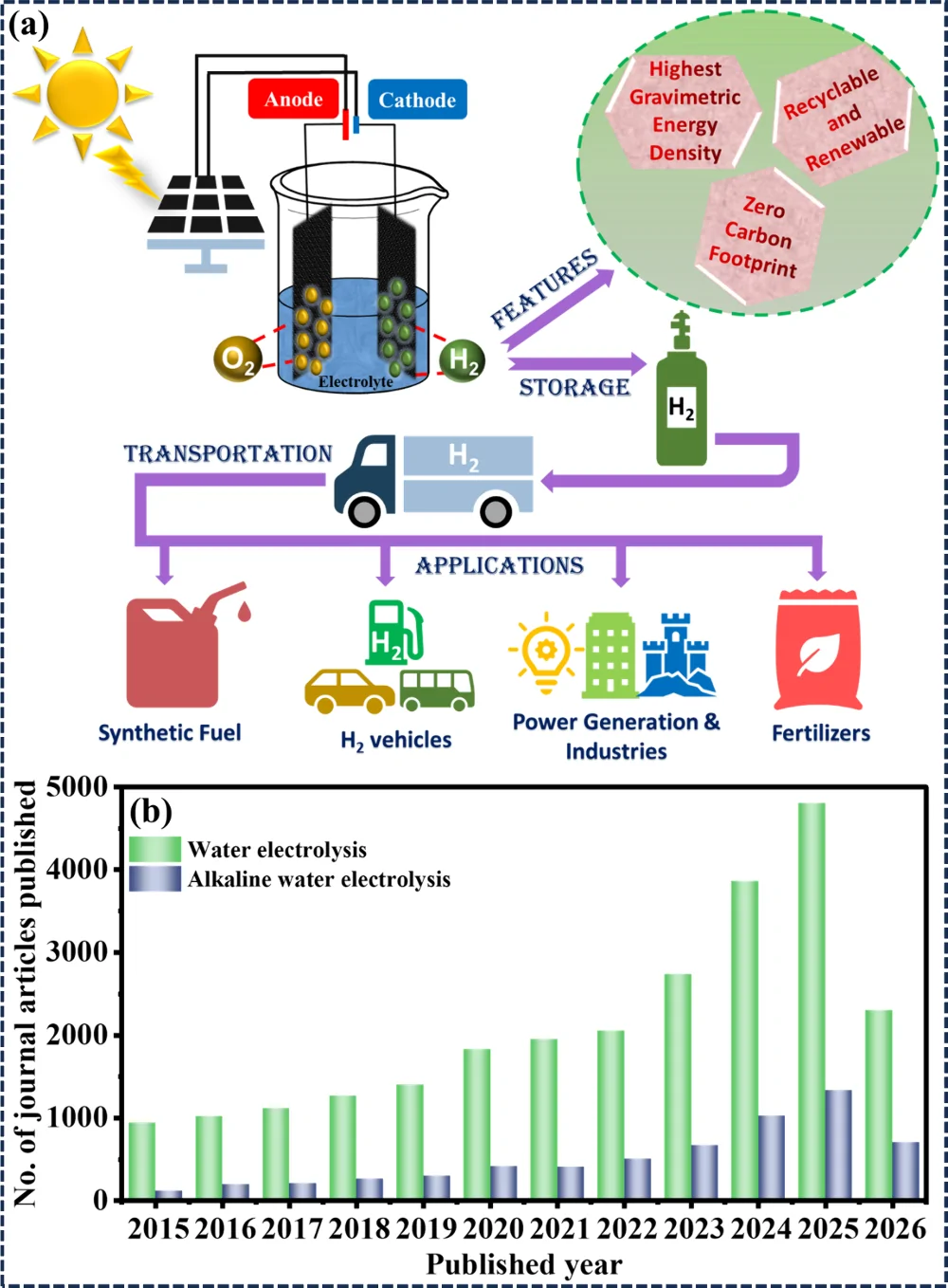
(a) Schematic illustration showing the key enabling process for the green hydrogen economy, including hydrogen production, storage, transportation, and utilization for various applications. (b) Number of journal articles from 2015 to 2026 related to hydrogen production (source: www.lens.org).

Although the thermodynamic water-splitting voltage is 1.23 V vs SHE, practical electrolysis requires a higher cell voltage due to kinetic limitations associated with reactions, as well as ohmic losses and mass transport limitations within the electrolyzer.^6^ Therefore, significant research efforts have been directed to developing highly active, earth-abundant, and durable electrocatalysts that can reduce reaction overpotentials, accelerate charge-transfer kinetics, and sustain long-term operational stability.^7^ Driven by these scientific and technological challenges, research in EWS has expanded rapidly over the past decade. This growing interest is reflected by the substantial increase in scientific publications related to water electrolysis and alkaline water electrolysis between 2015 and 2026 (source: www.lens.org) Figure 1b.

The rapid expansion of electrocatalyst research, however, has introduced a significant challenge: the reliable evaluation and comparison of catalytic performance. The discrepancies in electrochemical measurements have become one of the major obstacles preventing meaningful benchmarking and translation of laboratory-scale discoveries into practical water electrolyzer technologies. Thus, to assess the performance of an electrocatalyst, a comprehensive understanding of electrochemical measurement techniques and their associated experimental parameters is required. The performance and efficiency of water electrolyzers are governed by multiple interrelated factors that influence the electrochemical processes occurring at the electrode–electrolyte interface. These processes can be quantitatively evaluated by monitoring three fundamental electrochemical parameters: potential, current, and charge, using a standard electrochemical workstation. Variations in these electrochemical signals arise from interfacial charge-transfer reactions, adsorption/desorption of reaction intermediates, mass transport, and the physicochemical properties of the electrode and electrolyte.^8^ By correlating these electrochemical responses with catalyst properties, researchers can assess electrocatalyst performance, identify kinetic limitations, and optimize electrolyzer operation.

Among the available electrochemical techniques, linear sweep voltammetry (LSV) is the most widely used method for assessing electrocatalytic activity, providing polarization curves that relate current density to the applied potential and enable the determination of reaction overpotentials.^9^ However, the interpretation of LSV data is often influenced by several experimental factors, including scan rate, *iR* compensation, electrolyte composition and its pH, since each of these factors has a unique relevance.^10,11^ Similarly, cyclic voltammetry (CV) offers valuable information regarding redox processes, reaction mechanisms, and surface electrochemical properties of electrocatalysts.^12^ Long-term operational stability, which is essential for practical deployment, is generally assessed using chronoamperometry (CA) or chronopotentiometry (CP), enabling the evaluation of catalyst durability under sustained operating conditions.^13^

Beyond activity and stability assessments, understanding reaction kinetics is essential for elucidating electrocatalytic mechanisms. Parameters such as the Tafel slope provide critical insights into reaction pathways, rate-determining steps, and charge-transfer kinetics. Tafel analysis can be derived using multiple electrochemical techniques, including CV, CP, CA, and LSV measurements, although the selected methodology can influence the resulting kinetic parameters.^14–16^ Therefore, clear guidelines for Tafel slope determination are necessary to ensure reliable interpretation and comparison of catalytic performance. Electrochemical impedance spectroscopy (EIS) is another powerful technique for probing interfacial phenomena, charge-transfer resistance, mass transport characteristics, and electrode kinetics. Nevertheless, significant inconsistencies often arise due to variations in measurement protocols, including differences in applied potential, frequency range, perturbation amplitude, and electrode configuration.^17,18^ Consequently, researchers frequently encounter difficulties when comparing EIS data reported across different studies. Systematic investigations of frequency-, amplitude-, and potential-dependent EIS measurements are therefore presented to establish standardized practices and minimize ambiguity in impedance analysis.

Another important parameter for evaluating electrocatalyst performance is the electrochemically active surface area (ECSA), which represents the number of electrochemically accessible active sites available for catalytic reactions. It is commonly derived from the electrochemical double-layer capacitance (*C*_dl_), obtained by measuring capacitive currents within a non-Faradaic potential region at various scan rates.^19,20^ However, ECSA estimation is highly sensitive to the selection of the non-Faradaic potential window, as variations in the chosen potential range can significantly alter the measured *C*_dl_ and consequently the calculated ECSA, even for the same catalyst.^21^ Although low overpotential and high current density are widely regarded as key indicators of electrocatalytic performance, they do not necessarily reflect a catalyst’s intrinsic activity, as these metrics are strongly influenced by the number of accessible active sites. Therefore, intrinsic catalytic activity is commonly normalized with respect to ECSA, making the accurate determination of ECSA essential for meaningful comparisons among different electrocatalysts. Turnover frequency (TOF) is widely recognized as the most reliable descriptor of the intrinsic activity of an electrocatalyst because it quantifies the number of product molecules generated per active site per unit time, independent of catalyst loading or electrode geometry. Although accurately identifying the true number of active sites remains a major challenge, particularly for multimetallic and metal-free catalysts, careful determination of active-site density, real electrochemical surface area, and faradaic efficiency (FE) substantially improves the reliability of TOF values. Consequently, TOF should be reported alongside conventional activity metrics using transparent calculation procedures and experimentally measured FE, thereby enabling meaningful comparison of intrinsic catalytic activity across different electrocatalysts and studies.

Furthermore, while three-electrode measurements remain the standard approach for evaluating intrinsic electrocatalytic activity under laboratory conditions, their results often do not translate directly to practical electrolyzer operation because of substantial differences in the electrochemical environment, electrode architecture, catalyst loading, mass transport, gas evolution, and system integration.^22,23^ In practical devices, additional factors such as the properties of the gas diffusion layer, including its porosity, wettability, electrical conductivity, and gas–liquid transport characteristics, significantly influence catalyst utilization, reactant accessibility, bubble removal, and overall cell performance. Moreover, scaling electrocatalysts from laboratory-scale electrodes with low catalyst loadings and small geometric areas to industrially relevant large-area electrodes introduces challenges associated with uniform catalyst deposition, mechanical adhesion, electronic conductivity, electrolyte distribution, and current density homogeneity. Consequently, standardized testing protocols that bridge intrinsic catalyst evaluation under three-electrode conditions with realistic full-cell electrolyzer performance are increasingly recognized as essential for establishing reliable structure–performance relationships and accelerating the commercialization of efficient water electrolysis technologies.

In light of these considerations, this tutorial aims to provide a comprehensive framework for measuring and interpreting electrochemical water-splitting performance parameters. Initially, the electrocatalytic reaction mechanisms in different electrolyte environments are discussed. Subsequently, the tutorial summarizes the number of experiments on carbon cloth (CC) and nickel foam (NF) in an alkaline electrolyte. Key characterization techniques, including LSV, CV, CA/CP, ECSA determination, TOF analysis, Tafel kinetics, and EIS measurements, are discussed in detail. Particular emphasis is placed on the experimental protocols, data interpretation strategies, and common sources of error associated with each technique. Finally, the tutorial extends beyond conventional three-electrode studies by discussing full-cell alkaline water electrolyzer testing protocols, thereby providing researchers with a unified framework for translating intrinsic catalyst performance into realistic device-level evaluation. By providing standardized measurement protocols and best-practice recommendations, this tutorial seeks to assist researchers in accurately evaluating electrocatalyst performance and advancing the development of efficient and reliable water-splitting technologies for sustainable H_2_ generation.

## 2 Mechanism of Water Splitting

Water electrolysis is fundamentally an electrochemical redox process driven by an applied electric potential. During electrolysis, the oxygen evolution reaction (OER) occurs at the anode, where water or hydroxide ions are oxidized to molecular oxygen (O_2_), accompanied by the transfer of four electrons.^24^ It is the main kinetic bottleneck of overall water splitting because it involves a complex four-electron-coupled electron transfer mechanism and the energetically demanding formation of the O–O bond following O–H bond cleavage at catalytically active sites.^25^ Consequently, OER requires a substantial overpotential to achieve practical reaction rates. The electrolyte pH has a significant impact on the reaction mechanism, as the identities of the reactant species and the sequence of elementary reaction steps differ across electrolyte media. In acidic electrolytes, water molecules serve as the reactant, and their oxidation generates oxygen, protons (H^+^), and electrons. On the contrary, in alkaline (and near-neutral) electrolytes, hydroxide ions (OH^–^) participate directly in the anodic oxidation process, producing oxygen, water, and electrons.^26^ These distinct reaction pathways lead to differences in intermediate adsorption, reaction kinetics, and catalyst design considerations. The overall OER reactions in acidic and alkaline media are presented in the following sections.

### 2.1 OER in Acidic Electrolyte

The overall OER reaction in an acidic medium is given by eqs 1–5:

$$H_{2}O+M\to \mathrm{OH-M}+H^{+}+e^{-}$$ 1

$$\mathrm{OH-M}\to \mathrm{M-O}+H^{+}+e^{-}$$ 2

$$\mathrm{M-O}+H_{2}O\to \mathrm{OOH-M}+H^{+}+e^{-}$$ 3

$$\mathrm{M-OOH}\to O_{2}+M+H^{+}+e^{-}$$ 4

$$2\mathrm{M-O}\to O_{2}+2M$$ 5

### 2.2 OER in Alkaline Electrolyte

The overall OER reaction in an alkaline medium is given by eqs 6–10:

6 $${\mathrm{OH}}^{-}+M\to \mathrm{M-OH}+e^{-}$$

7 $$\mathrm{M-OH}+{\mathrm{OH}}^{-}\to H_{2}O+\mathrm{M-O}+e^{-}$$

8 $$\mathrm{M-O}+{\mathrm{OH}}^{-}\to \mathrm{M-OOH}+e^{-}$$

9 $$\mathrm{M-OOH}+{\mathrm{OH}}^{-}\to H_{2}O+O_{2}+e^{-}$$

10 $$2\mathrm{M-O}\to O_{2}+M$$

In these reaction schemes, M represents the catalytically active site of the electrocatalyst. Initially, a water molecule (or hydroxide ion in alkaline media) adsorbs onto the active site to form the hydroxyl intermediate (M–OH). Subsequent deprotonation and electron transfer produce the adsorbed oxygen intermediate (M–O). The M–O species then reacts with another water molecule (or hydroxide ion, depending on the electrolyte) to generate the hydroperoxyl intermediate (M–OOH), which undergoes a final electron transfer step to form adsorbed molecular oxygen (O_2_). The sluggish kinetics of the OER primarily originate from the thermodynamically and kinetically demanding formation and transformation of the surface-bound intermediates, particularly the O–O bond formation step, which contributes significantly to the overall overpotential required for oxygen evolution.^27,28^

### 2.3 OER in Neutral Electrolyte

In neutral electrolytes, the OER proceeds through a multistep proton-coupled electron transfer (PCET) mechanism involving four electron-transfer steps and the sequential formation of oxygen-containing surface intermediates. Initially, water molecules adsorb onto the catalytically active sites (M) of the electrocatalyst, followed by successive deprotonation steps to form surface-bound hydroxyl (M–OH) and oxo (M–O) intermediates. Subsequently, the adsorbed M–O species undergoes nucleophilic attack by a water molecule (or hydroxide species generated locally), leading to the formation of the hydroperoxyl intermediate (M–OOH) and the crucial O–O bond. In the final step, M–OOH undergoes further electron transfer, releasing molecular oxygen (O_2_) and regenerating the active site. Alternatively, on certain catalyst surfaces, oxygen evolution may proceed through the coupling of two adjacent M–O intermediates via the lattice-oxygen or adsorbate-evolution pathway, depending on the catalyst structure and reaction conditions. These elementary steps collectively determine the reaction kinetics and overpotential of the OER in neutral media,^29,30^ as presented in eqs 11–15:

$$H_{2}O+M\to \mathrm{OH-M}+H^{+}+e^{-}$$ 11

$$\mathrm{OH-M}\to \mathrm{M-O}+H^{+}+e^{-}$$ 12

$$\mathrm{O-M}+H_{2}O\to \mathrm{OOH-M}+H^{+}+e^{-}$$ 13

$$\mathrm{M-OOH}\to O_{2}+M+H^{+}+e^{-}$$ 14

$$2\mathrm{M-O}\to O_{2}+2M$$ 15

### 2.4 HER in Neutral Electrolyte

In neutral electrolytes, since the concentration of free protons is low, water molecules act as the primary proton source.^31,32^ In the reaction schemes, M denotes the catalytically active site of the electrocatalyst, while M–H represents the adsorbed hydrogen intermediate. The HER is initiated by the Volmer step, in which a water molecule is electrochemically dissociated to form an adsorbed hydrogen atom (M–H) on the catalyst surface, accompanied by the generation of a hydroxide ion. Subsequently, H_2_ evolution proceeds either via the Heyrovsky step, where M–H reacts with another water molecule and an electron to produce molecular H_2_, or through the Tafel step, in which two adjacent M–H intermediates recombine to form H_2_. The relative contributions of the Volmer–Heyrovsky and Volmer–Tafel pathways depend on electrocatalyst properties, hydrogen adsorption energy, and operating conditions.^33,34^ The HER reaction steps are given below in eqs 16–18:

Volmer:

16 $$H_{2}O+e^{-}+M\to \mathrm{M-H}+{\mathrm{OH}}^{-}$$

Heyrovsky:

17 $$H_{2}O+e^{-}+\mathrm{M-H}\to H_{2}+{\mathrm{OH}}^{-}+M$$

Tafel:

18 $$2\mathrm{M-H}\to H_{2}+2M$$

### 2.5 HER in Acidic Electrolyte

HER involves a two-step procedure to generate H_2_ in an acidic electrolyte. The first phase is the protonation process, referred to as a Volmer step reaction, in which a proton accepts an electron and forms the adsorbed hydrogen intermediate state (M–H or H_ad_) on the electrocatalyst surface. The following step is the Heyrovsky reaction, in which a H_2_ molecule is produced when a hydrogen intermediate state reacts with a proton and an electron. On the other hand, the Tafel recombination process occurs when two adsorbed hydrogen atoms react.^35,36^ The HER reaction steps in an acidic medium are given below in eqs 19–21:

19 $$H_{3}O^{+}+e^{-}+M\to \mathrm{M-H}+H_{2}O,\left(120\;\mathrm{mV/dec}\right)$$

20 $$H_{3}O^{+}+e^{-}+\mathrm{M-H}\to H_{2}+H_{2}O+M,\left(40\;\mathrm{mV/dec}\right)$$

21 $$\mathrm{M-H}+\mathrm{M-H}\to H_{2}+2M,\left(30\;\mathrm{mV/dec}\right)$$

### 2.6 HER in Alkaline Electrolyte

In alkaline electrolytes, HER proceeds through either the Volmer–Heyrovsky or Volmer–Tafel mechanism and follows a reaction sequence analogous to that in neutral media, although the initial proton source differs from that in acidic electrolytes. Because free protons are scarce under alkaline conditions, water molecules serve as the H_2_ source. During the Volmer step, a water molecule is electrochemically dissociated at the catalytically active site (M), producing an adsorbed hydrogen intermediate (M–H) and a hydroxide ion (OH^–^), accompanied by electron transfer. The adsorbed hydrogen is subsequently converted into molecular hydrogen through either the Heyrovsky step, in which M–H reacts with another water molecule and an electron to release H_2_ while regenerating OH^–^, or the Tafel step, where two adjacent adsorbed hydrogen intermediates recombine to form H_2_ and regenerate the catalyst surface. The HER reaction steps in alkaline medium are given below in eqs 22–24:

Volmer:

22 $$H_{2}O+e^{-}+M\to \mathrm{M-H}+{\mathrm{OH}}^{-}$$

Heyrovsky:

23 $$H_{2}O+e^{-}+\mathrm{M-H}\to H_{2}+{\mathrm{OH}}^{-}+M$$

Tafel:

24 $$2\mathrm{M-H}\to H_{2}+2M$$

Although the elementary reaction pathways in acidic, neutral, and alkaline electrolytes are conceptually similar, the slower water dissociation kinetics in alkaline media generally makes the Volmer step the rate-determining step, resulting in slower HER kinetics compared with acidic conditions.^37^ A schematic overview of the EWS mechanisms in different electrolyte media (alkaline, neutral, and acidic) is presented in Figure 2.

**Figure 2:**
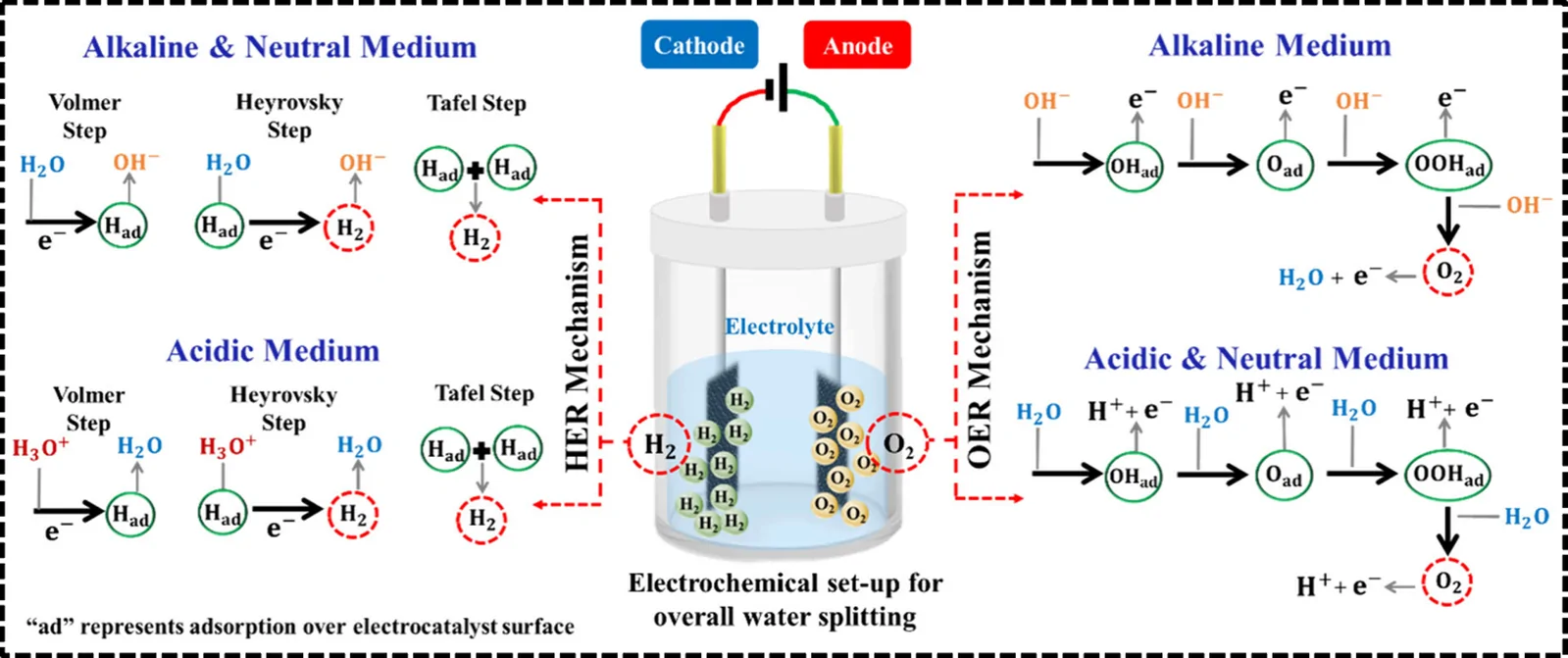
Mechanism of electrochemical water splitting in alkaline, neutral, and acidic electrolyte media.

## 3 Performance Parameters and Techniques for their Evaluation

### 3.1 Linear Sweep Voltammetry (LSV)

LSV is widely used to evaluate the electrocatalytic activity of HER and OER electrocatalysts. In LSV, the electrode potential is swept linearly with time from a starting potential to a predetermined final potential at a constant scan rate (measured in mV/s or V/s) and the resulting current response is continuously recorded. In LSV, the applied potential is swept from a region where negligible electrochemical reaction occurs to more negative potentials for reduction processes (e.g., HER) or more positive potentials for oxidation processes (e.g., OER). The measured current arises from electrochemical reactions occurring at the electrode surface, enabling the assessment of the kinetics and efficiency of water-splitting catalysts. For water electrolysis, LSV is generally performed using a three-electrode configuration, consisting of a working electrode coated with the electrocatalyst, a reference electrode (e.g., Ag/AgCl, Hg/HgO, Hg/Hg_2_SO_4_ or Hg/Hg_2_Cl_2_) depending upon pH of the electrolyte, and an inert counter electrode, typically a graphite rod, platinum mesh or wire.^38^ To facilitate comparison across studies, all measured potentials are converted to the reversible hydrogen electrode (RHE) scale using the Nernst equation, since the thermodynamic potentials of HER and OER are pH-dependent. The *iR*-compensated HER overpotential (η) is obtained using eqs 25 and 26:

25 $$E_{\mathrm{RHE}}=E_{\mathrm{Hg/HgO}}+\left(0.059\right)\times \mathrm{pH}+0.0983-iR$$

26 $$\eta =E_{\mathrm{RHE}}-0.0\;V$$

where *E*_RHE_ is the potential with respect to the reversible hydrogen electrode, *E*_Hg/HgO_ is the potential with respect to the reference electrode, *iR* is the ohmic drop compensation, 0.0983 is the standard thermodynamic potential of the Hg/HgO­(sat. 1 M NaOH) reference electrode, and the factor 0.059 accounts for the simplified Nernst equation at room temperature (298 K).

The OER overpotential is obtained using eq 27:

27 $$\eta =E_{\mathrm{RHE}}-1.23\;V$$

The LSV spectra provide several important performance parameters for evaluating electrocatalysts. In EWS, it is used to evaluate the catalyst’s properties for both OER and HER, including onset potentials and overpotentials. The onset potential indicates the potential at which measurable catalytic activity begins, while the overpotential represents the additional potential beyond the thermodynamic equilibrium potential required to achieve a specified current density.^39,40^ Overpotential is commonly reported at 10 mA cm^–2^, corresponding approximately to the current density required for a solar-to-hydrogen conversion efficiency of about 10% under ideal conditions.^41^ However, for practical electrolyzers, higher current densities, such as 100, 500, or 1000 mA cm^–2^, are increasingly reported because they better reflect industrial operating conditions. Lower overpotentials at a given current density indicate superior catalytic activity. However, the nature of LSV depends on several factors, such as (i) the working electrode material, (ii) the scan direction and rate, (iii) the electrolyte concentration and pH, (iv) *iR* compensation, and (v) the mass loading of the catalyst. To understand the impact of these factors, we present several experimental examples in the following sections.

#### 3.1.1 Working Electrode Material and Scan Rate

The most commonly used substrates in EWS are NF and CC. The nature of working electrode significantly alters the overpotential and current density, as shown for CC and NF in Figure 3a and 3c, respectively. The CC shows very low current densities even at very high potentials, whereas NF shows high current densities even at significantly low potentials in comparison to CC. Also, there is a redox peak present in LSV of NF due to the oxidation of Ni^2+^ to Ni^3+^, while no such peak is observed in CC. Thus, the working electrode material can alter the LSV spectrum shape and activity. The scan rate at which the LSV is recorded is also very crucial. Scan rate refers to how quickly the potential changes or sweeps over time. We recorded the LSV at scan rates of 1, 2, 5, 10, and 25 mV/s to observe the change in LSV spectra of CC and NF as presented in Figure 3a,c, respectively. The overpotential gets decreased, and current density increases with scan rate for both CC and NF. More specifically, the overpotential at 10 mA cm^–2^ (η_10_) changes from 796 mV to 783, 754, 715, and 647 mV when the scan rate changes from 1 mV/s to 2, 5, 10, and 25 mV/s, respectively, for CC, as presented in Figure 3b. The relative error in η_10_ is calculated using eq 28:

28 $$\mathrm{relative}\;\mathrm{error}\;\mathrm{in}\;\eta_{10}=\frac{\eta_{10}\left(1\right)-\eta_{10}\left(x\right)}{\eta_{10}\left(1\right)}\times 100\%$$

where η_10_(1) and η_10_(*x*) are the overpotentials at 1 mV/s and at different scan rates *x* = 2, 5, 10, or 25 mV/s, respectively. It is observed that at *x* = 2, 5, 10, and 25 mV/s, the errors in η_10_ are 1.63, 5.27, 10.17, and 18.71%, respectively, as shown in Figure 3b.

**Figure 3:**
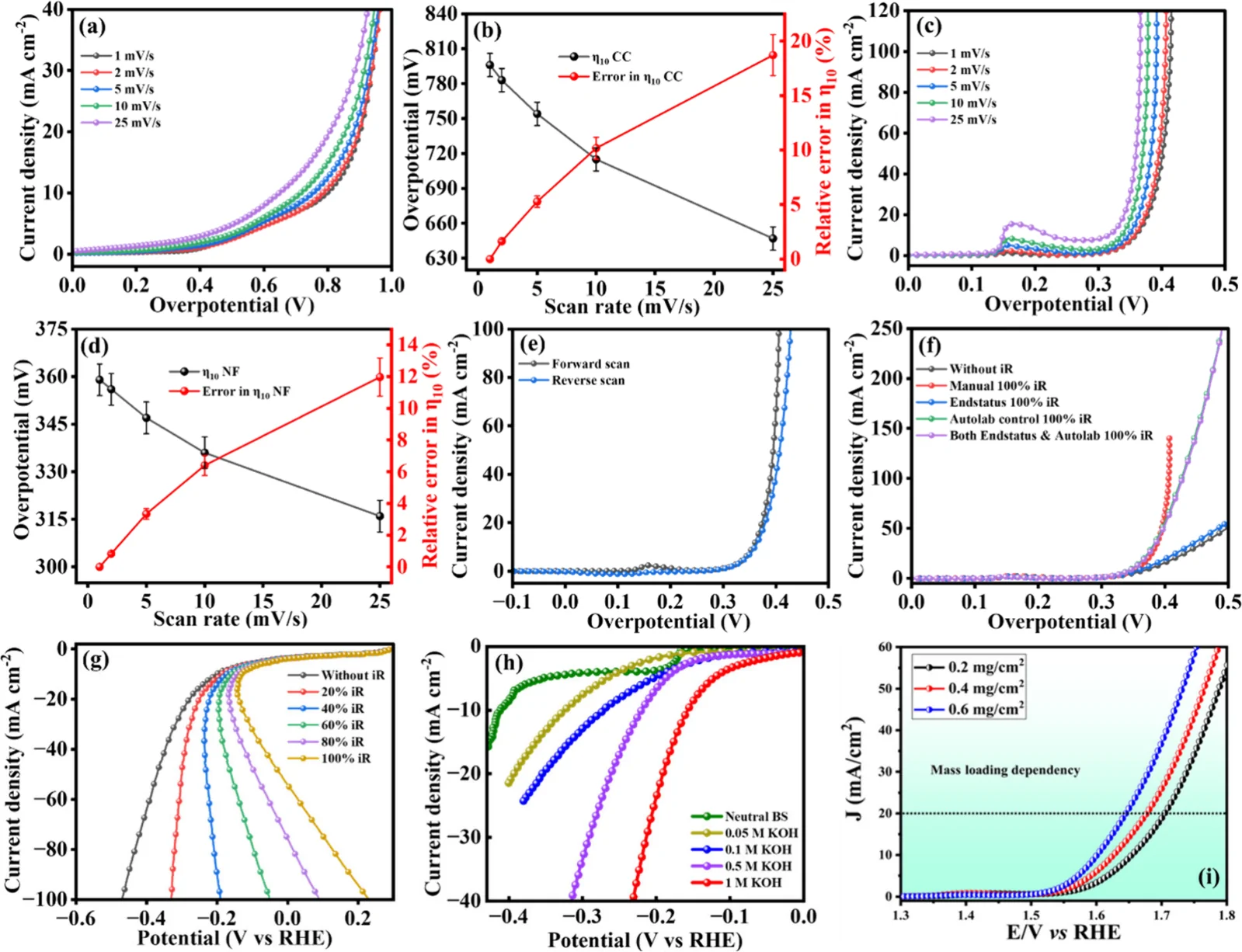
(a) LSV spectra of CC with scan rate variation. (b) Impact of scan rate on overpotential and error in overpotential estimation of CC. (c) LSV spectra of NF with scan rate variation. (d) Impact of scan rate on overpotential and error in overpotential estimation of NF. (e) Impact of direction of scan on the LSV curve of NF. (f) Impact of manual and system-assisted *iR* compensation on the LSV spectrum of NF. (g) Impact of percent of *iR* compensation on the LSV curve of NiS@NF having large *R* of 7 Ω. (h) Impact of electrolyte concentration and pH on the LSV curve of ZNR@NF. Reproduced from ref 2. Copyright 2024 American Chemical Society. (i) Impact of catalyst mass loading on the LSV curve. Reproduced with permission from ref 42. Copyright 2023 the authors of ref 42, exclusive license to Royal Society of Chemistry.

A similar trend of decreasing overpotential with increasing scan rate is also observed for the NF substrate, as shown in Figure 3c. The η_10_ decreases from 359 mV to 356, 347, 336, and 316 mV as the scan rate increases from 1 mV/s to 2, 5, 10, and 25 mV/s, respectively, as shown in Figure 3d. The relative errors in η_10_ at *x* = 2, 5, 10, and 25 mV/s are 0.835, 3.342, 6.406, and 11.97%, respectively. The low scan rates of 1 and 2 mV/s represent true catalytic activity and quasi-steady state, as it has more time to equilibrate at each potential. It shows that lesser capacitive currents arise from double-layer charging, and the contribution to current from the electrocatalyst’s self-oxidation is also minimized.^10^ The higher scan rates of 5, 10, and 25 mV/s can overestimate current densities, making it feel like better false electrocatalytic activity due to increased capacitive current contributions from double-layer charging. The enhanced migration of charged species at a higher potential scan rate would lead to a greater current density, resulting in a reduced overpotential value at a specific current density.^42^ Therefore, it is recommended that the LSV spectra never be recorded at scan rates >2 mV/s for reporting error-free and underestimated overpotentials.

#### 3.1.2 Direction of Scan

Further, in general, the LSV spectrum is recorded in a forward (from low potential to high potential) scan. There are some catalysts, particularly transition-metal-based catalysts, which involve an oxidation peak in OER, as observed for the NF substrate, Figure 3c. The oxidation peak can also contribute to current density enhancement; therefore, it is recommended to use a reverse (from high potential to low potential) scan for these catalysts to get true catalytic activity free from oxidation-controlled activity. To highlight this effect, we have recorded LSV spectra of NF in both forward and reverse directions as presented in Figure 3e. It can be observed that the reverse scan LSV spectrum exhibits lower current densities at a particular applied potential compared to the forward scan.

#### 3.1.3 *iR* Compensation

Another factor that alters the LSV spectrum is the *iR* compensation and its percentage. Here, *i* stands for the current density obtained from the LSV spectrum across the applied potential, and *R* corresponds to the electrolyte or solution or uncompensated resistance. How to evaluate this *R* is covered in the upcoming sections. The majority of the scientific community follows manual 100% *iR* compensation.^43–46^ However, in recent years, several articles have been published using *iR* compensation through the electrochemical potentiostat system itself.^47^ We have collected all data using a Metrohm electrochemical workstation (AUTOLAB, AUT86243, Netherlands). In this electrochemical workstation, there are two options for *iR* compensation, i.e., Endstatus and Autolab control, as shown in Figure S1a,b. We have recorded LSV curves by enabling *iR* compensation through Endstatus only, Autolab control only, and by enabling both Endstatus and Autolab control for NF. The obtained results are presented in Figure 3f. It is observed that none of these *iR* compensations through the system itself match the manual *iR* compensation. The LSV spectra obtained through Autolab control and both Endstatus and Autolab control *iR* compensation show similar current densities at similar potentials; these are much higher compared to manually *iR*-corrected, signifying better false electrochemical activity of NF. In contrast, the LSV curves obtained through Endstatus *iR* compensation show almost a similar trend as observed without *iR*-corrected LSV, signifying either no or very little *iR* compensation. These results impart caution while performing *iR* compensation through the system itself. It might be possible that these errors will change from one system to another. Therefore, it is recommended to compare the results of manual *iR* compensation with system *iR* compensation before reporting surprisingly high current densities at very low overpotentials. The commercialized real systems always operate with existing *R*,^48^ so we recommend reporting bifunctional catalysts with overall cell potential without *iR* compensation and catalysts that are either HER or OER active with both *iR* compensation and without *iR* compensation data. Also, the percentage of *iR* compensation becomes a concern when 100% *iR* compensation gives an unacceptable LSV shape trend, as shown in Figure 3g. We prepared nickel sulfide over nickel substrate (NiS@NF) via sulfurization of NF in the chemical vapor deposition (CVD) system (the synthesis protocols are provided in the Supporting Information; FESEM micrographs are shown in Figure S2a–c, and the EDX spectrum is shown in Figure S2d), and it shows a very high *R* of 7 Ω. The one without *iR*-corrected LSV seems to have an acceptable LSV shape, and upon 100% *iR* compensation, the LSV curve bends toward positive potentials at higher current densities for HER, which is unacceptable. To overcome this issue, we reduced the *iR* compensation percentage and observed that up to 20% *iR* compensation gives an acceptable shape only for the LSV curve. Thus, one can decide one’s own percentage of *iR* compensation based on the value of *R*. However, we still recommend avoiding catalysts with very high *R* values.

#### 3.1.4 Electrolyte Concentration and pH

Another factor that affects the LSV curve is pH and concentration of the electrolyte, which we have highlighted in our previous work.^2^ We have synthesized ZnO nanorods on NF (ZNR@NF) and evaluated their electrochemical performance in neutral buffer solution (BS), 0.05, 0.1, 0.5, and 1 M KOH, as presented in Figure 3h. It is observed that HER activity is sluggish in neutral medium due to two-step reduction processes. In the initial reduction phase, H_3_O^+^ ions serve as the main reactants, occurring at low overpotentials, followed by the reduction of H_2_O molecules at higher overpotentials. On the contrary, in strongly alkaline electrolytes, the reduction of H_2_O molecules occurs in a single step, hence responsible for lower overpotentials. The increase in the electrolyte pH from 7 to 14 altered the localized electric field strength near the electrode–electrolyte interface. This alteration resulted in a higher concentration of cations at the interface, resulting in enhanced HER catalytic activity. The results are in line with the reported literature.^49,50^

#### 3.1.5 Mass Loading of the Catalyst

Karmakar et al.,^42^ highlighted that the catalyst loading can itself vary the LSV spectra and hence overpotentials, as shown in Figure 3i. The commercial nickel oxide (NiO) powder was loaded on CC with different mass loadings. The overpotential decreases as the mass loading increases from 0.2 to 0.6 mg cm^–2^, providing higher OER activity. Hence, it is important to optimize catalyst loading over the current collectors/support substrates to get enhanced activity. The very low catalyst loading will result in higher overpotentials, as the active sites will be lower. On the other hand, very high catalyst loading results in declining catalytic activity as the active sites present on the surface only participate in the reaction, and the rest act as a dead mass.^51,52^ Thus, the catalyst loading should be optimal for maximizing the performance of water electrolyzers in terms of activity, stability, efficiency, and cost.

### 3.2 Electrochemical Impedance Spectroscopy (EIS)

EIS works by applying a sinusoidal signal (AC voltage or AC current) across a broad frequency range to perturb an electrochemical system that is in equilibrium or a stable state. EIS is superior to other electrochemical approaches because it can distinguish between different electrical, electrochemical, and physical processes that occur in an actual electrochemical system and so provide a wealth of information.^53^ It breaks down a complicated electrochemical system into discrete processes with distinct time constants so that it can be readily examined under an applied frequency range.^18,54^ Low frequencies are used to probe slow processes, whereas high frequencies are used to probe fast processes. An analogous electrical circuit made up of typical passive components like resistors, capacitors, and inductors, as well as more complex (distributed) elements coupled in various ways, can be used to fit EIS measurements in an electrochemical system. The plot of real and imaginary impedance is known as the Nyquist plot. It is used to calculate the electrolyte resistance (*R*, used in *iR* compensation) and charge transfer resistance (*R*_ct_) to compare activity trends of different catalysts. This plot greatly depends upon the following parameters: (i) the applied potential, (ii) the frequency range, and (iii) the AC wave amplitude. To test the effect of these parameters, we have accounted for them individually.

#### 3.2.1 Impact of the Applied Potential

The most confusing question for any HER/OER researcher is the following: at what potential value should EIS be conducted? Anantharaj and Noda^17^ highlighted this ambiguity by taking several examples of EIS at the open-circuit potential (OCP), at a constant potential, at different potentials where all catalysts possess a 25 mA cm^–2^ current density, and at a constant potential in catalytic turnover regions. The study revealed that the EIS measurements taken at catalytic turnover conditions only match well with the OER activity of different samples. Inspired by these results, we also performed EIS measurements of pristine CC with varying potential from 1.82 to 2.12 V at a fixed amplitude of 5 mV in the frequency region 10^5^ to 1 Hz, as shown in Figure 4a. The circuit used for fitting these EIS plots and the value of each component are provided in Table S1. The charge transfer resistance decreases as the applied potential increases (Figure 4b). Similarly, for NF, the EIS measurement was performed at various potentials from 1.50 to 1.74 V, as presented in Figure 5a. The values of *R*_ct_ were extracted from the equivalent circuit (Table S4) and plotted against the applied potential in Figure 5b.

**Figure 4:**
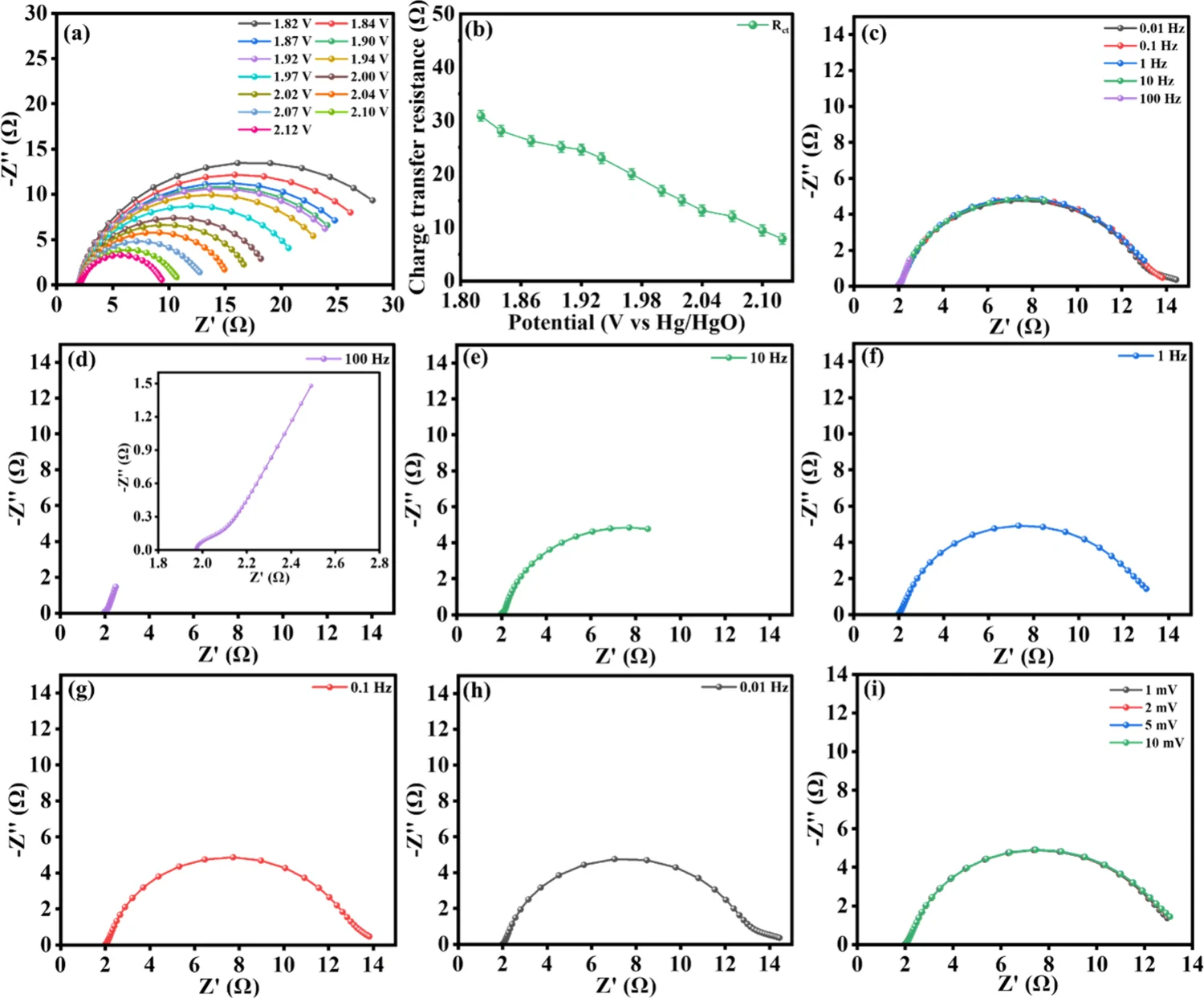
(a) Nyquist plot of CC with potential variation. (b) Variation in *R*_ct_ with applied potential of CC. (c) Nyquist plot of CC with frequency variation at 2.07 V vs Hg/HgO. (d–h) Nyquists plot of CC at 2.07 V vs Hg/HgO in frequency ranges of (d) 10^5^ to 100 Hz, (e) 10^5^ to 10 Hz, (f) 10^5^ to 1 Hz, (g) 10^5^ to 0.1 Hz, and (h) 10^5^ to 0.01 Hz. (i) Impact of amplitude variation on the Nyquist plot of CC at 2.07 V vs Hg/HgO and 10^5^ to 1 Hz.

**Figure 5:**
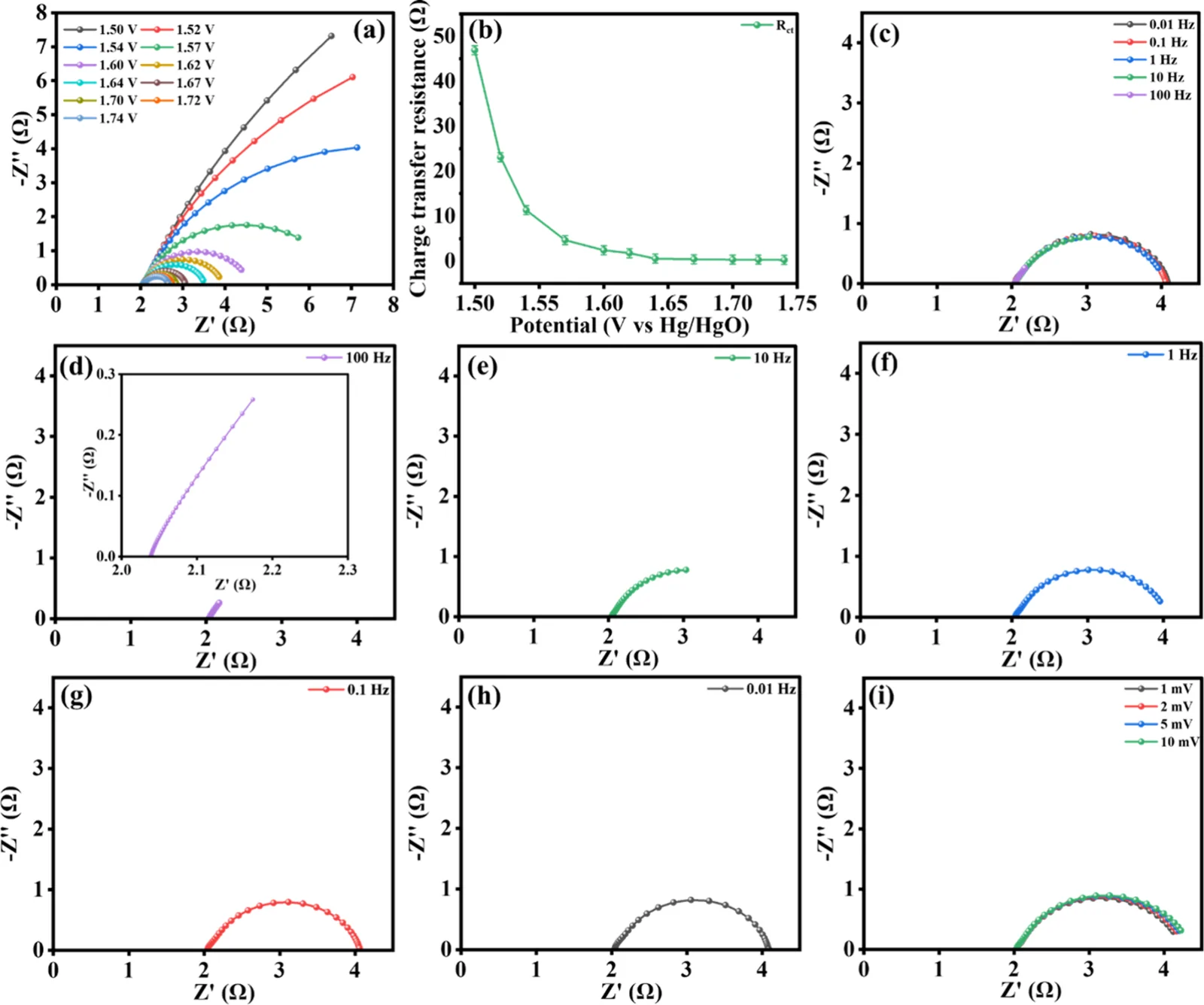
: (a) Nyquist plot of NF with potential variation. (b) Variation in *R*_ct_ with applied potential of NF. (c) Nyquist plot of NF with frequency variation at 1.70 V vs Hg/HgO. (d–h) Nyquist plots of NF at 1.70 V vs Hg/HgO in frequency ranges of (d) 10^5^ to 100 Hz, (e) 10^5^ to 10 Hz, (f) 10^5^ to 1 Hz, (g) 10^5^ to 0.1 Hz, and (h) 10^5^ to 0.01 Hz. (i) Impact of amplitude variation on the Nyquist plot of NF (at 1.70 V vs Hg/HgO and 10^5^ to 1 Hz).

#### 3.2.2 Impact of the Frequency Range

The next altering parameter of the EIS plot is the frequency range. To understand the impact of the frequency range, we have considered various frequency ranges: 10^5^ to 100 Hz, 10^5^ to 10 Hz, 10^5^ to 1 Hz, 10^5^ to 0.1 Hz, and 10^5^ to 0.01 Hz. The fitted EIS plots of CC at different frequency ranges (at 2.07 V vs Hg/HgO and 5 mV amplitude) are presented in Figure 4c. The fitted elements along with the equivalent circuit are presented in Table S2. Also, the EIS plots at different lower frequencies of 100, 10, 1, 0.1, and 0.01 Hz are presented separately in Figure 4d–h to clearly distinguish the plots. The selected lower frequency of 100 Hz gives information about solution resistance and capacitive elements only. It does not provide information about charge transfer resistance between the electrode and the electrolyte. The change of the lower frequency from 100 to 10 Hz provides some information about charge transfer resistance, yet not completely. Further, changing frequency from 10 to 1, 0.1, and 0.01 Hz provides appropriate information about all the components involved in the electrochemical process. Similarly, for NF, the EIS plot with variation of frequency range is recorded and presented in Figure 5c–h and Table S5. These EIS plots also depicted similar behavior as shown by CC. The frequency regions can be broadly classified into three regions:

1. **High Frequency Region (1 MHz to 100 Hz**)**:** In this frequency region, the impedance is dominated by the resistance of electrolyte (*R*) and the capacitive behavior of the electrode–electrolyte interface. This region is crucial to get the value of *R* used for *iR* compensation.
2. **Mid Frequency Region (100 Hz to 1 Hz**)**:** This region gives information about charge transfer resistance (*R*_ct_) and hence the kinetics of the reaction. The value of *R*_ct_ should be minimal for the reaction to proceed with faster kinetics. The catalytic activity of two catalysts can be analyzed in this region in terms of *R*_ct_.
3. **Low Frequency Region (1 Hz to 0.01 Hz):** In this frequency region, the impedance is dominated by the diffusion of ions or molecules between electrode and electrolyte. It gives information about mass transfer limitations or diffusion processes in terms of Warburg impedance, often used in batteries or fuel cells.

Thus, the EIS spectrum in EWS should be acquired in an appropriate frequency range from higher (≥10^5^ Hz) to lower (≤1 Hz) to probe all the possible elements involved in any reaction.

#### 3.2.3 Impact of AC Wave Amplitude

The next question that comes to our mind is the following: what should be the amplitude of the AC wave? As mentioned earlier, the EIS approach uses a small-amplitude stimulus (voltage or current) applied to an electrochemical system, typically overlaid on a dc signal (voltage or current), and measures the response (voltage or current, respectively) throughout a broad frequency range. A small-amplitude perturbation must be performed during measurements to guarantee a linear relationship between the applied signal and the system’s response, i.e., the input and output signals ought to use the same frequency. We have chosen different small amplitudes (1, 2, 5, and 10 mV) of AC frequency for perturbation to check how the EIS plot changes with amplitude. The EIS plots at different amplitudes at a fixed potential of 2.07 V vs Hg/HgO (for CC) or 1.70 V vs Hg/HgO (for NF) and frequencies of 10^5^ to 1 Hz are presented in Figure 4I for CC and Figure 5I for NF. The corresponding fitted equivalent circuits are presented in Table S3 and S6 for CC and NF, respectively. The Nyquist plot at all small amplitudes exhibits almost similar behavior for both CC and NF, exhibiting linearity of the system due to the fact that linear system’s impedance is independent of their amplitude.^18^ The majority of electrochemical systems typically behave nonlinearly unless small-amplitude perturbation signals are employed.

### 3.3 Tafel Slope

A popular metric for evaluating the rates and processes of electrocatalytic reactions is the Tafel slope method. According to its most basic definition, it is the potential required in mVs to increase the current by a factor of 10, so it is expressed in mV/dec Therefore, the activity of any catalyst is inversely proportional to the Tafel slope. The origin of the Tafel slope is from the famous Tafel equation (eq 29) given by Julius Tafel in 1905, often known as “the first law of electrode kinetics”:^16,56^

29 η=blog|j|+a

where *b* denotes the Tafel slope, η stands for overpotential, *a* is the intercept or Tafel constant, and *j* is the current density. The Tafel equation is derived from the Butler–Volmer equation (eq 30), which gives the current–potential relationship for a redox couple:^57,58^

30 $$j=j_{0}\left[-e^{-\alpha nF\eta /RT}+e^{\left(1-\alpha \right)nF\eta /RT}\right]$$

where, *j*_0_ is the exchange current density at zero overpotential, α is the charge transfer coefficient, *n* is the number of electrons transferred for oxidation/reduction, *F* is Faraday’s constant (96485 C mol^–1^), *R* is the gas constant (8.314 J K^–1^), and *T* is the temperature in K. At higher overpotentials (η ≫ 0) and *E* ≫ *E*_rev_, eq 30 reduces to the Tafel equation (eq 34) for the oxidation process as follows:

31 $$j=j_{0}\left[e^{\left(1-\alpha \right)nF\eta /RT}\right]$$

32 $$\frac{j}{j_{0}}=e^{\left(1-\alpha \right)nF\eta /RT}$$

33 $$\ln j-\ln j_{0}=\frac{\left(1-\alpha \right)nF\eta }{RT}$$

34 $$\eta =-\frac{2.303RT}{\left(1-\alpha \right)nF}\log j_{0}+\frac{2.303RT}{\left(1-\alpha \right)nF}\log j$$

Similarly, for η ≪ 0 and *E* ≪ *E*_rev_, eq 30 reduces to eq 38:

35 $$j=j_{0}\left[-e^{-\alpha nF\eta /RT}\right]$$

36 $$\frac{j}{j_{0}}=-e^{-\alpha nF\eta /RT}$$

37 $$\ln\left(-j\right)-\ln j_{0}=-\frac{\alpha nF\eta }{RT}$$

$$\eta =\frac{2.303RT}{\alpha nF}\log j_{0}-\frac{2.303RT}{\alpha nF}\log\left(-j\right)$$ 38

For simplification, eqs 34 and 38 can be written as

39 η=a±blog⁡j

where the intercept *a* is $\frac{2.303RT}{\alpha nF}\;\log j_{0}$ , which can be used to calculate the exchange current density, and the value of *b* can be determined by calculating the slope of the plot of η versus log *j* and is equivalent to $\frac{2.303RT}{\alpha nF}$ , which can be further used to calculate the charge transfer coefficient. However, it is not always easy to determine α, especially when *n* is unknown. However, it is extremely unlikely that many electrons will be transferred in the same elementary step, according to the absolute rate theory of electron transfer, also known as the Marcus hypothesis.^59,60^ Additionally, according to Bard and Faulkner,^300^ it is often assumed that electrons are transmitted one at a time when analyzing electrode kinetics. This is likely based on the theory that electrons transfer by tunneling, and since the tunneling probability is low, it is very unlikely (the probability is extremely low) that two electrons will tunnel simultaneously. Furthermore, fractional transmission of electrons is impossible. Therefore, it is fair to assume that *n* is always 1. If there are chances for the uncertain value of *n*, then the product of *n* and α can be reported to describe catalytic efficiency.^61^

There are several methods through which a Tafel plot can be constructed, including (1) voltammetry techniques (LSV or CV), (2) the EIS technique, or (3) the chronopotentiometry (CP) or chronoamperometry (CA) technique.

#### 3.3.1 Tafel Slope Using Voltammetry

The most common method followed by the majority of researchers is the voltammetry technique. However, as highlighted elsewhere, the Tafel determination from the LSV/CV, which is essentially a potentiodynamic transient approach, does not provide us with reliable data and is not advisible to utilize. The potential ramping from low to high, regardless of scan rate, produces a double-layer charging current in addition to the Faradaic current associated with a reaction. Tafel analysis should only take into account the current density corresponding to Faradaic reactions, such as OER/HER, etc., as it provides information on kinetics. In LSV/CV experiments, it is impossible to exclude the double-layer charging current and the metal oxidation/reduction current. To explore these possibilities, we have calculated the Tafel slopes of pristine CC and NF substrates from LSV recorded at 2 mV/s (100% *iR*-corrected manual) as shown in Figures 6a and 7a, respectively. The CC does not show a completely linear region in the current density range of 1–10 mA/cm^2^ (Figure 6a). This situation arises when the free energy of the first transition state is lower than that of the second.^62^ Thus, we fit two Tafel slopes in the linear region. For catalysts involving more than one Tafel slope, Tafel plots acquired experimentally in the Tafel regime do not reveal the chemical nature of the reaction intermediates; DFT is necessary for it. However, for NF, a linear region is observed for the chosen current range, which results in a single Tafel slope value of 59 mV/dec.

**Figure 6:**
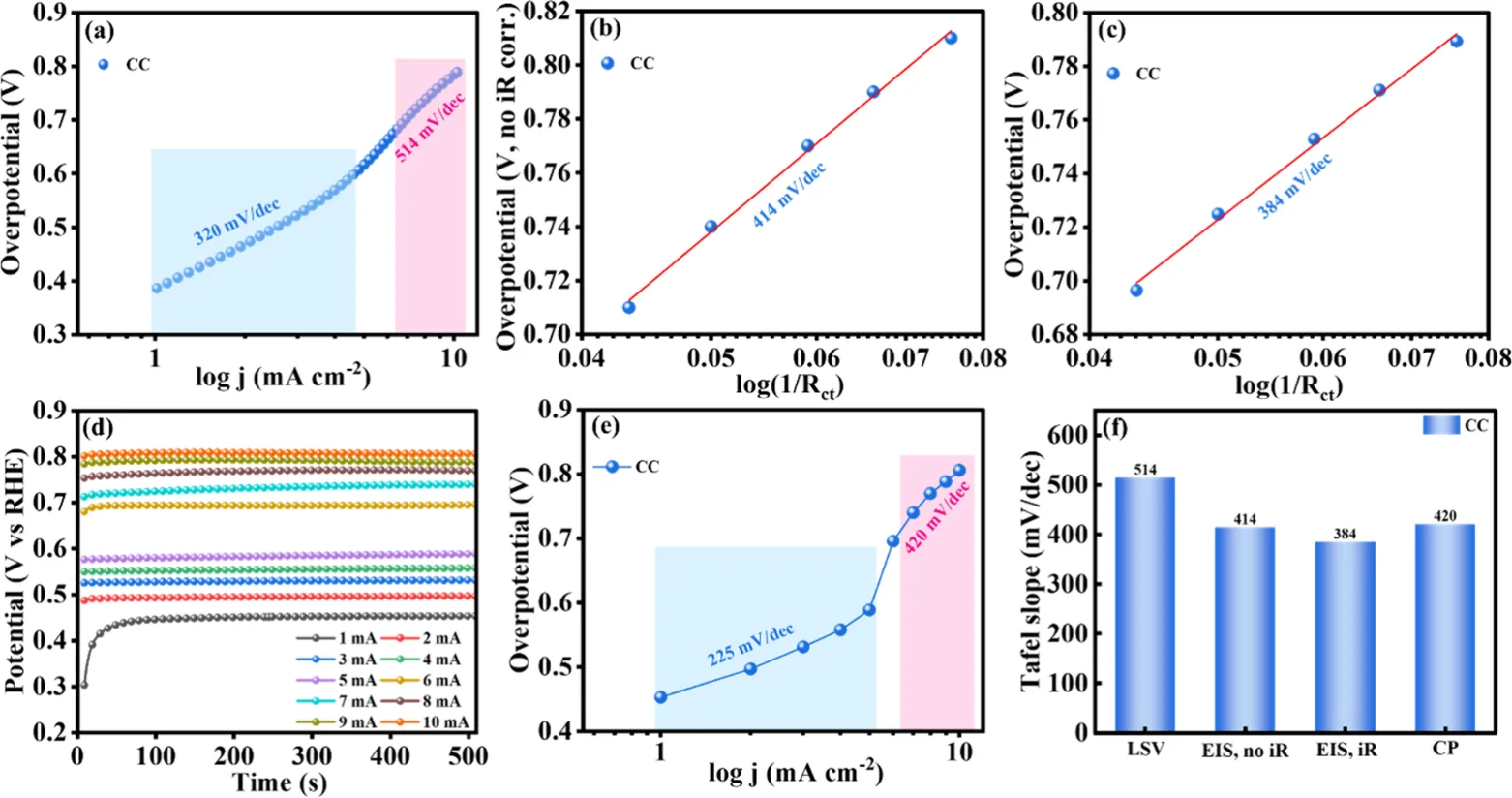
Tafel plot of CC obtained from (a) LSV method (b) EIS method without *iR* compensation, (c) EIS method with *iR* compensation, (d) Chronopotentiometry at different current density (1–10 mA cm^–2^), (e) Tafel plot from CP technique, and (f) comparison of Tafel slopes obtained through different methods for CC.

**Figure 7:**
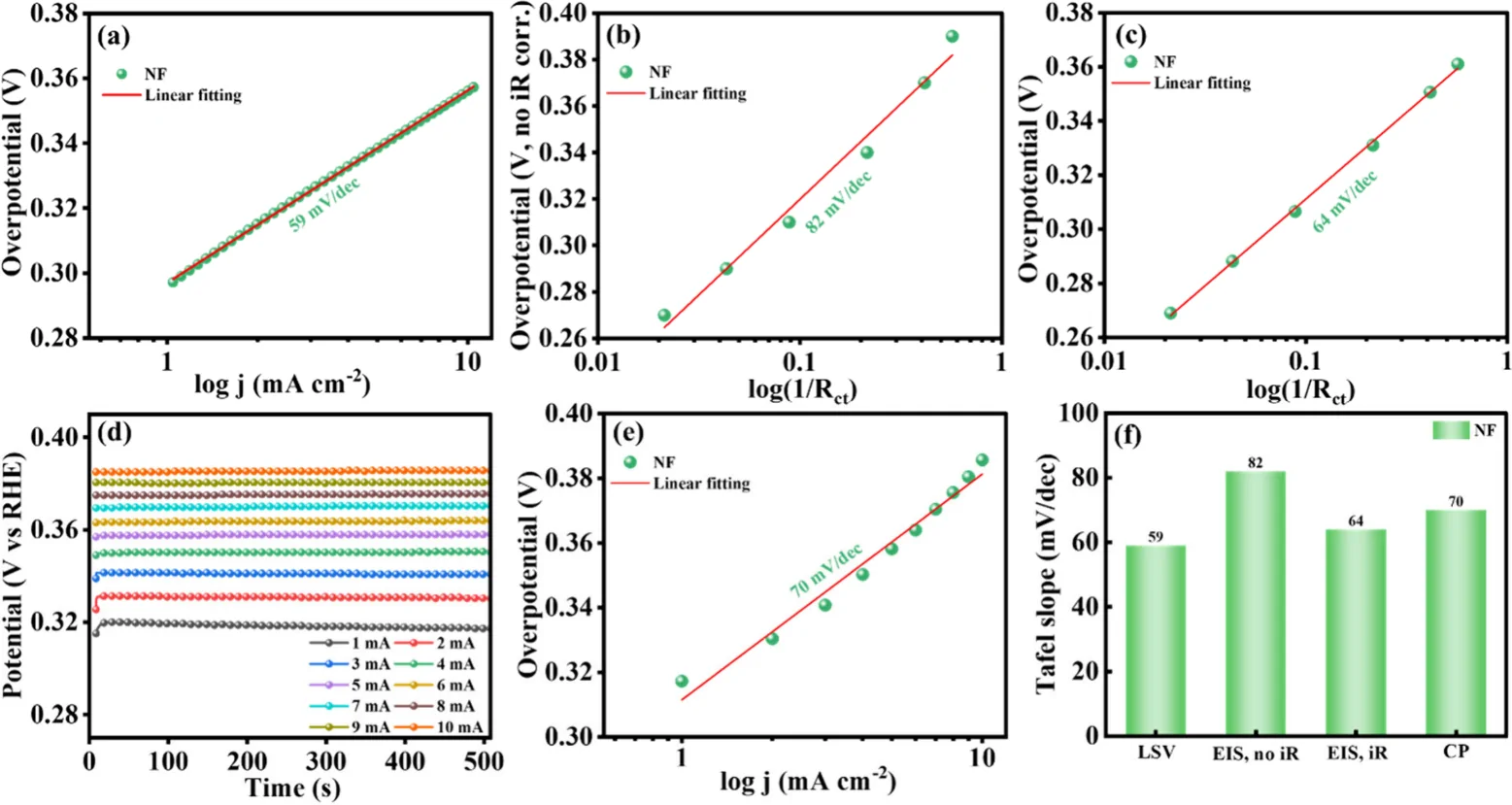
Tafel plot of NF obtained from (a) LSV method (b) EIS method without *iR* compensation, (c) EIS method with *iR* compensation, (d) Chronopotentiometry at different current density (1–10 mA cm^–2^), (e) Tafel plot from CP technique, and (f) comparison of Tafel slopes obtained through different methods for NF.

#### 3.3.2 Tafel Slope Using EIS

EIS can be an alternate technique used for the Tafel plot involving log­(1/*R*_ct_) versus η. This technique uses the potential to provide the benefit of steady-state measurements. But this technique is based on the assumption that of the current density behavior is thought to be exactly equal to 1/*R*_ct_. For an electrochemical reaction involving current density (*j*) and overpotential (η), one can write^63^

40 $$\eta =jR_{\mathrm{ct}}$$

If we take the first derivative of eq 40 with respect to η, then it transforms as follows:

41 $$1=R_{\mathrm{ct}}\frac{dj}{d\eta }+j\frac{dR_{\mathrm{ct}}}{d\eta }$$

42 $$1=R_{\mathrm{ct}}\frac{dj}{d\eta }$$

43 $$\frac{1}{R_{\mathrm{ct}}}=\frac{dj}{d\eta }$$

This equation is valid only when either *j* = 0 or *R*_ct_ is overpotential-independent (i.e., constant). Since *i* can be near zero at very low overpotential and *R*_ct_ is potential-dependent, the equation is only true for low overpotentials, which is its disadvantage.

Further, the Tafel slope is obtained from the EIS approach. The value of *R*_ct_ is obtained by fitting the Nyquist plot with the equivalent circuit. As the Nyquist plot does not provide information about current density, *iR* compensation is not possible. Figures 6b and 7b show the Tafel plots obtained from EIS without *iR* correction for CC and NF, respectively. CC shows the Tafel slope value of 414 mV/dec, which is lower than the Tafel slope obtained from LSV (514 mV/dec), and NF shows a value of 82 mV/dec, which is higher than that from LSV (59 mV/dec). These discrepancies can be resolved by applying *iR* compensation in the Tafel plot obtained from the EIS method. This can be done by first recording the LSV spectrum and getting the current densities corresponding to the potentials at which EIS is recorded. The *iR*-corrected Tafel plots from the EIS method are presented in Figures 6c and 7c for CC and NF, respectively. Now, the Tafel slope value obtained for CC is 384 mV/dec, which is lower than that from the LSV method, and the value for NF is 64 mV/dec, which is close to that obtained from LSV.

#### 3.3.3 Tafel Slope Using CA/CP

Further, the CA/CP method is the most commonly used and precise way to measure Tafel analysis, as it allows measuring current density or potential in steady state. The *i*/*V* versus *t* graph for a straightforward reaction with no surface intermediate will be a straight line once double-layer charging is finished. However, in multistep complex reactions with several surface intermediates, the time it takes for the *i*/*V* versus *t* graph to show a straight line may rise as the intermediates are adjusted to a steady state. This method also possesses the disadvantage of creating gas bubbles during the investigation, which could obstruct the active surface of evolution reactions like the OER/HER, which could alter the reaction rate. Here, we performed step-chronopotentiometry measurements for current densities ranging from 1 to 10 mA/cm^2^ at a difference of 1 mA/cm^2^ between each step (Figure 6d for CC and Figure 7d for NF). The CP-derived Tafel plots are presented in Figures 6e and 7e for CC and NF, respectively. The Tafel slopes obtained from this method are 420 mV/dec for CC and 70 mV/dec for NF. The comparison of Tafel slope values (mV/dec) obtained from different methods is presented in Figure 6f for CC, which follows the trend 514 (LSV) > 420 (CP) > 414 (EIS, without *iR*) > 384 (EIS, with *iR*). However, for NF, the obtained trend is 82 (EIS, without *iR*) > 70 (CP) > 64 (EIS, with *iR*) > 59 (LSV) (Figure 7f).

Thus, it is quite clear that different methods give different Tafel slope values, and different trends for different catalysts. Thus, it is hard to conclude which method is best. Every method has its pros and cons. For comparing two catalysts, there would be no requirement for a relative comparison if two axes were fixed, because each Tafel slope value would have the same inaccuracy. Consequently, the method used to determine the Tafel slope value does not change the findings, which is why static approaches are not common in practice. But it is believed that the CP/CA technique is a better way to get the kinetics of the reaction in terms of the Tafel slope value, as it represents steady state, which is essential to know the kinetics. In contrast, the potential in LSV/CV varies before the intermediate radical accumulation is completed, leading to a larger fluctuation in the current density than in the steady state. Because of these factors, the Tafel slope from LSV/CV typically produces a low or high Tafel slope value (depending on the electrodes and the kind of reaction being studied), indicating low or fast kinetics in contrast to genuine kinetics, which is deceptive. Also, since the current is not zero at high overpotential, d*i*/dη is not equal to 1/*R*_ct_. Therefore, it is fundamentally incorrect to calculate the Tafel slope from the EIS, and it is also very time-consuming. Keeping in mind the factors stated above, we recommend the CA/CP technique as a better method in comparison to the Tafel slope obtained from the LSV/CV (recorded at scan rate less than or equal to 2 mV/s) method for getting true kinetics.

### 3.4 Electrochemical Active Surface Area (ECSA)

It is an important parameter that is directly related to the better catalytic performance of any electrocatalyst. The surface area exposed to the electrolyte is directly correlated with the electrical current observed when sweeping the electrode potential. An increased exposed area of a catalyst available for interaction with electrolyte ions is indicated by a high ECSA value and hence resulting in higher catalytic activity. The geometrical surface area (*A*_geo_) of the working electrode is entirely different from the exposed area. Generally, the ECSA is much higher than the *A*_geo_ as the surface area of catalysts increases through nanoengineering, resulting in a significantly different electrochemically active region from the flat electrode’s geometrical area.^64^ The most widely adopted method for ECSA determination is the double-layer capacitance (*C*_dl_) method in the non-Faradaic region. Now, the question comes: what is a non-Faradaic region, and why is it used to calculate ECSA? The potential region/window in which only adsorption–desorption takes place, but no charge transfer occurs, or no redox reactions occur, is identified as the non-Faradaic region.^65^ This is chosen because the applied potential in this region is not sufficient to drive any oxidation or reduction processes, so the current is purely capacitive, which arises due to the charging/discharging of the electrical double layer at the electrode–electrolyte interface. However, in Faradaic regions, the current is contributed from the capacitive nature as well as redox reactions or any other side reactions. Thus, isolating purely capacitive current is very challenging and almost impossible. To calculate ECSA, first, we have to identify the potential window, i.e., the lower and higher potentials at which CV cycling is to be done. This can be determined from LSV/CV, as shown in Figure 8a for CC and Figure 8d for NF. The working electrode CC shows a large non-Faradaic region in comparison to NF. So, different catalysts possess different non-Faradaic regions. Generally, the low potential regions (0–0.1 V, 0.1–0.2 V, etc.) can be considered for CV cycling.

**Figure 8:**
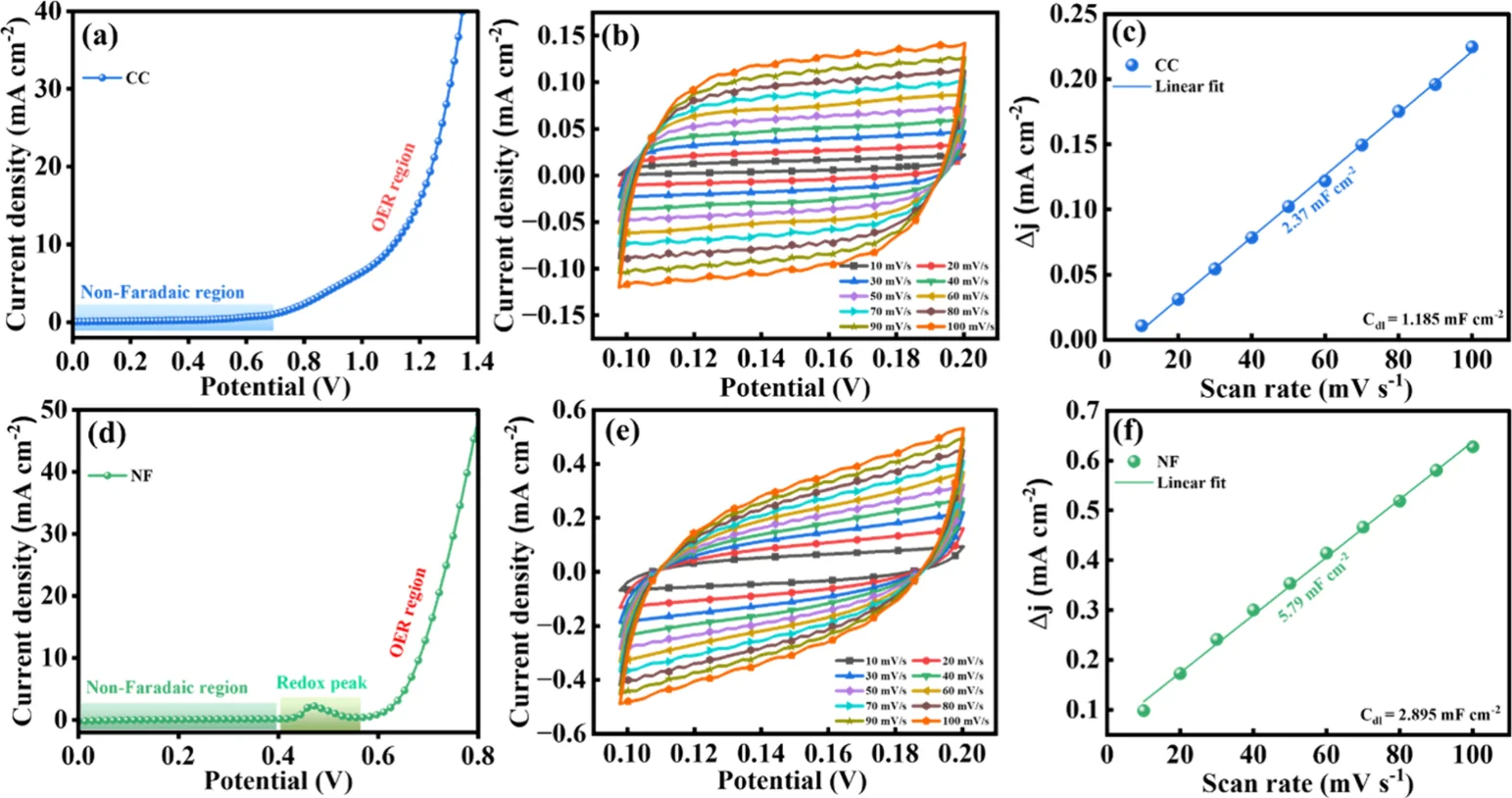
(a) Identification of the non-Faradaic region for CC. (b) CV cycles with variable scan rates for CC. (c) Plot of capacitive current vs scan rate for CC. (d) Identification of the non-Faradaic region for NF. (e) CV cycles with variable scan rates for NF. (f) Plot of capacitive current vs scan rate for NF.

In non-Faradaic regions, CV cycles are collected at various scan rates to calculate a catalyst’s *C*_dl_ value. The slope is then calculated by graphing the difference between the anodic and cathodic current densities (Δ*j*) versus scan rate (υ), which directly correlates with the *C*_dl_ value, as shown in eqs 44–48. The general equation for a capacitor, where the current is related to the time derivative of the voltage, is given in eq 44:

$$\Delta j\left(t\right)=C_{\mathrm{dl}}\frac{dV\left(t\right)}{dt}$$ 44

The scan rate is given by eq 45:

$$\upsilon =\frac{dV(t)}{dt}$$ 45

Δ*j*(*t*) can be calculated as follows from eqs 46–47:

$$\Delta j\left(t\right)=j_{anodic}-j_{cathodic}$$ 46

Thus,

$$\Delta j\left(t\right)=C_{\mathrm{dl}}\upsilon -(-C_{\mathrm{dl}}\upsilon )=2C_{\mathrm{dl}}\upsilon$$ 47

Thus, in the plot of Δ*j* versus υ, half of the slope gives the value of *C*_dl_. Further, the ECSA can be calculated from *C*_dl_ as follows from eq 48:

$$\mathrm{ECSA}\;=\mathrm{Geometrical}\;\mathrm{surface}\;\mathrm{area}\times \frac{C_{dl}}{C_{s}}$$ 48

where C_s_ is the specific capacitance of the electrolyte (0.04 mF/cm^2^ for 1 M KOH, 0.035 mF/cm^2^ for 1 M H_2_SO_4_).^66^ The CV cycling with various scan rates from 10 to 100 mV/s with a successive difference of 10 mV/s is presented in Figure 8b for CC and Figure 8e for NF. The plots of capacitive current with various scan rates at the middle of the potential range are shown in Figure 8c for CC and Figure 8f for NF. The calculated *C*_dl_ for CC is 1.185 mF cm^–2^, and hence, ECSA is 29.625 cm^2^, which is much higher than *A*_geo_ (1 cm^2^). Similarly, for NF, the calculated *C*_dl_ is 2.895 mF cm^–2^, and hence, ECSA is 72.375 mF cm^–2^, which is much higher than that of the CC electrode due to the 3D foam-like structure.

Still, there are several controversies. For example, Jeon et al.^67^ demonstrated that the catalysts in the case of Ni-based hydroxides are nonconductive at the non-Faradaic potential zone prior to the Ni­(II) oxidation, which prevents precise electrochemical measurements. Therefore, it is suggested to use electrochemical impedance spectroscopy (EIS) at the OER active potential to determine the adsorbate capacitance (*C*_a_) resulting from OER intermediate species (O*, OH*, OOH*) on the NiFe LDH active sites.^68,69^ This approach is advantageous because it measures the capacitance during the reaction, when the catalyst is electrically conductive, and it only accounts for the capacitance from the catalyst’s active component rather than the support material. The value of *C*_a_ determined by EIS fitting at a selected range of OER active potential was then divided by the specific capacitance (*C*_s_) to get the ECSA of the catalysts. Mathematically, *C*_a_ can be written as^70^

49 $$C_{a}=Q\frac{d\theta }{dV}$$

where *Q* is the charge of the complex adsorbate layer, θ is the surface coverage fraction of OER intermediates, and *V* is the applied potential. Thus, we can use this method for Ni-, Co-, Fe-, and Ir-based hydroxides to get more accurate ECSA values, as demonstrated by Watzele et al.^70^

### 3.5 Turnover Frequency (TOF) Calculation

Turnover frequency is the basic intrinsic activity marker that measures the quantity of product formed or reactant consumed per unit time for a given amount of catalyst. It is defined as the number of moles of H_2_ or O_2_ evolved per unit time. This allows one to estimate the catalyst’s efficiency for a specific reaction.^71,72^ It is most commonly calculated by eq 50:

50 $$\mathrm{TOF}=\frac{jN_{A}}{nF\Gamma }$$

where, *j*, *n*, Γ, and *N*_A_ represent the current density at the overpotential value at which TOF is calculated, the number of electrons transferred, the number of electrochemically accessible sites (ECAS), and the Avogadro constant (6.022 × 10^23^ mol^–1^), respectively. Here, one should note that ECSA is different from ECAS, as ECSA measurements reveal how many electrochemically active sites are present on the electrodes, but there is a probability that not all of these sites are catalytically active. There are several methods for calculating ECAS, including the traditional theoretical method, underpotential deposition (UPD), stripping methods, and redox peak integration.^20^ Regrettably, these techniques are highly material-specific and cannot be applied universally to all types of electrocatalysts. The traditional theoretical method assumes that all active sites participate in both HER and OER. The number of surface sites per unit area for a catalyst can be calculated as

51 $$n_{\mathrm{surface}\;\mathrm{sites}}={\left(\frac{x}{V_{\mathrm{unit}\;\mathrm{cell}}/N_{A}}\right)}^{2/3}$$

where *x* is the number of atoms per formula unit cell and *V*_cell_ is the unit cell volume (in cm^3^/mol), given by

52 $$V_{\mathrm{unit}\;\mathrm{cell}}=\frac{\mathrm{FW}}{\rho }$$

in which FW is the formula weight (in g/mol) and ρ is the density (in g/cm^3^).

The UPD method is a voltammetric technique that integrates the area under the *I*–*V* spectrum in the UPD region to determine the charge needed to electrochemically adsorb or desorb an adlayer of a selected electrocatalyst. For example, hydrogen underpotential deposition (HUPD) is the most commonly used method for ECAS measurements of Pt-based electrocatalysts. Unfortunately, polycrystalline nanostructured electrocatalysts with one or two prominent facets are widely adopted as catalysts these days. In these circumstances, assuming a set value of UPD charges established for certain planes would result in a very erroneous calculation of the real surface area.^73,74^ Furthermore, the catalytic electrodes are known to be strongly poisoned by carbon monoxide due to its high affinity for many metals and noble metals. However, this unfavorable adsorption of CO can be used to determine the ECAS of solid electrodes. CO adsorbs heavily on all noble metals and their alloys in the potential range that corresponds to the double layer and hydrogen regions.^75^ If a high enough potential is applied to the electrode, it can be completely desorbed by oxidizing to CO_2_. The surface area may be calculated by integrating the CO oxidation peak, and it can be converted to the ECSA using a conversion factor of 420 μC cm^–2^, as it involves two-electron oxidation.^76^ However, there are still problems of metal dissolution from the electrode surface that may accompany adsorbed CO oxidation at high potentials, changing the electrode surface state and, in the case of alloys, surface composition.^77^

The redox-peak integration approach is another simpler way to examine ECAS of transition metals that possess a redox couple. The process of electrocatalytic water oxidation requires the catalytic sites to go through a full cycle of oxidation and reduction, which includes intermediate bond-forming and bond-breaking stages. Therefore, it is reasonable to infer that the precise number of sites involved in the reaction is the number of metal sites undergoing oxidation before water oxidation. The precise number of catalytic sites can be determined by calculating the total number of electrons transferred based on the area under the peak, provided that the number of electrons transferred in the oxidation/reduction of a single site is known. Mathematically, it can be calculated as follows. Let *A*_ox/red_ be the area under the peak associated with the oxidation/reduction of the transition metal (in mA·V), and let υ be the scan rate (in mV·s^–1^). Then the associated charge *q* (in A s^–1^ = C) is given by

$$q=\frac{A_{\mathrm{ox/red}}}{\upsilon }$$

The number of electrons transferred is then obtained by dividing this total charge *q* by the charge per electron:

$$n_{\mathrm{transferred}}=\frac{q}{e}$$

where *e* = 1.602 × 10^–19^ C is the elementary charge.

For a single-electron transfer reaction, the number of electrons calculated above is exactly the same as the number of surface-active sites.

Herein, based on CC and NF electrodes, we adopted two methods to calculate ECAS, as shown in the following sections.

#### 3.5.1 TOF Using the Theoretical Method

For CC, TOF can be calculated for OER as follows:

53 $$\mathrm{TOF}=\frac{n_{\mathrm{oxygen}\;\mathrm{turnovers}}}{n_{\mathrm{surface}\;\mathrm{sites}}}$$

where *n*_oxygen turnovers_ is the total number of oxygen molecule turnovers per unit area, which can be obtained from the current density (in mA/cm^2^) as shown below:

$$n_{\mathrm{oxygen}\;\mathrm{turnovers}}=j\;(mA/{cm}^{2})\cdot \left(\frac{1\;\mathrm{C/s}}{1000\;}\right)\left(\frac{1\;\mathrm{mol}\;\mathrm{of}\;e^{-}}{96485.3\;C}\right)\left(\frac{1\;\mathrm{mol}\;\mathrm{of}\;O_{2}}{4\;\mathrm{mol}\;\mathrm{of}\;e^{-}}\right)\left(\frac{6.022\times 10^{23}\;O_{2}\;\mathrm{molecules}}{1\;\mathrm{mol}\;\mathrm{of}\;O_{2}}\right)$$ 54

where the factor (1 mol of O_2_)/(4 mol of e^–1^) is due to the fact that OER is a four-electron process. Equation 54 simplifies to

$$n_{\mathrm{oxygen}\;\mathrm{turnovers}}=j\cdot (1.56\times 10^{15}\;O_{2}\;\mathrm{molecules}\;\cdot s^{-1})$$ 55

For CC, the formula weight is 12.011 g/mol, and the density is 2.26 g/cm^3^. Using these values in eq 52 gives

$$V_{\mathrm{unit}\;\mathrm{cell}}=\frac{12.011\;\mathrm{g/mol}}{2.26\;{\mathrm{g/cm}}^{3}}=5.314\;{\mathrm{cm}}^{3}/\mathrm{mol}$$

Substituting this value along with *x* = 1 atom per unit cell for CC into eq 51 gives the number of surface sites per unit area:

56 $$n_{\mathrm{surface}\;\mathrm{sites}}={\left(\frac{1}{\frac{5.314\;{\mathrm{cm}}^{3}\cdot {\mathrm{mol}}^{-1}}{6.022\times 10^{23}\;{\mathrm{mol}}^{-1}}}\right)}^{2/3}=2.342\times 10^{15}\;{\mathrm{cm}}^{-2}$$

Finally, substituting eqs 55 and 56 into eq 53 yields

$${\mathrm{TOF}}_{\mathrm{OER}}=\frac{j\cdot (1.56\times 10^{15}\;O_{2}\;\mathrm{molecules}\cdot s^{-1})}{2.342\times 10^{15}\;}=j\;\left({\mathrm{mA/cm}}^{2}\right)\cdot (0.666\;O_{2}\;\mathrm{molecules}\cdot s^{-1})$$ 57

The calculated TOFs at different overpotentials for CC are presented in Figure 9a.

**Figure 9:**
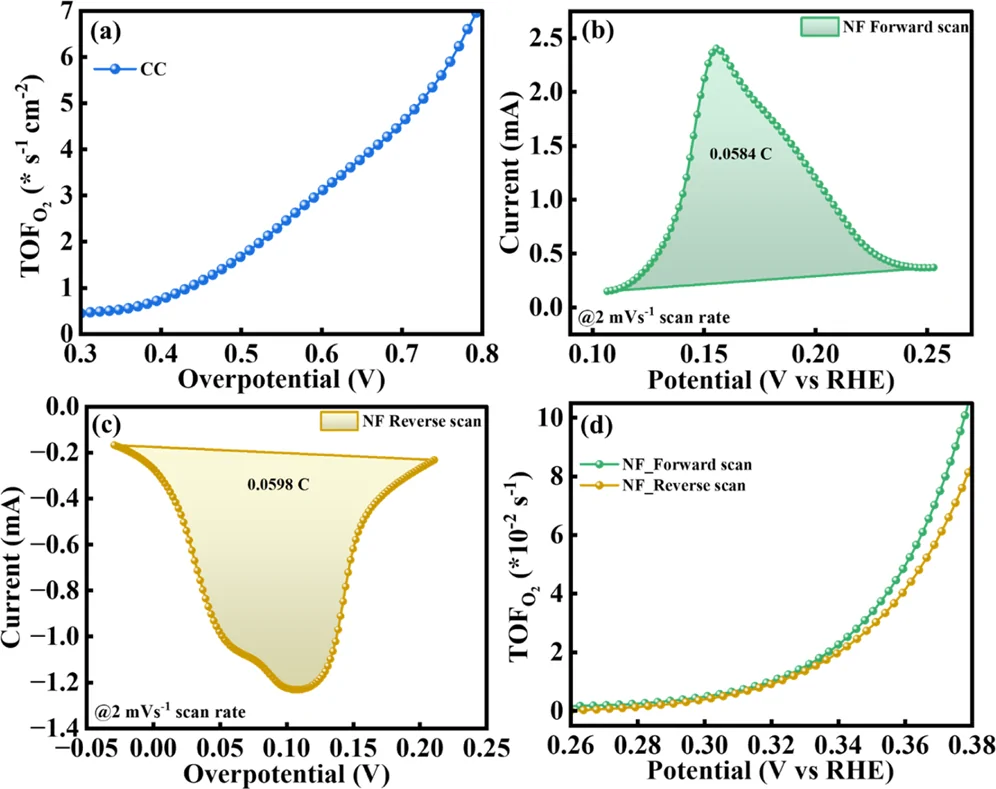
(a) O_2_ TOFs of CC at various overpotentials. (b) Oxidation peak area of Ni^2+^ to Ni^3+^ for NF. (c) Reduction peak area of Ni^3+^ to Ni^2+^ for NF. (d) TOFs of NF at various overpotentials in forward and reverse scans for O_2_.

#### 3.5.2 TOF Using the Redox-Peak Integration Method

For NF, the redox-peak integration method can be used, as it involves the Ni^2+^ to Ni^3+^ oxidation peak in the forward scan. The reverse scan of LSV can also be used to calculate the number active sites, as it involves the Ni^3+^ to Ni^2+^ reduction peak. The calculation for ECAS of NF can be done as follows by the integration of the area under the peak, as shown for oxidation in Figure 9b and for reduction in Figure 9c. It has been noticed that NF shows almost similar peak areas for the oxidation and reduction peaks, indicating its redox reversibility. Further, to estimate the exact number of ECAS, the following steps can be adopted:

The area associated with the reduction and oxidation of Ni was found to be *A*_red_ = 0.1196 mA.·V, and *A*_oxd_ = 0.1167 mA.·V. The scan rate was 2 mV s^−1^. Thus, the associated charge *q*_red_ = *A*_red_/*v* = 0.0598 C and *q*_oxd_ = *A*_oxd_/*v* = 0.0584 C. Hence, the number of electrons transferred is given by *n*_*red transferred*_ = *q_red_*/e = 3.7375 × 10^17^ and *n_oxd transferred_* = *q_oxd_*/e = 3.65 × 10^17^. Since the oxidation of Ni^2+^ to Ni^3+^ and reduction from Ni^3+^ to Ni^2+^ are single-electron-transfer reactions, the number of electrons calculated above is exactly the same as the number of surface active sites. Hence, the number of Ni sites that participated in the reduction reaction is 3.7 × 10^17^ and in oxidation reaction is 3.65 × 10^17^.

The TOF variation with overpotential calculated from eq 50, of NF for OER is presented in Figure 9d for both the forward and reverse scans. As NF contains equal ECAS for both oxidation and reduction peaks, there are still differences in TOF for forward and reverse scans. This arises from the current density difference, as the forward scan shows higher current due to extra oxidation current involvement that can affect TOF as well. Thus, one can use a reverse scan of LSV for a more accurate determination of TOF.

### 3.6 Stability (Chronoamperometry versus Chronopotentiometry)

The evaluation of an electrocatalyst’s stability over an extended period of operation is a critical factor in determining its feasibility for large-scale commercial operations. Typically, cyclic voltammetry (CV), chronoamperometry (CA), and chronopotentiometry (CP) are used as complementary techniques to assess the stability of electrocatalysts.^78^ For the first method, by performing continuous CV scans (e.g., more than 1000 scans) in a typical HER/OER potential range, one can learn about the electrocatalyst’s stability by comparing the first and last scans. The electrocatalyst’s stability is indicated by changes in the overpotential at the same current density or vice versa. The stability is generally higher when the change is smaller. CV assesses the stability under transient situations in a comparatively broad range of overpotentials and current densities.^79^ For the second method, CA monitors changes in current density over time at a fixed potential for several hours/days. The stability of the catalyst in this case is provided by measuring the difference between the initial and final current densities. Furthermore, CA assesses stability at a fixed potential, indicating a stationary electrooxidation or electroreduction state.^80^ For the third method, in CP the change in potential is monitored over time at a constant current density. The stability of the catalysts is indicated by measuring the change in overpotential for the same current density as before. The potential changes during CP measurement suggest different electrooxidation or electroreduction conditions. Additionally, more active catalysts have a lower overpotential or less electrooxidation condition during CP studies at the same current density, which could result in improved stability.^81^

For industrial realization, CA and CP are preferred over CV cycle stability. The CV cycles can be used to show on–off stability of the system, whereas CA/CP enables one to monitor constant activity over time. Thus, CV cyclic stability can be used to show additional stability with CA/CP. But the representation of CV alone is not preferred for reporting industrially viable, stable electrocatalysts. Now, the next question is CA versus CP, which is best? To answer this question, we have conducted both CP and CA measurements for CC and NF for an identical time period. The percentage retention in activity is the best way to demonstrate stability. But do not rely on these numbers so often. These numbers can vary from CP to CA measurements for the same electrocatalyst. Nonetheless, these numbers also vary in different CP techniques of data representation, i.e., one can represent CP data in terms of the obtained potential (V) without including any correction with respect to the reference electrode and *iR* compensation, or one can represent it by including correction with respect to the reference electrode but without *iR* compensation (denoted as V vs Hg/HgO), or one can represent it by including both corrections (denoted as V vs RHE). Here we present the obtained V, V vs Hg/HgO, and V vs RHE CP data for CC in Figure 10a–c, respectively, and the results for CA measurements in Figure 10d. Surprisingly, the activity retention percentage varies for the same electrocatalyst depending on the different CP data representations and CA measurements, although the pristine CC substrate shows degradation in both measurements, as shown in the inset of Figure 10a. The color of the electrolyte changes to yellowish-brown from a clear and transparent KOH solution. Initially, we thought it was due to the graphite rod counter electrode. We then replaced it with a Pt wire counter electrode to investigate whether it would happen again, and we observed a similar color change. We also tested different CC purchased from the Fuel Cell store, and we still observed the same color change of the electrolyte after the stability test as we observed through the CC purchased from Nanoglobal Technologies.

**Figure 10:**
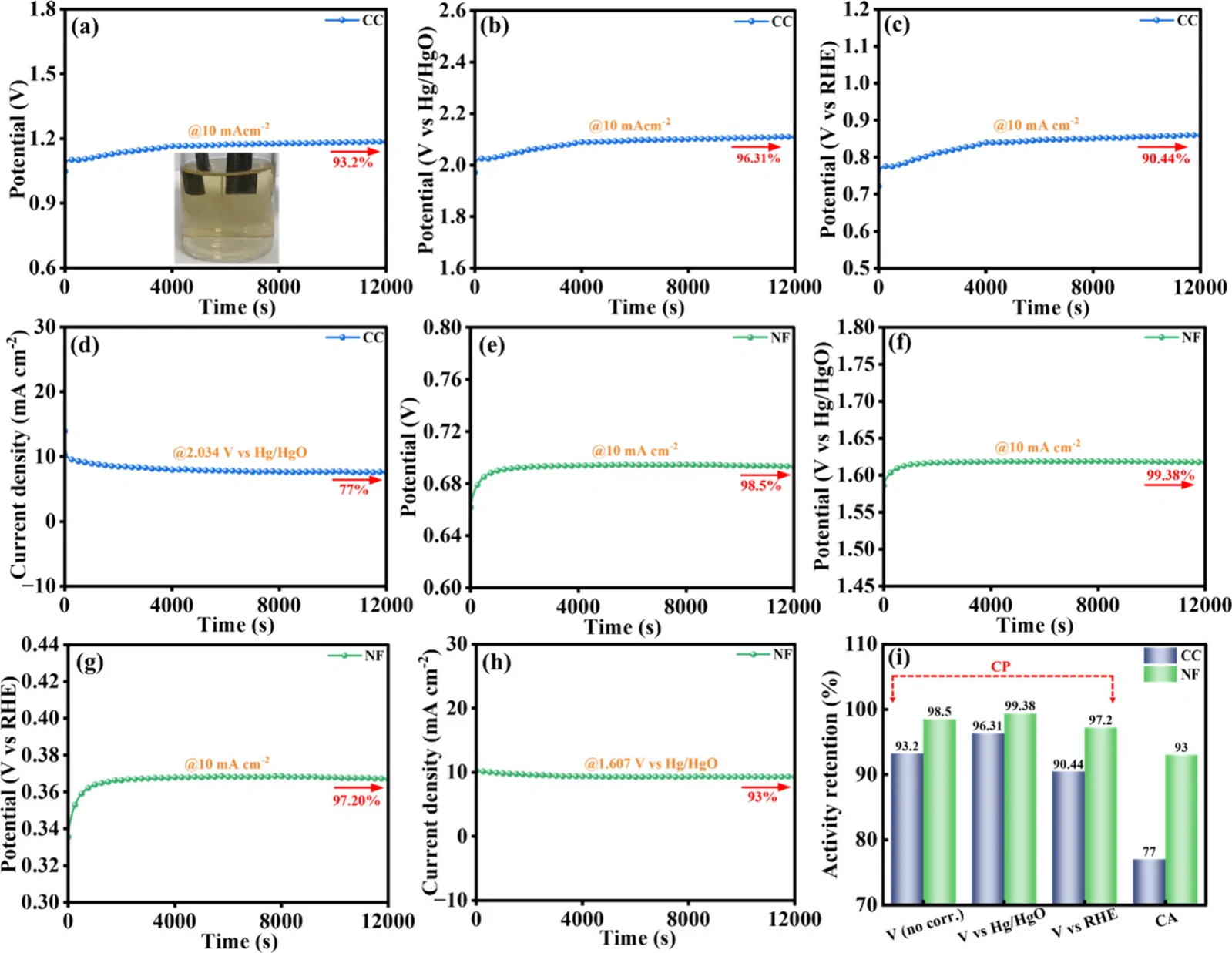
Chronopotentiometry of CC in terms of (a) as obtained V, (b) V vs Hg/HgO, and (c) V vs RHE. (d) Chronoamperometry of CC at 2.034 V vs Hg/HgO. (e–g) Chronopotentiometry of NF in terms of (e) as obtained V, (f) V vs Hg/HgO, and (g) V vs RHE. (h) Chronoamperometry of NF at 1.607 V vs Hg/HgO. (i) Comparison of activity retention (%) via CP and CA.

To check whether this discrepancy is applicable only to CC, we performed similar CP and CA measurements for NF as presented in Figure 10e–g and Figure 10h, respectively. Here also the same difference can be noticed. Hence, it has been observed that CP measurements always show higher retention activity than CA measurements. This difference in activity retention percentage is due to the relative dividing factor in the denominator. As can be expressed mathematically,

58 $$\mathrm{activity}\;\mathrm{retention}\;\mathrm{percentage}=\frac{\left|P_{0}-P_{F}\right|}{P_{0}}\times 100\%$$

where *P*_0_ denotes the initial current (*I*_0_) or voltage (*V*_0_) before testing the stability and *P*_F_ denotes the final current (*I*_F_) or voltage (*V*_F_) obtained after stability under the same conditions. To understand it more clearly with an example, suppose you are testing the CA measurements for a current of 10 mA, and after the stability test, the current is reduced to 9 mA. Then the activity retention percentage will be 90% even for just 1 mA current decay. Similarly, suppose you are testing CP measurements to observe a constant potential of 250 mV, and the potential changes to 260 mV after stability. Then the activity retention percentage will be 96%, even for 10 mV of potential rise. Thus, there are several complexities that one should notice to compare the stability retention activity while working with CP measurements, whereas it would be much easier to compare the CA measurements with other works, as shown in Figure 10i. Thus, we recommend that CA is better than CP for getting more reliable activity retention and a fairer comparison.

The performance of the electrocatalyst cannot be systematically represented by the change in current density or change in overpotential with time alone, as they only offer stability-related information. Therefore, descriptors combining stability and activity data, such as the activity-stability factor (ASF) and stability number (S-number), were proposed to allow for a fair comparison of various catalysts.^82^ The ratio of evolved O_2_/H_2_ molecules to dissolved active metal is known as the S-number, and it indicates how many molecules are generated for every atom of dissolved active metal in the electrolyte.^83^ Greater stability is indicated by a larger S-number. The ratio of the OER current to the corresponding metal dissolution current, as determined by ICP-MS studies, is known as the ASF.^84^ The lifetime of an electrocatalyst can be estimated using eq 59:

59 $$t=\frac{SnFm}{jM}$$

where *S* is the S-number, *n* is the number of electrons transferred, *F* is the Faraday constant, *m* is the mass of the catalyst loaded on the electrode, *j* is the current density, and *M* is the molar mass of the catalyst. The ASF can be evaluated using eq 60:^85^

60 $$ASF={\frac{J-J_{s}}{J_{s}}|}_{\eta }$$

where *J* is the total current density for the O_2_/H_2_ evolution and corresponding dissolution, *J*_s_ is the dissolution current density, and η is the constant overpotential.

### 3.7 From a Three-Electrode Test to an Alkaline Water Electrolyzer

In this section, we tried to highlight the changes that occur when transitioning from a three-electrode setup to a complete alkaline water electrolyzer. Among the various electrolyzers types, the alkaline water electrolyzer (AWE) has been extensively studied and commercially adopted due to its technological maturity, low material costs, and long operational life.^86^ However, conventional AWE systems suffer from performance limitations, especially due to the large interelectrode distance and high internal resistance caused by thick liquid electrolyte layers. To overcome these drawbacks and enhance energy efficiency, the zero-gap alkaline water electrolyzer (ZGAWE) design has emerged as a promising solution by minimizing the gap between the electrodes and the membrane.^87^ In this configuration, the anode, diaphragm (or separator), and cathode are in direct contact or placed with negligible spacing, effectively reducing ionic transport resistance and improving overall cell performance. The electrolyzer consists of several parts, such as end plates, gaskets, current collector plates, or flow plates, and a membrane electrode assembly (MEA) having anode and cathode catalyst separated with an anion exchange membrane (AEM). The schematic of the ZGAWE used in this study is presented in Figure 11a.

**Figure 11:**
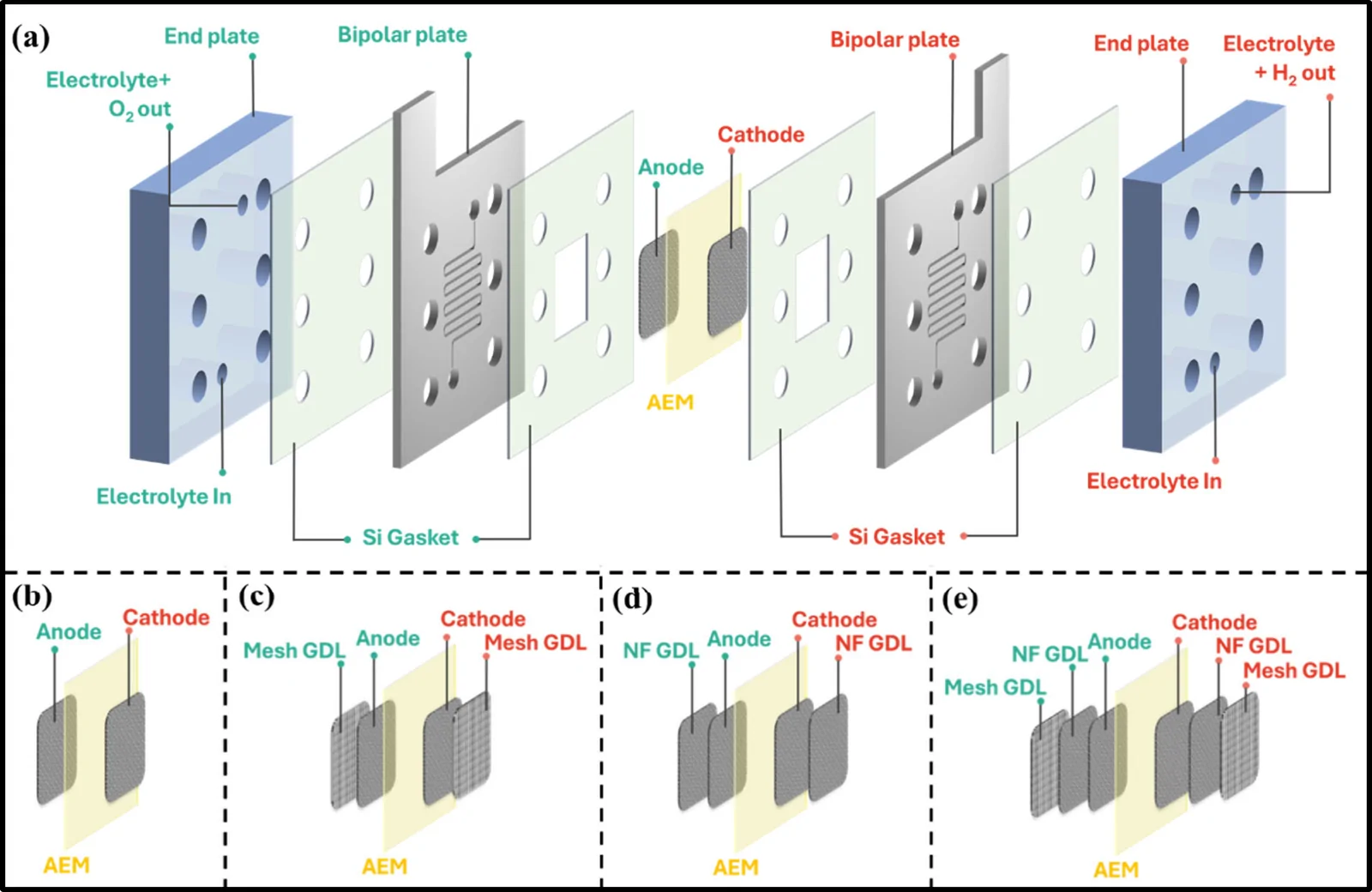
(a) Schematic of a zero-gap electrolyzer with its various components. (b–e) Schematics of membrane electrode assemblies with (b) NF, (c) NFM, (d) NFNF, and (e) NFNFM configuration.

Mass transport is a major issue in ZGAWE, especially at higher current densities.^88^ A gas diffusion layer (GDL) between the catalyst layer and the flow plates thus plays a major role in transferring reactants and products. It facilitates the removal of generated hydrogen and oxygen gases from the catalyst layer to prevent gas bubble accumulation, which can block active sites and reduce efficiency.^89,90^ Various GDLs have been explored, such as meshes, foams, 3D-printed electrodes, etc. Here, to highlight the impact of the GDL on the performance of ZGAWE, we have taken four configurations (Figure 11b–e): (1) NF as the catalyst without any additional GDL (written as NF); (2) NF as the catalyst with a stainless steel (SS) mesh as the GDL (i.e., NFM); (3) NF as the catalyst with additional NF as the GDL (i.e., NFNF); (4) NF as the catalyst with NF and SS mesh as GDLs (i.e., NFNFM).

The alkaline electrolyte 1 M KOH is circulated with a flow rate of 100 mL/min via a peristaltic pump for all measurements. Initially, the electrolyzer for all configurations is operated at 100 mA cm^–2^ for preconditioning to reach a steady state via the CP technique. Later, the LSV polarization curves are recorded at 2 mV/s, as displayed in Figure 12a. The sample NF shows fluctuations at higher current densities due to gas bubble accumulation on the surface. By incorporating SS mesh, the sample NFM shows less fluctuation in comparison to NF and also exhibits an enhancement in performance. Further, the NFNF and NFNFM sample shows almost negligible fluctuations due to the easy escape of gas bubbles and efficient mass transport. (Note: Never use NF as a GDL to test an electrocatalyst that is OER-active only. NF, being a good HER-active material, can participate in the reaction, and hence, the overall configuration can result in better overall activity, which is not the activity of the OER electrocatalyst alone. Try to use CC as a GDL for that case). The fitted EIS plot is presented in Figure 12b. The high-frequency resistance (HFR) is an important parameter that reflects the internal resistance of the cell, including ions and electronic resistance. The values of HFR obtained through the equivalent fitted circuits are presented in Table S7 for NF, NFM, NFNF, and NFNFM configurations. The cell with NF only shows high HFR in comparison to other samples, as shown in the inset Figure 12b, which signifies that the GDL can effectively reduce the internal resistance, thereby preventing ohmic losses and enhancing the cell efficiency.

**Figure 12:**
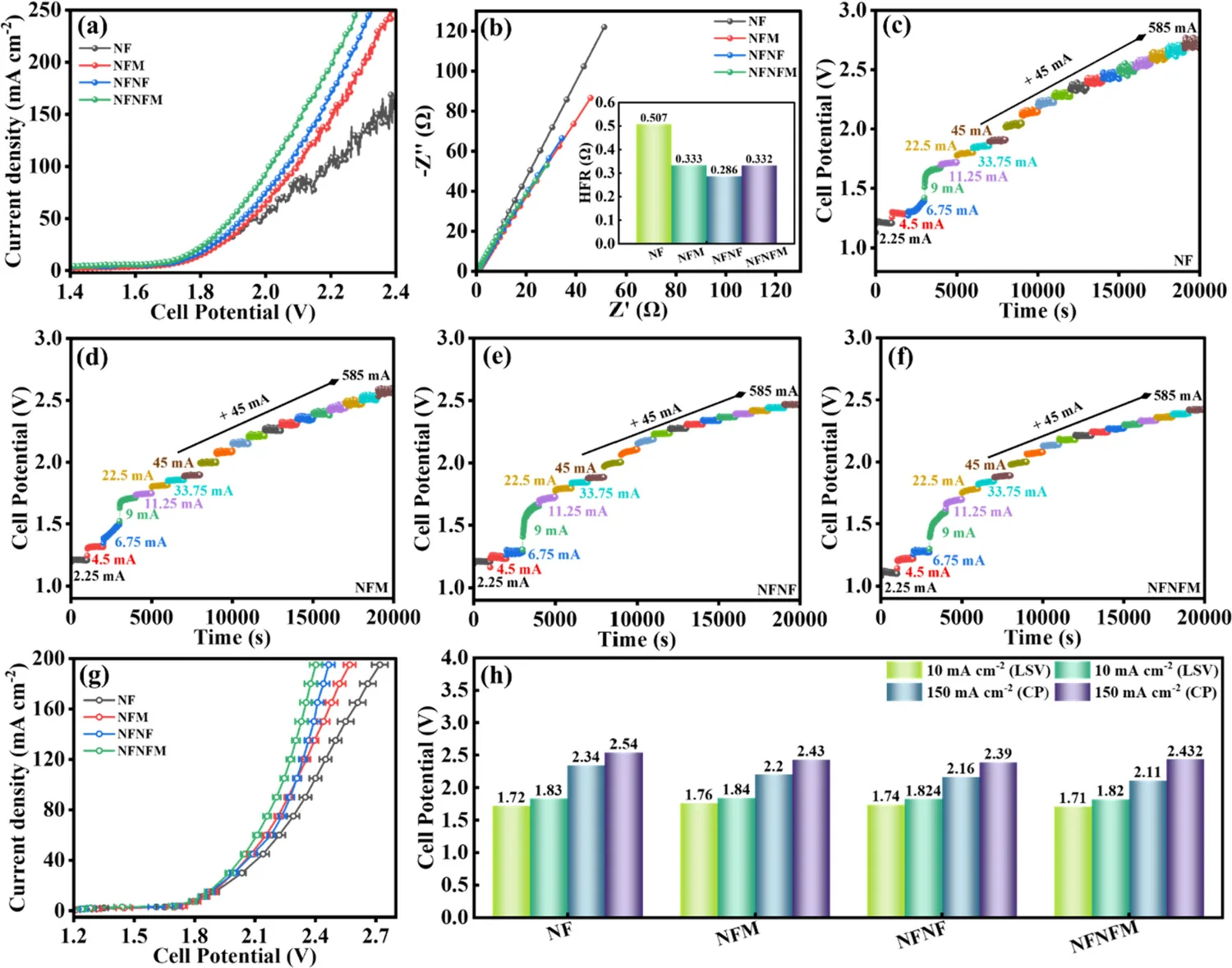
(a) LSV polarization curves. (b) Nyquist plots at 0 V for NF, NFM, NFNF, and NFNFM configurations. The inset shows corresponding HFR values. Step-CP measurements for (c) NF, (d) NFM, (e) NFNF, and (f) NFNFM configurations. (g) Polarization curves derived from step-CP measurements. (h) Differences in cell potential needed for 10 and 150 mA/cm^2^ current densities through LSV and CP measurements.

As discussed earlier, LSV polarizations are always accompanied by higher currents due to less time to equilibrate at a particular potential. Therefore, to mimic real-world electrolyzers, more reliable polarization curves should be extracted from steady-state measurements such as CP or CA. We have taken measurements by step CP, where the current density increases from 2.25 to 11.25 mA with a step of 2.25 mA, from 11.25 to 45 mA with a step of 11.25 mA, and from 45 to 585 mA with a step of 45 mA. The results are presented in Figure 12c–f for NF, NFM, NFNF, and NFNFM, respectively. The extracted polarization curves are shown in Figure 12g, which exhibit a similar trend to that obtained through LSV measurements, but with lower current densities at a particular potential. Furthermore, for clear comparison, the cell potentials required for the current densities of 10 and 150 mA cm^–2^, as measured by LSV and CP techniques, are presented in Figure 12h. It is observed that the cell potential needed through the LSV technique is always lower, i.e., > 5% lesser for lower current density (10 mA cm^–2^) and >15% lesser for higher current density (150 mA cm^–2^) for all NF, NFM, NFNF, and NFNFM samples. However, some recent studies still show that the polarization curves obtained through the LSV technique are only used for reporting the cell potential.^91–94^ But these measurements are not reliable for real-time electrolyzer operation, as shown above. Thus, one should extract polarization curves from a steady-state technique such as step-CP or CA to mimic the real response of the electrolyzer.

The stability of the electrocatalyst is another major concern that needs to be tested critically. However, many studies focus solely on electrocatalytic activity, often overlooking or inadequately examining stability. Testing procedures frequently employ lower current densities and shorter times than those required by industry, which do not accurately replicate actual industrial AWE settings. In order to create more resilient electrocatalysts, accelerated degradation tests that reliably replicate degradation processes over suitable time periods and realistic operating circumstances are required. Therefore, evaluating fluctuating currents is a valuable method for assessing the stability of catalysts.^95^ Here, we have tested two fluctuating currents, i.e., low (100 mA for 10 min) and high (800 mA for 10 min), for ∼30 cycles for the NFNFM configuration as presented in Figure 13a. The change in cell potential after the 30th cycle with respect to the initial cycle is displayed in Figure 13b, which shows an increment in cell potential of 100 mV due to degradation of the catalyst.

**Figure 13:**
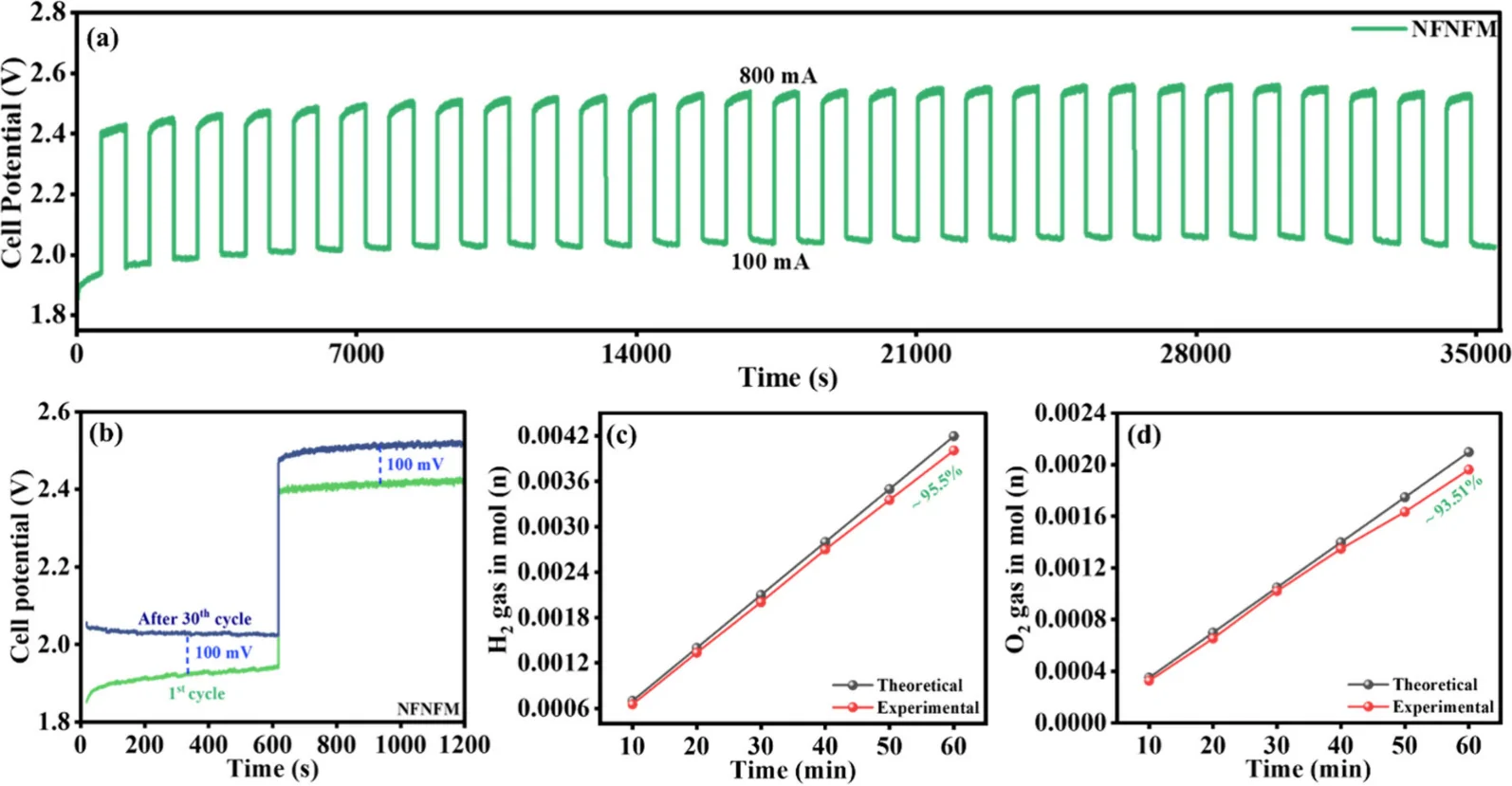
(a) Stability testing using fluctuating current for ∼30 cycles for NFNFM configuration, (b) change in cell potential before and after 30th cycle for NFNFM, number of moles calculated theoretically and experimentally for (c) H_2_ gas, and (d) O_2_ gas.

### 3.8 Faradaic Efficiency (FE)

In the above sections, we have discussed about activity and stability of an electrocatalyst. Besides these two parameters, selectivity is another important parameter for any electrocatalyst that can be described through FE. In electrochemical reactions, the applied current may contribute to both the target reaction products (i.e., H_2_ and O_2_) and various other parasitic side reactions i.e., catalyst reduction, corrosion, gas crossover, etc.^96,97^ FE quantifies the fraction of the total charge that is specifically used for the intended product formation. The word “Faraday” interrelates with Faraday’s constant (*F*), which correlates charge with moles of matter or substance as expressed in the laws of electrolysis given by Michael Faraday. The amount (moles) of product collected in relation to the amount that could be formed from the total charge passed, expressed as a fraction or a percent, is known as FE, and it characterizes the overall selectivity of an electrochemical process. Simply, it is the ratio of experimental number of moles of H_2_ or O_2_ gas (*n*_exptl_) to the theoretically calculated number of moles of H_2_/O_2_ gas (*n*_theor_), i.e.,

61 $$\mathrm{FE}=\frac{n_{\mathrm{exptl}}}{n_{\mathrm{theor}}}\times 100\%$$

It can be calculated using various methods such as gas chromatography, water–gas displacement method, and mass spectrometry method. Among all these techniques, the water–gas displacement method is one of the simplest methods and does not require advanced instrumentation.^98^ It requires a graded cylinder or buret filled with water and inverted in a container. One caution is to ensure the tube is completely filled, with no air bubbles. The evolved gas at a particular current or potential is then collected in the form of water displacement from the cylinder. The readings should be noted at different time intervals to calculate the total volume of gas collected (in mL) over a specific time period (i.e., 30–60 min), which can further be converted into moles of gas using Faraday’s second law of electrolysis.^99^ The theoretical number of moles of H_2_/O_2_ can be calculated using Faraday’s law, as follows:^100^

62 $$n_{\mathrm{theor}}=\frac{It}{zF}$$

where *I* is the current (in A), *t* is the time (in s), and *z* is the number of transferred electrons (e.g., *z* = 4 for O_2_ and *z* = 2 for H_2_), and *F* is the Faraday constant (96,485 C mol^–1^).

Experimentally, the number of moles of H_2_/O_2_ can be calculated using the ideal gas law (eq 63):

63 $$PV=n_{\mathrm{exptl}}RT$$

where *V* denotes the volume of collected gas, *T* denotes the temperature in kelvins, *R* denotes the ideal gas constant (0.0821 L·atm·mol^–1^·K^–1^), and *P* denotes the atmospheric pressure (∼1 atm).

Here we have used the water–gas displacement method to calculate the FE of the NFNFM configuration. The gases were collected at 225 mA current for a 60 min duration at each 10 min step. The measured volume of water displaced is then converted to moles of H_2_ and O_2_ as shown in Figure 13c,d, respectively. The calculated FE is ∼95.5% for H_2_ and ∼93.5% for O_2_ for a 60 min operation of the electrolyzer. This method is simple, yet it has several disadvantages, such as a portion of the generated gas dissolution in water leading to underestimation of FE, or the collected gas is saturated with water vapor, which expands the measured volume, leading to overestimation of FE. It cannot distinguish between H_2_, O_2_, or any other gaseous product.^101^ To overcome these issues, the most extensively used technique for determining the amount of evolved gas is presently gas chromatography (GC).^21^ It can be used to quantify FE even for low gas production rates, and also for a course of time when catalysts undergo surface modifications. It can also selectively quantify H_2_ and O_2_ using calibration curves.^102^ Yet, it also has several disadvantages, such as its high cost, requiring calibration with standard gases, and product collection is also required, which poses a risk of gas leakage. Standard GC is not inherently real-time unless coupled with an online sampling system. Recently, more advanced techniques that combine mass spectrometry (MS) with electrochemistry, such as differential electrochemical MS or electrochemical MS, have been used to monitor real-time FE.^103^ Its working requires a gas-permeable membrane that allows the transfer of gas only, whereas no electrolyte can pass through it. The gas then enters the MS under vacuum, which detects the mass-to-charge ratio of ions to quantify the gas.

### 3.9 Upscaling of Geometric Area of Electrocatalysts for Electrolyzers

The evaluation of electrocatalysts for water splitting is typically carried out at the laboratory scale using small electrode areas, often around 1 cm^2^, under well-controlled conditions. While such measurements provide valuable information on the intrinsic activity of catalytic materials, they do not accurately depict the performance characteristics observed in scaled-up systems. Scaling up the electrode area in water splitting electrolyzers from lab-scale (≤1 cm^2^) to larger practical dimensions (≫1 cm^2^) is a critical step toward real-world application of electrocatalysts. However, this transition is not merely a linear enlargement; it introduces several challenges that can significantly affect the overall catalytic performance. When transitioning to larger electrode areas, such as 10 cm^2^ or beyond, various physical, electrochemical, and mass transport phenomena begin to influence the apparent activity and overall efficiency of the catalyst system. Understanding how catalyst activity responds to increased electrode area is essential for bridging the gap between small-scale measurements and industrial-scale hydrogen production.

Here, we have presented the tested electrochemical results of scaling the geometric area of NF from 3 to 10 cm^2^ in Figure 14a. Ideally, the current density should be similar for both 3 and 10 cm^2^ areas, yet the reality is different. The electrode with the higher geometric area produces less current density than the electrode with a smaller area. Particularly, at 2.3 V, the observed difference in their current densities is ∼76 mA cm^–2^. This difference is due to the edge-to-bulk surface ratio, which is very high for smaller electrodes. Higher electric fields are frequently found at the electrode edges, which results in faster local reaction rates. The diffusion layer near the edges of the electrode varies notably from that at the center of the flat surface, leading to higher current densities at the edges.^104,105^ For electrodes larger than 1 cm^2^, the impact of edge effects becomes minimal, and the electrochemical reaction mainly takes place on the uniform surface, resulting in a more stable current density. Also, the diffusion of reactants (e.g., OH^–^) and the removal of gas bubbles (H_2_/O_2_) become more challenging at larger scales. Thus, bubble accumulation and slower diffusion may reduce the effective active surface area, increasing local overpotential. However, this activity trend may vary from catalyst to catalyst and becomes more challenging where uniform growth over the substrate is required.

**Figure 14:**
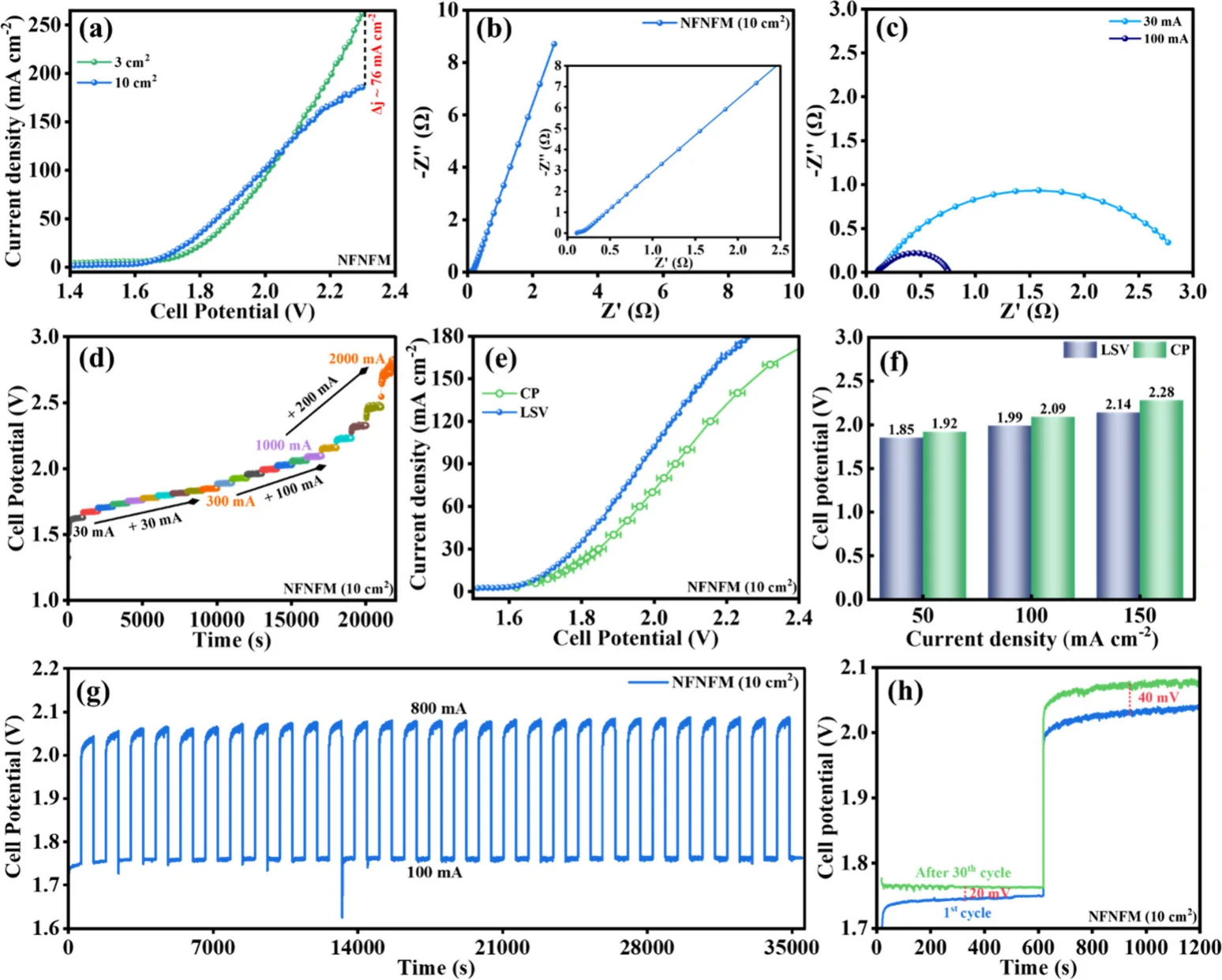
(a) LSV polarization spectra for 3 and 10 cm^2^ NFNFM. (b) Nyquist plot at 0 V. The inset shows a magnified view of the EIS plot. (c) Galvanostatic EIS for full Nyquist plot. (d) Step-CP measurements. (e) Polarization curves acquired through LSV and CP techniques. (f) Differences in cell potential needed for 10, 100, and 150 mA/cm^2^ current densities through LSV and CP measurements. (g) Stability testing using fluctuating current for ∼30 cycles. (h) Change in cell potential before and after the 30th cycle for NFNFM (10 cm^2^) configuration.

The EIS measurement at 0 V is used to determine the HFR of NFNFM for 10 cm^2^ (Figure 14b), which is nearly ∼0.110 Ω, as shown in the inset of Figure 14b. This decrement in HFR value from a 3 cm^2^ electrode is in good agreement with the inverse relationship of resistance with the area, as larger electrodes often provide more conductive pathways. Further, the full Nyquist plot is acquired using galvanostatic EIS at different currents of 30 and 100 mA, as presented in Figure 14c. The equivalent circuit used for fitting is presented in Table S8, and the obtained value of *R*_ct_ is 2.79 Ω for 30 mA and 0.561 Ω for 100 mA, representing easy charge transfer through the electrode–electrolyte interface. The more reliable polarization curve can be derived from step CP/CA measurements as mentioned earlier. Here, the step CP measurements are taken where the current density increases from 30 to 300 mA with a step of 30 mA, from 300 to 1000 mA with a step of 100 mA, and from 1000 to 2000 mA with a step of 200 mA, as shown in Figure 14d. The comparison of the polarization curve taken from LSV and derived through CP measurements is presented in Figure 14e. The difference in activity obtained is quite high and should not be neglected. The observed difference in cell potential accounts for ∼70 mV (for 10 mA cm^–2^), ∼100 mV (for 100 mA cm^–2^), and ∼140 mV (for 150 mA cm^–2^) higher cell potentials in steady-state measurements, Figure 14f.

Further, the acceleration degradation tests were also performed to gather information about the stability of higher area electrodes. We have tested two similar fluctuating currents, i.e., low (100 mA for 10 min) and high (800 mA for 10 min), for ∼30 cycles for the NFNFM (10 cm^2^) configuration as presented in Figure 14g. The change in cell potential after the 30th cycle with respect to the initial cycle is displayed in Figure 14h, which shows an increment of 20 mV for 100 mA and 40 mV for 800 mA current. The observed increment in both currents after 30th cycle is less in comparison to that observed in 3 cm^2^ electrode area, which is in good agreement of larger electrodes provide more stable current.

### 3.10 AWE under Industrial Conditions

The measurement protocols described in this tutorial are primarily demonstrated at room temperature in 1 M KOH, which remains the most widely adopted laboratory benchmark for catalyst evaluation. However, practical AWE typically operates under substantially different conditions, namely 60–80 °C in concentrated KOH electrolytes (6–7 M, ∼25–30 wt %).^106,107^ Elevated temperature accelerates the kinetics of both HER and OER by lowering activation barriers and increasing exchange current densities, thereby reducing the overpotential required to achieve a given current density.^108,109^ Simultaneously, concentrated alkaline electrolytes exhibit significantly higher ionic conductivity, leading to lower uncompensated resistance (*R*) and consequently smaller *iR* losses.^110^ However, the key challenges include managing bubble-induced ohmic resistance at high current densities, ensuring long-term durability (>60,000 h) of electrodes, and maintaining gas purity (>99.5% H_2_) under dynamic renewable power input.^52,108^ Elevated temperature also alters gas evolution dynamics by decreasing electrolyte viscosity and surface tension, promoting faster bubble detachment, reducing bubble coverage, and improving mass transport; however, operating at industrial current densities (>0.5–2 A cm^–2^) can still result in significant bubble-induced transport limitations.^112–114^ Furthermore, kinetic parameters such as the apparent Tafel slope may differ from those measured under ambient conditions because the dominant rate-determining step, reactant activity, surface coverage, and mass transport characteristics change with temperature and electrolyte concentration.^107,115,116^ Therefore, when translating laboratory protocols to industrially relevant testing, electrochemical impedance spectroscopy (EIS)-based resistance measurements, dynamic *iR* compensation, temperature-controlled electrochemical cells, and high-current-density polarization measurements should be performed under the intended operating conditions. These considerations are essential for obtaining performance metrics that accurately reflect commercial alkaline electrolyzer operation and for narrowing the gap between laboratory catalyst evaluation and practical device performance.

## 4 Conclusions

In the pursuit of efficient and sustainable hydrogen production through electrochemical water splitting, the accurate assessment of electrocatalyst performance is both crucial and challenging. This tutorial has comprehensively outlined the best measurement practices for evaluating the key performance parameters that define catalyst efficiency, activity, selectivity, and durability. A deep understanding of performance parameters is essential for accurate interpretation and comparison across various electrocatalytic systems. The LSV/CV measurements are largely affected by scan rates; in order to get a more reliable overpotential, one should never exceed a 2 mV/s scan rate. Also, *iR* compensation in LSV/CV curve is done with proper care and should be compared with the system-compensated and manual *iR* drop to report justified current densities at lower overpotentials. For an electrocatalyst exhibiting a redox peak, a reverse scan of the LSV should be performed to compare its performance with that of other reported catalysts. The EIS measurements should be done at catalytic turnover conditions to get a more precise value of charge transfer resistance and should be done in a proper frequency range of at least 10^5^ to 1 Hz or 10^5^ to 0.1 Hz with a small AC amplitude.

All examined electrocatalysts in electrocatalytic water splitting are first screened using a transient polarization approach, such as cyclic voltammetry (CV) or linear sweep voltammetry (LSV), at a constant potential ramping rate. These methods do not give the catalytic interface enough time to settle at a specific potential in order to produce steady-state responses. As a result, the response from a steady-state method like chronoamperometry (CA) is always lower than the observed current density for a given overpotential in LSV/CV. This increased current density is due to double-layer charging or the catalyst’s self-electrooxidation, hence CA/CP measurements are more reliable to get Tafel slopes and ultimately charge transfer resistance. The non-Faradaic regions should be properly screened in order to get a double-layer capacitance free from capacitance contributed from redox-peak or any other side reactions. For Ni-based materials, adsorbate capacitance can also be calculated for a more precise value of ECSA. The ECSA-normalized LSV can be used to isolate the specific activities of the catalyst from the higher surface area effect.

The TOF is an important intrinsic parameter that is not reported in several articles, but it should be reported properly, not only at a single overpotential value but also in the range of overpotential values. In contrast to the overpotential recorded at a benchmarking current density, which essentially acts as an apparent activity marker, TOF provides us the precise data on the rate at which a fuel is used or the amount of the desired fuel produced in an energy conversion process. The redox peak integration method can be used to calculate the TOF of transition metal-based catalysts. Stability is one of the important parameters for any catalyst’s industrial use. Here, we have highlighted several ambiguities while using CP measurements to demonstrate the stability. Instead, CA measurements should be used to report a more accurate and fairer comparison of stability with other reported electrocatalysts. A schematic for the systematic methodology to be followed during three-electrode measurements is presented in Figure 15.

**Figure 15:**
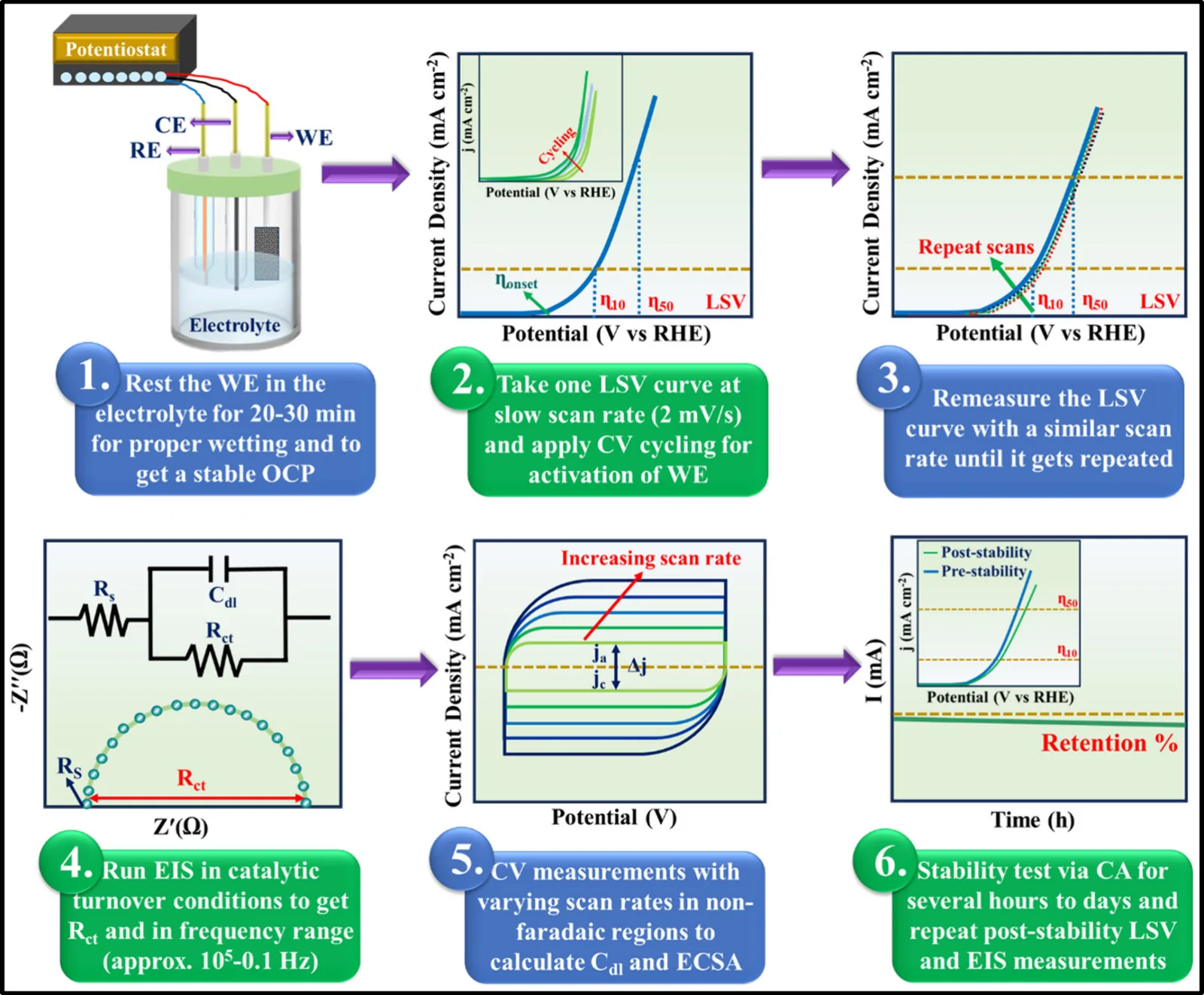
Schematic of the systematic approach of techniques used to extract performance parameters in a three-electrode system.

Emphasis is also laid on transitioning from three-electrode testing to a complete cell, i.e., alkaline water electrolyzer. Various aspects of effective membrane electrode assembly are discussed in detail. The components of the electrolyzer can affect its performance, primarily through the tuning of the gas diffusion layer with the electrocatalyst. The polarization curves derived from step-CP/CA measurements are more reliable instead of LSV techniques. Electrocatalyst selectivity can be assessed by measuring faradaic efficiency, which quantifies the yield of H_2_/O_2_ gas produced. Apart from catalytic activity, stability remains one of the most critical challenges for practical electrolyzers. Conventional lab-scale durability tests often fail to replicate industrial operating conditions. To bridge this gap, it is essential to evaluate electrode stability under fluctuating currents (i.e., high and low) to better mimic real-world operation. A schematic for the systematic methodology to be followed during water electrolyzer measurements is presented in Figure 16. Upscaling of the electrode geometric area is another major concern to bridge the gap between lab-scale and real-world electrolyzers. Larger areas may experience uneven potential and current densities, resulting in nonuniform reaction rates. At larger scales, diffusion paths for reactants and products increase, often causing concentration gradients, bubble accumulation, and reduced efficiency. Maintaining catalyst integrity and strong adhesion to the substrate over large electrode surfaces is more challenging, which we, as researchers, are trying to mitigate. Thus, we believe this tutorial will guide researchers in reporting error-free electrocatalyst performance metrics and also help beginners in the field learn the best measurement practices for evaluating electrochemical water splitting performance parameters.

**Figure 16:**
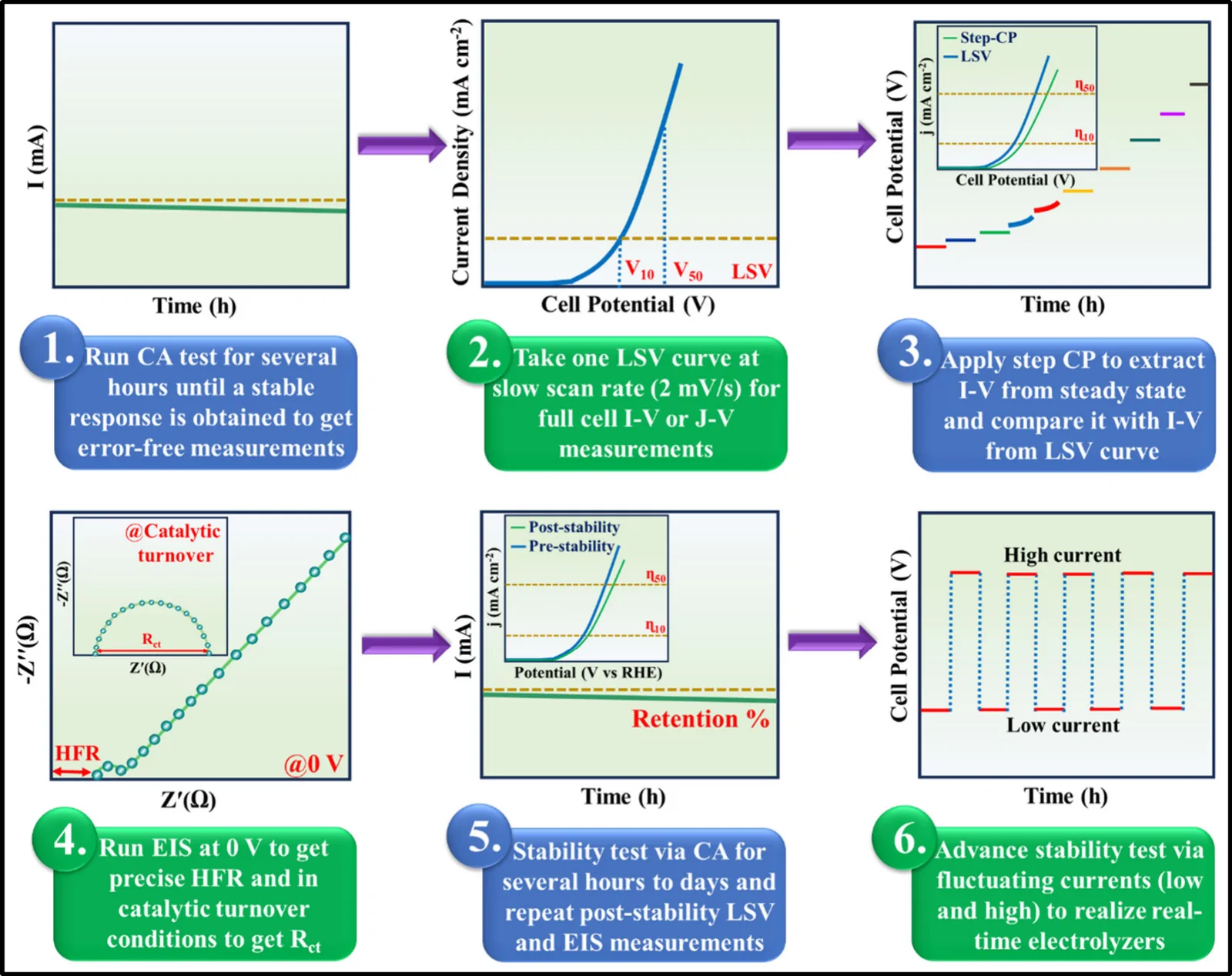
Schematic of the systematic approach of techniques used to extract some performance parameters in a water electrolyzer.

## Supplementary Material



## Notes

Materials and reagents, AWE setup details, *iR* compensation image from AUTOLAB, synthesis of NiS@NF, and equivalent circuits along with fitting element details for all EIS plots used in this Tutorial (PDF)
