# Supplementary material for: A Tutorial on Measurement of Electrochemical Water-Splitting Performance Parameters: Methods and Best Practices
Source: ACS Meas Sci Au. 2026 Aug 21;6(5):1445–70. doi: 10.1021/acsmeasuresciau.6c00147 (PMC13693225; doi:10.1021/acsmeasuresciau.6c00147)
Supplement: Supplementary file 1 [file tg6c00147_si_001.pdf]

Supporting Information

A Tutorial on Measurement of Electrochemical Water-Splitting Performance Parameters: Methods and Best Practices

Minakshi Sharma[1], Chandra Prakash[1], Vijay K. Singh[3]*, Ambesh Dixit[1,2]*

[1]Advanced Materials and Device (A-MAD) Laboratory, Department of Physics, Indian Institute of Technology Jodhpur, Rajasthan-342030, India

[2]Rishabh Centre for Research and Innovation in Clean Energy, Indian Institute of Technology Jodhpur, Jodhpur, Rajasthan-342030, India

[3]Department of Physics, Indian Institute of Technology Ropar, Rupnagar, Punjab-140001, India

*Corresponding authors: ambesh@iitj.ac.in, vksingh@iitrpr.ac.in

Materials and Reagents

Sulphuric acid (H_2SO_4) and Hydrochloric acid (HCL, 37 wt.%) (Sigma Aldrich), Carbon cloth (CC, thickness ~ 0.3 mm, carbon fibre content ~ 95%) and Nickel Foam (NF, thickness ~ 1.5 mm, Purity ~ 99.9%) (Nano Global Technologies Pvt. Ltd.), Stainless-Steel mesh (SS, thickness ~ 0.15 mm) and Si gaskets (thickness ~ 1 mm) from METNMAT Innovations Pvt. Ltd., India, 2-propanol (Sigma Aldrich), Sulphur powder (Thermo scientific) and Potassium hydroxide (KOH) pellets (Rankem).

Cleaning of carbon cloth and nickel foam

The as-received CC was pretreated to make its surface hydrophilic. Firstly, in 0.5 M H_2SO_4 for 3-4 h, then in DI water for 30 min using ultrasonication, and finally kept for drying at 60 °C in an electrical oven. The NF was sonicated in 0.5 M HCl to remove any oxide layer, if present, followed by subsequent sonication in 2-propanol, ethanol, and deionised water for 10 min each. Afterwards, nitrogen gas was used to blow away the water and finally kept for drying at 60 °C in an electrical oven.

Anion-Exchange membrane (AEM) assisted alkaline water electrolyser (AWE) set up:

The AEM-assisted alkaline water electrolyzer (AWE) used in this study was purchased from METNMAT Innovations Pvt. Ltd., India. The 1 M KOH electrolyte was continuously circulated through the cell using a peristaltic pump (RAVEL HITEKS Pvt. Ltd., Model RH-

P120S) operated at 50 rpm, corresponding to a flow rate of approximately 55 mL min^{-1}, which was maintained throughout all experiments. Sealed borosilicate glass bottles served as electrolyte reservoirs for both the catholyte and anolyte (1 M KOH) and for gas collection. The front and end plates are made of transparent PMMA. Ni-coated SS-316 stainless steel plates and SS-316 served as both the electrolyte flow-field plates and the current collectors for the anode and cathode, respectively. The flow plates have a single serpentine flow pattern design. A Fumasep FAS-50 (thickness: 45-55 µm, conductivity: 3-8 mS cm^{-1}, ion-exchange capacity: 1.6-2.0 mmol g^{-1}) anion-exchange membrane purchased from Fuel Cell Store was employed as the separator after pretreatment in 1 M KOH for 24 h. To facilitate efficient reactant transport and gas release, various gas diffusion layers (GDLs) were incorporated into the cell as described in the manuscript.

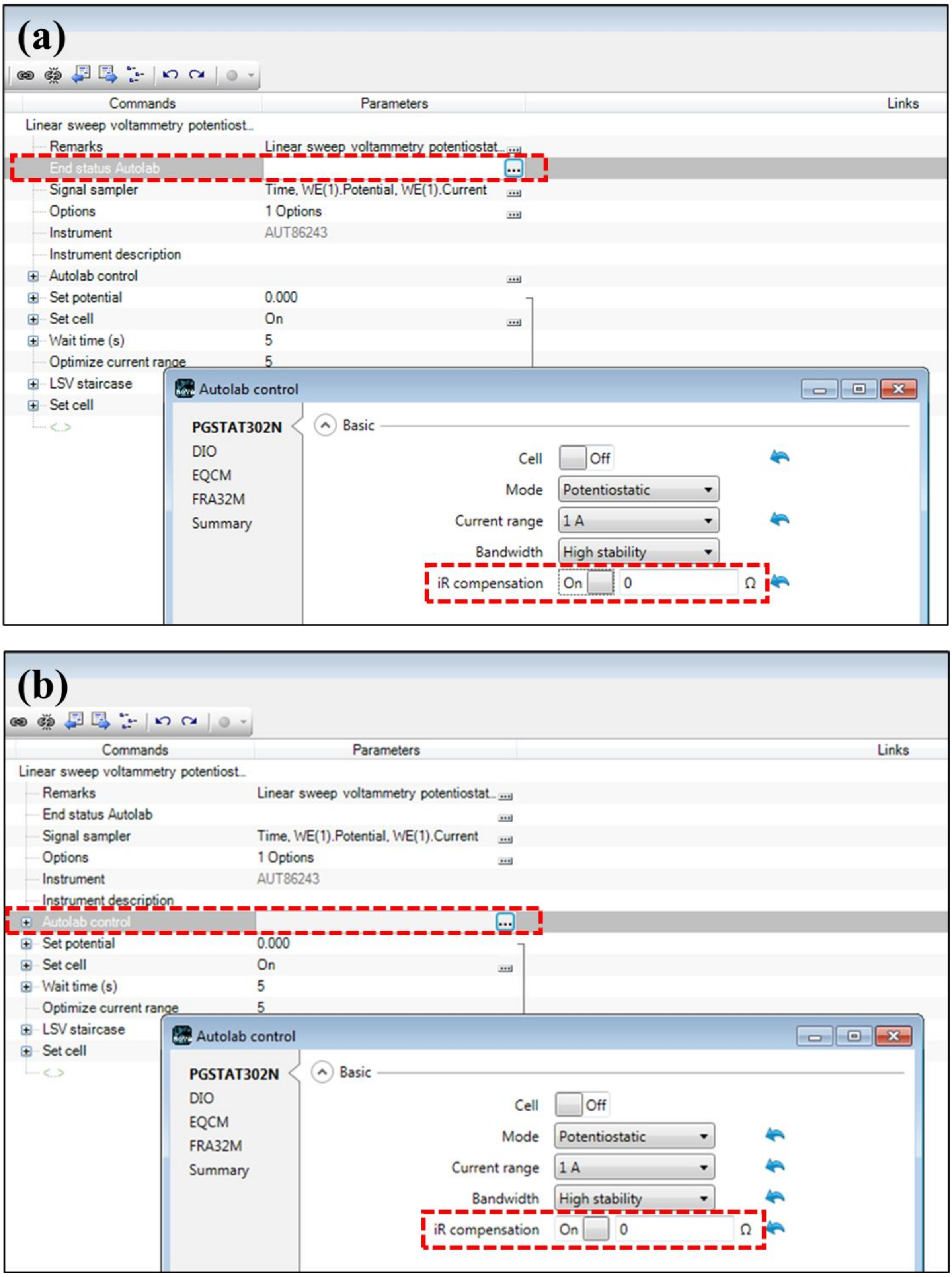

Figure S1: iR compensation using Autolab PGSTAT302N **(a)** using End Status Autolab option, **(b)** Autolab control option.

Synthesis of NiS@NF:

500 mg sulphur powder and three pieces of NF (2*1 cm^2) were kept facing vertically in a stand and were placed in the Ist and IInd zones of CVD, respectively. The quartz tube diameter used for deposition was 45 cm. The carrier gas Ar+H_2 (100 sccm) was used for deposition. The temperature of the Ist zone was set at 300 °C, and for the IInd zone at 500 °C, and kept at these temperatures for 1 h for deposition. Afterwards, the samples were allowed to naturally cool with the continuous flow of Ar+H_2. The mass loading was found to be ~15 mg cm^{-2}.

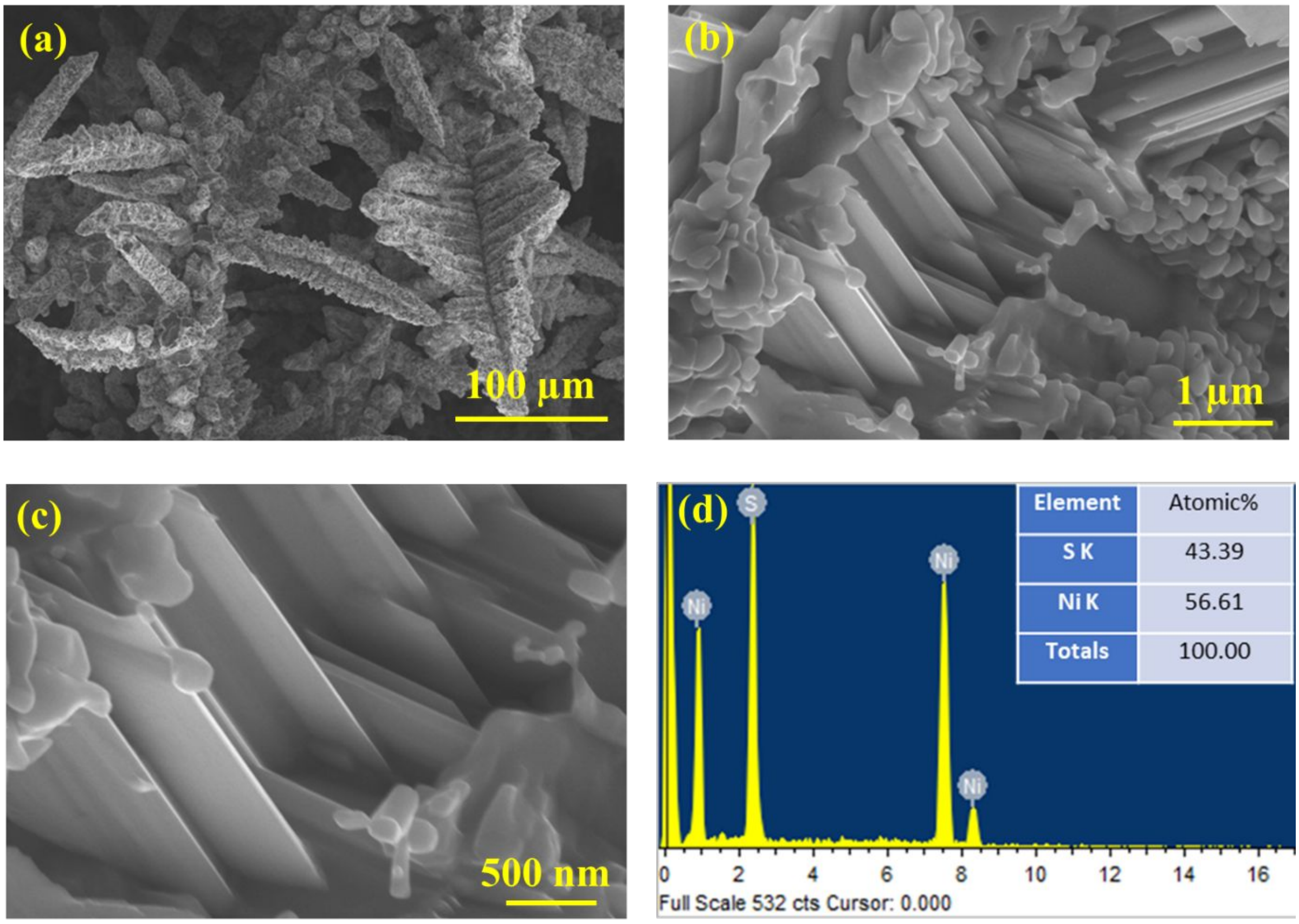

Figure S2: (a-c) FESEM micrographs of NiS@NF at various magnifications, **(d)** EDX spectrum of NiS@NF, inset table showing elemental composition.

Table S1: Equivalent circuit for CC with varying potentials at a frequency of 10^5-1 Hz with a 5 mV ac wave amplitude.

Potential (V)	Equivalent circuit
1.82	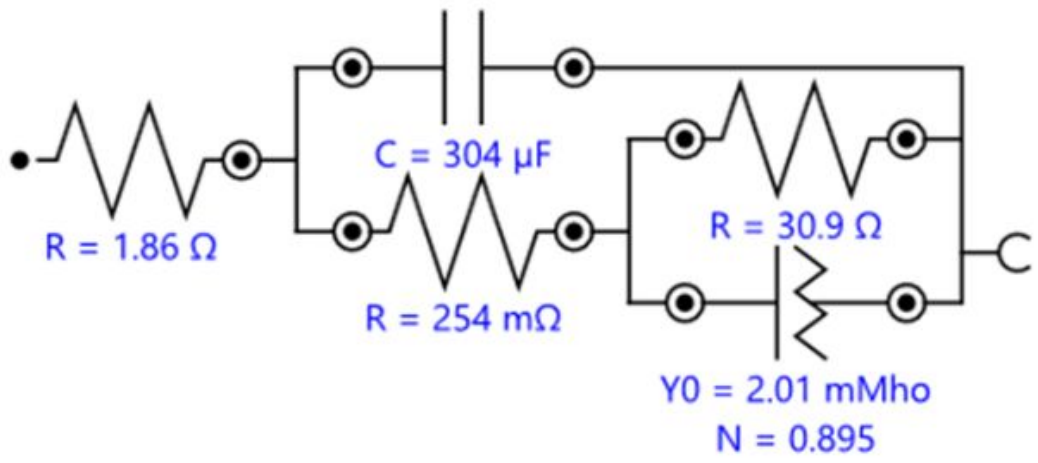
1.84	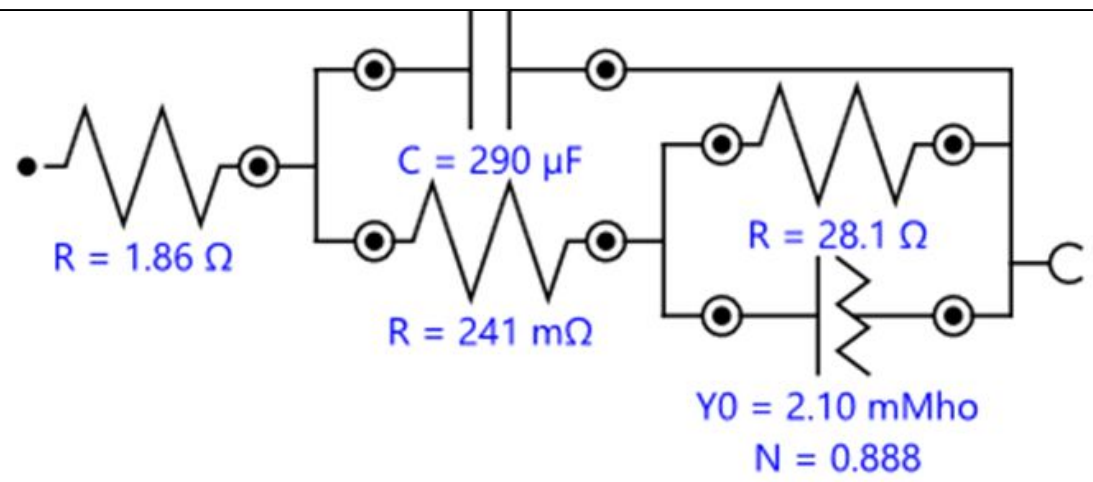
1.87	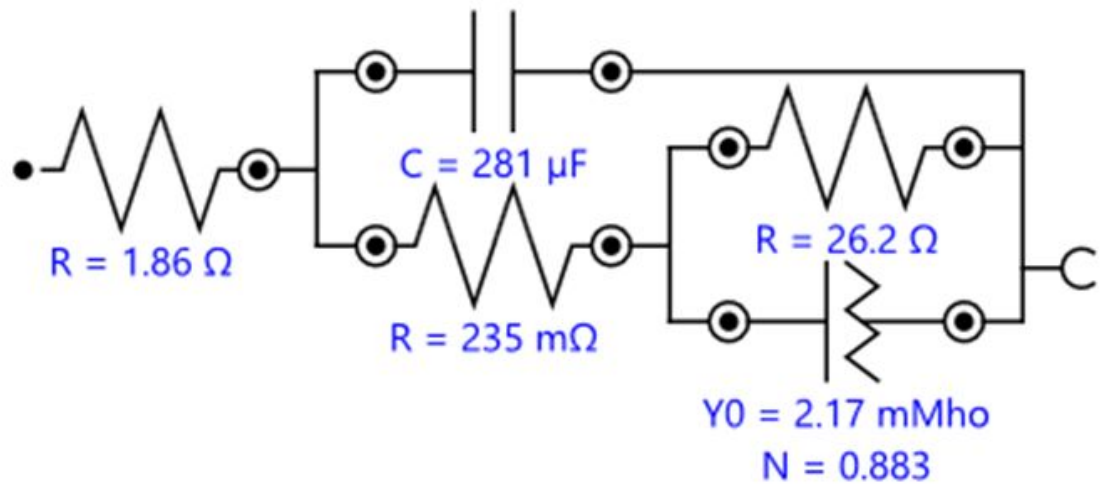
1.90	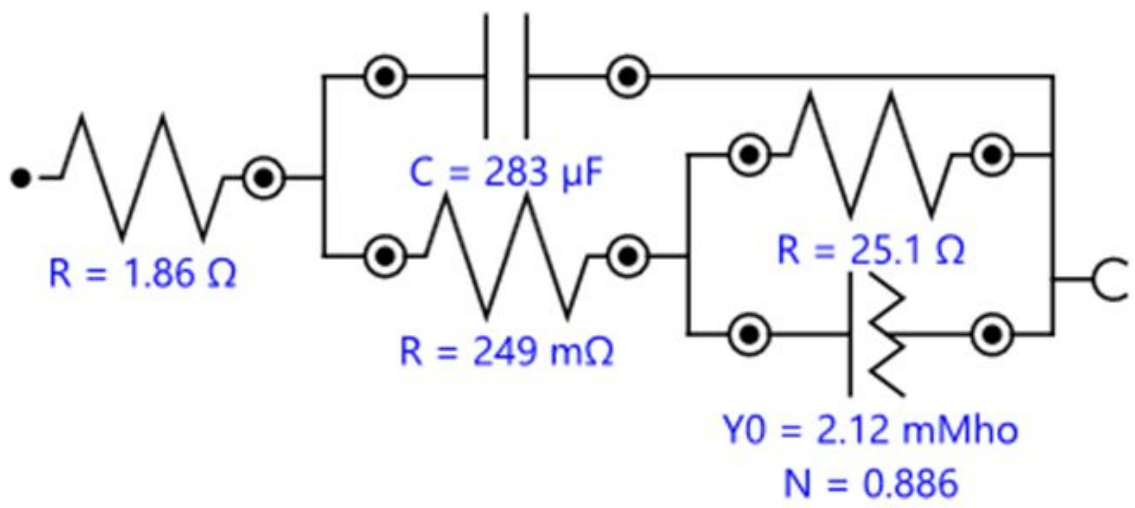
1.92	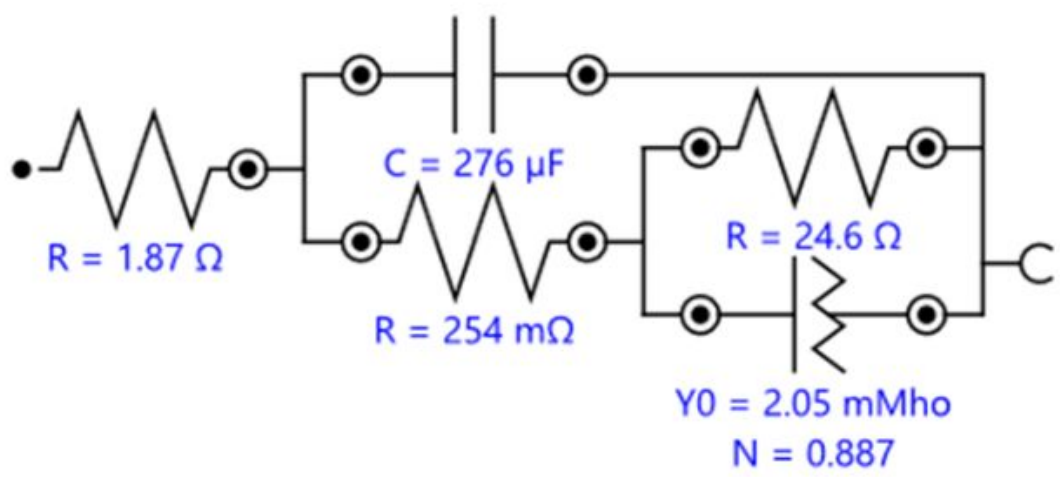

<table>
<tr><td>1.94</td><td>
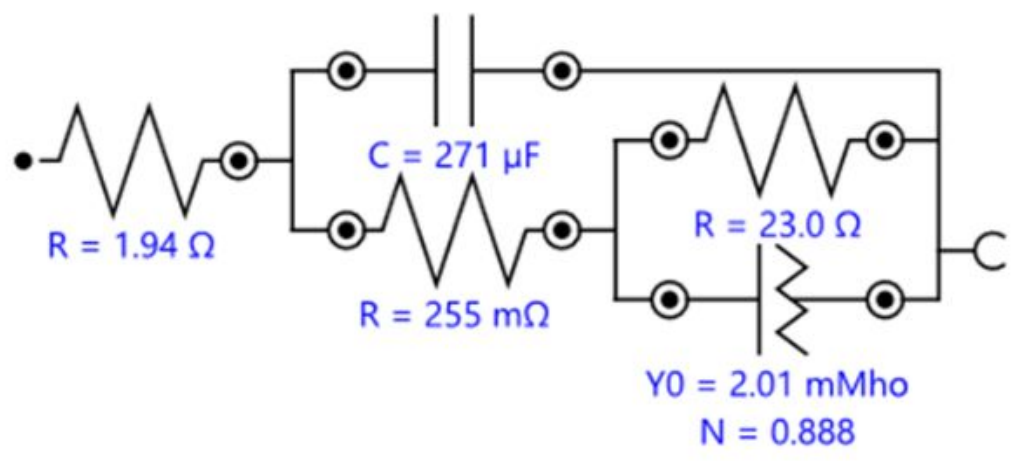

</td></tr>
<tr><td>1.97</td><td>
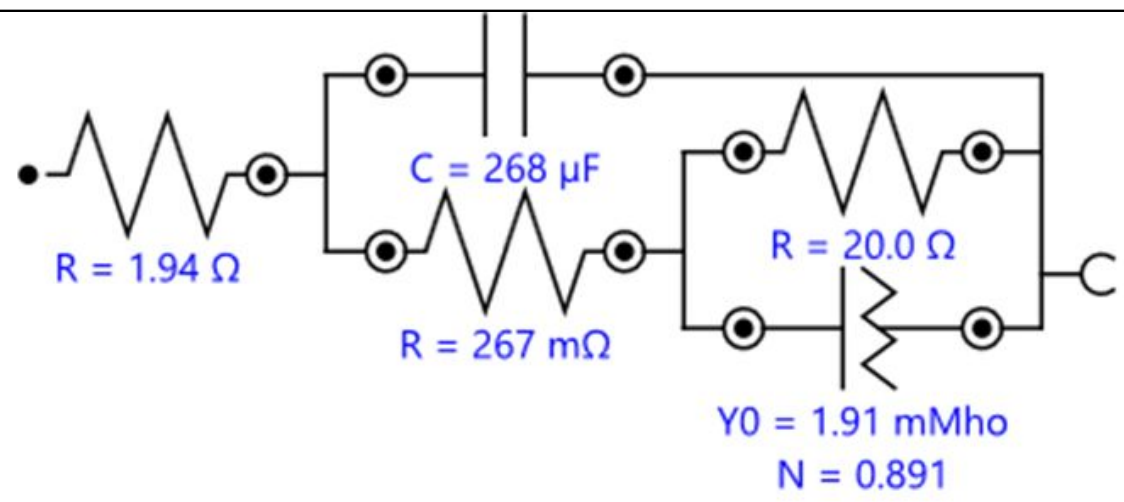

</td></tr>
<tr><td>2.00</td><td>
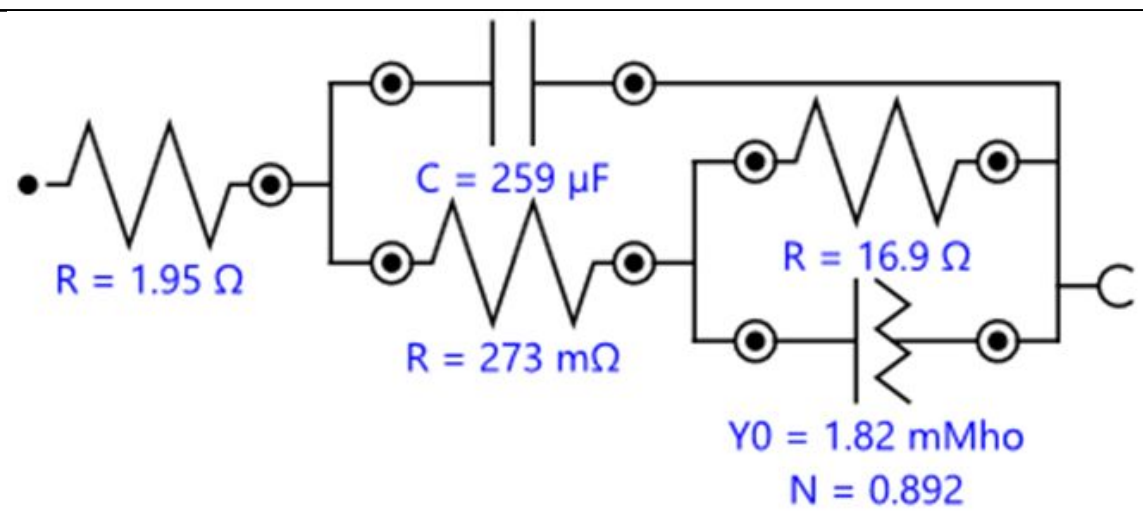

</td></tr>
<tr><td>2.02</td><td>
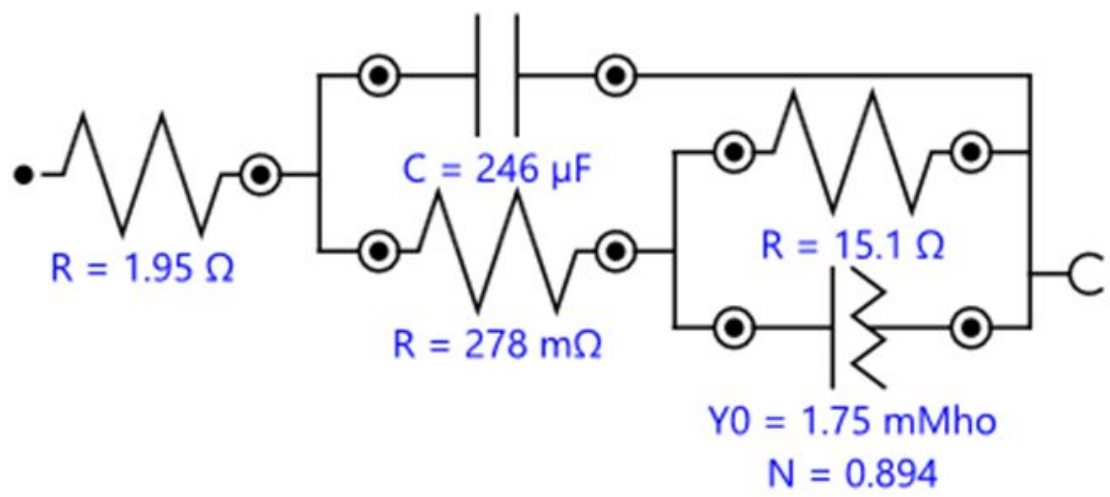

</td></tr>
<tr><td>2.04</td><td>
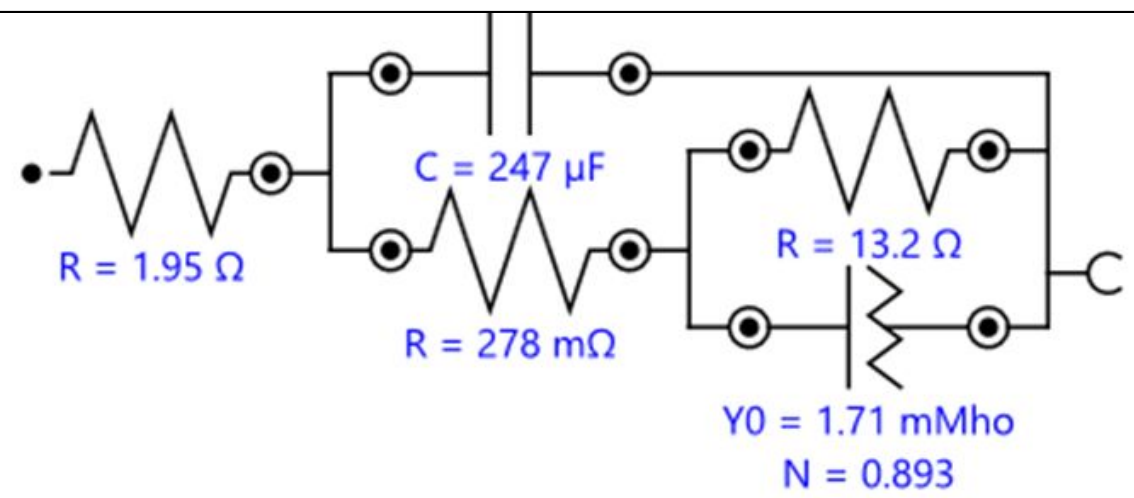

</td></tr>
<tr><td>2.07</td><td>
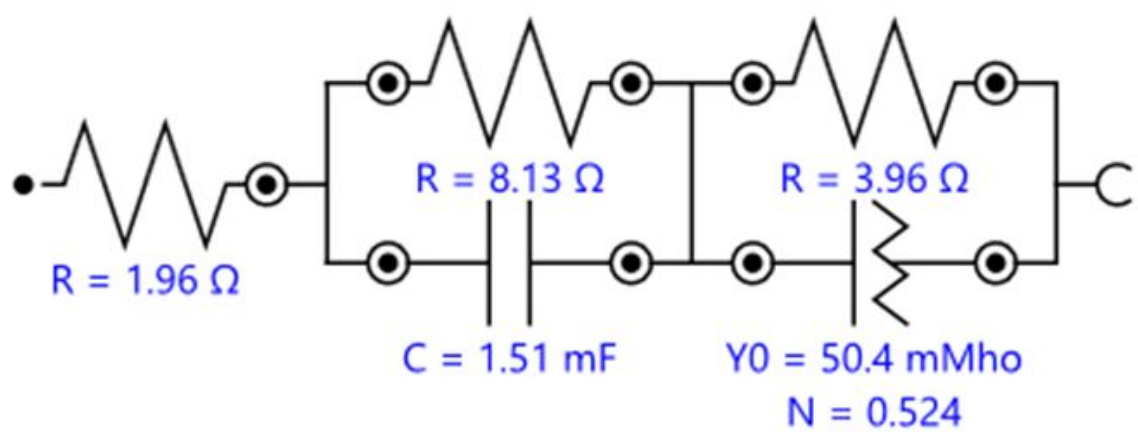

</td></tr>
</table>

2.10	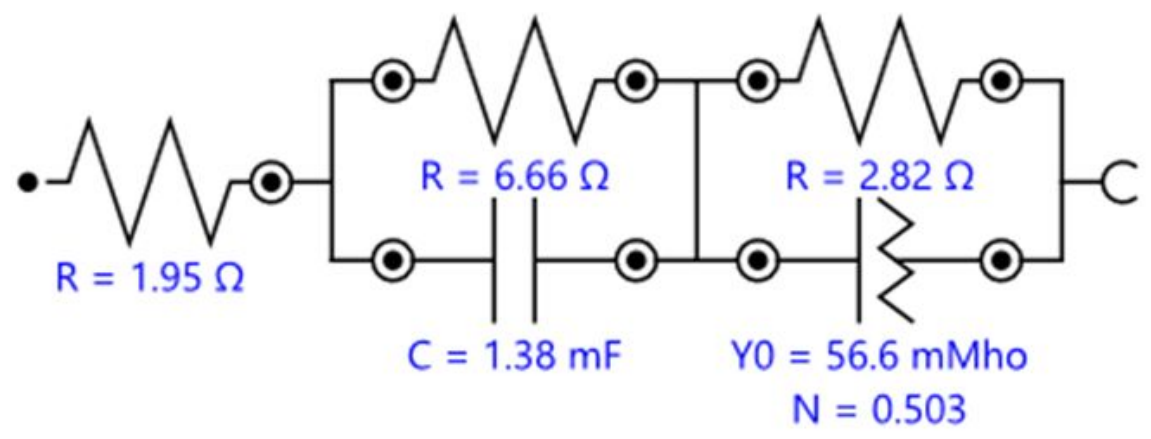<br>R = 6.66 Ω<br>R = 2.82 Ω<br>R = 1.95 Ω<br>C = 1.38 mF<br>Y0 = 56.6 mMho<br>N = 0.503
2.12	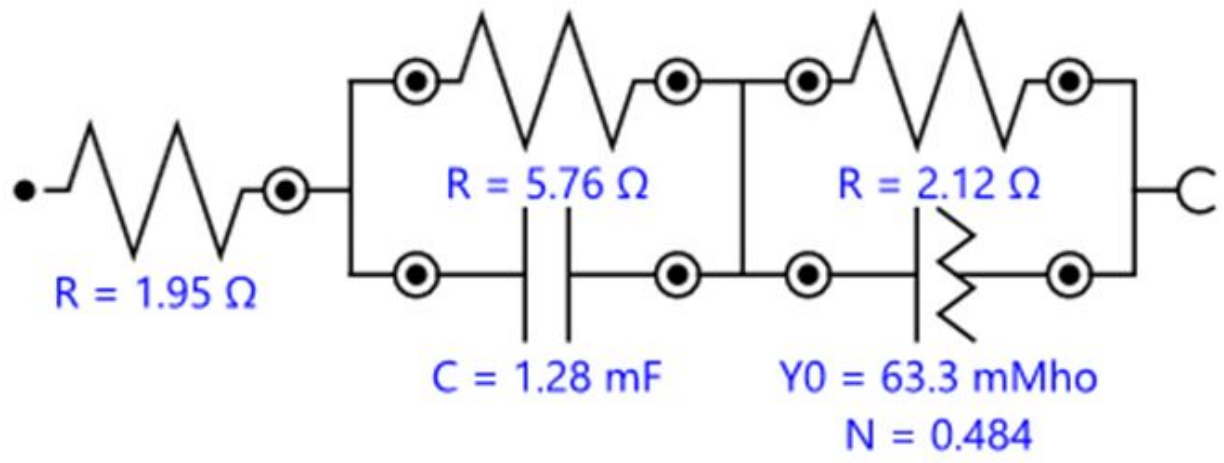<br>R = 5.76 Ω<br>R = 2.12 Ω<br>R = 1.95 Ω<br>C = 1.28 mF<br>Y0 = 63.3 mMho<br>N = 0.484

Table S2: Equivalent circuit for CC at 2.07 V with varying lower frequency range and higher frequency kept fixed (10^5 Hz) with a 5 mV ac wave amplitude.

Frequency (Hz)	Equivalent circuit
100	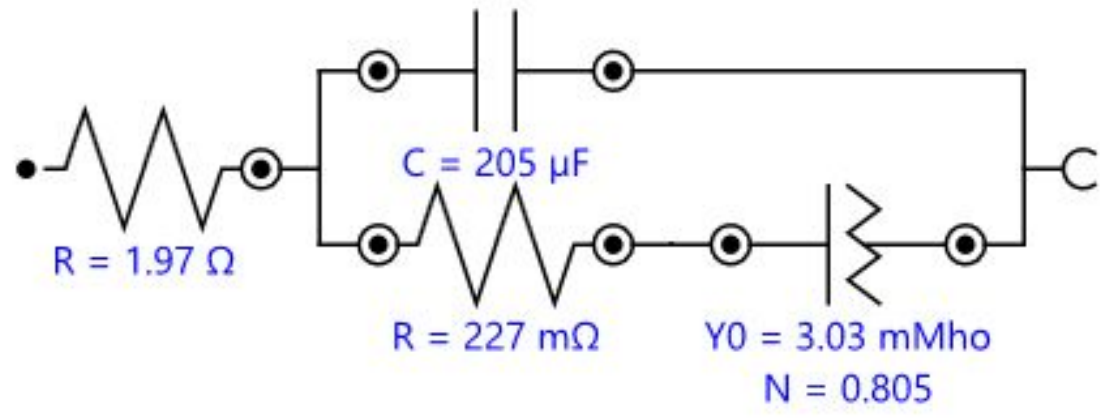
10	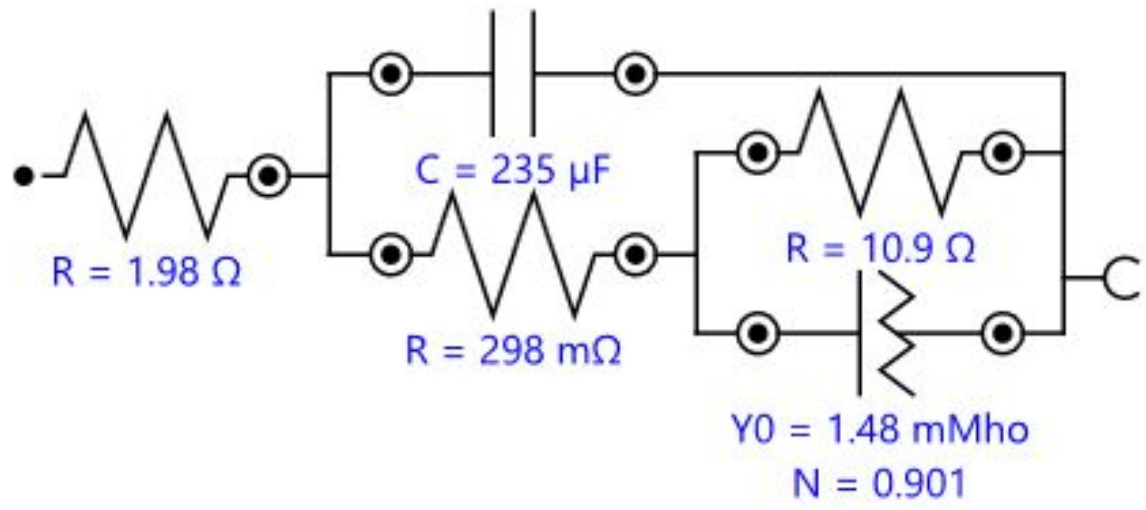
1	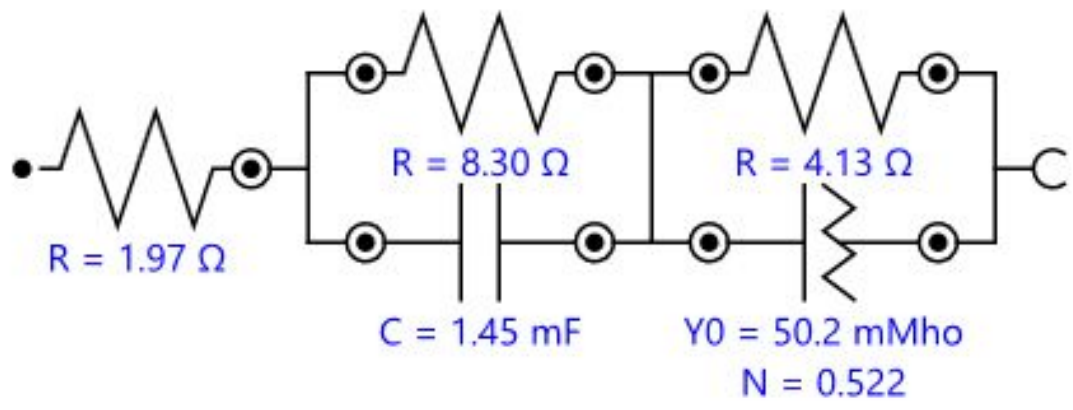
0.1	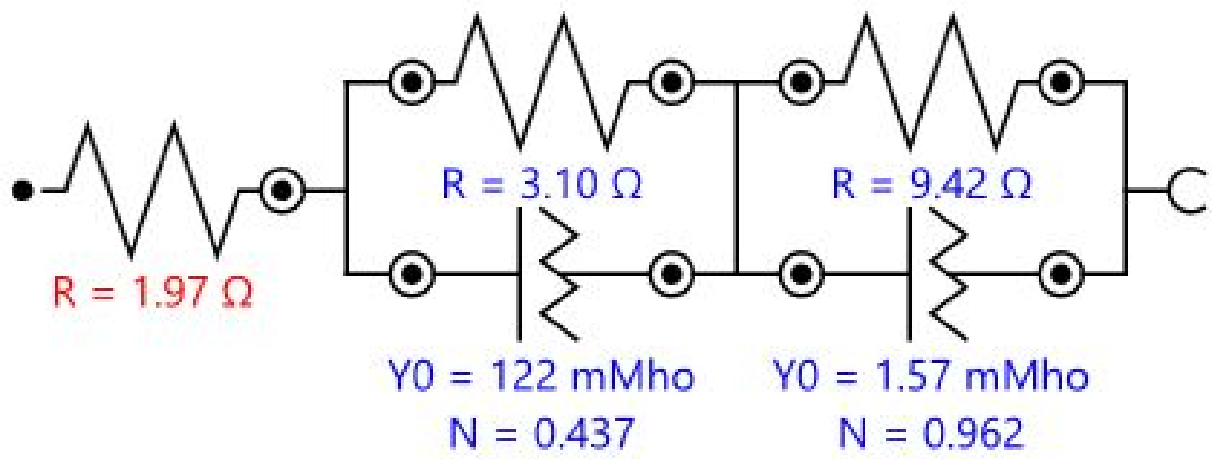
0.01	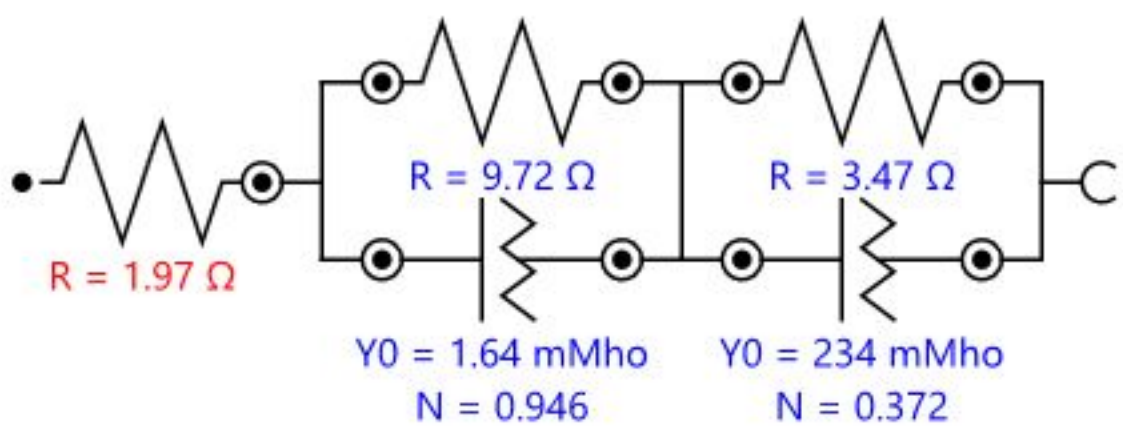

Table S3: Equivalent circuit for CC at 2.07 V with varying amplitude of ac wave at a frequency range of 10^5-1 Hz.

<table>
<tr><th>Amplitude (mV)</th><th>Equivalent circuit</th></tr>
<tr><td>1</td><td>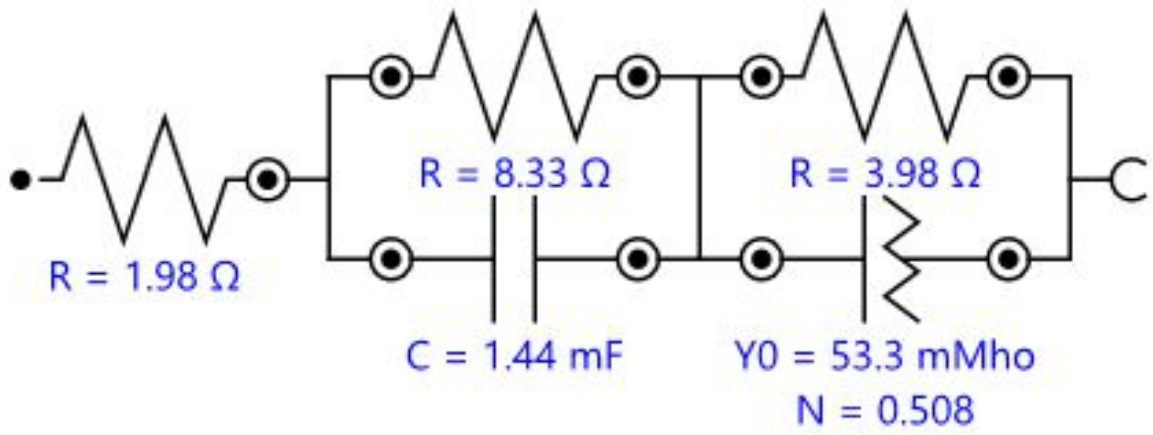
</td></tr>
<tr><td>2</td><td>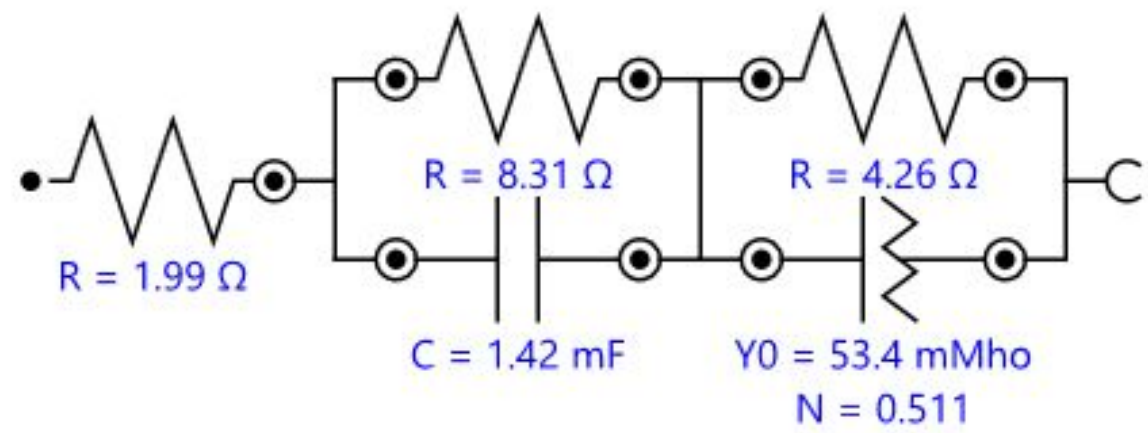
</td></tr>
<tr><td>5</td><td>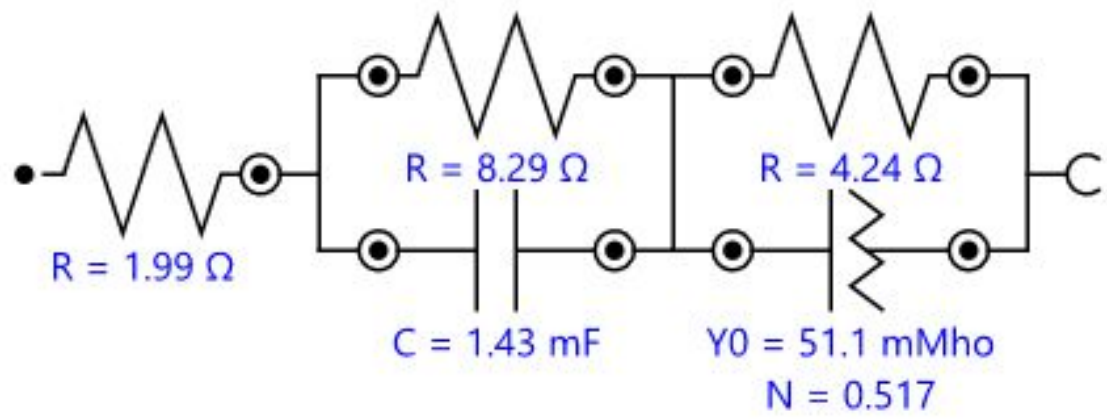
</td></tr>
<tr><td>10</td><td>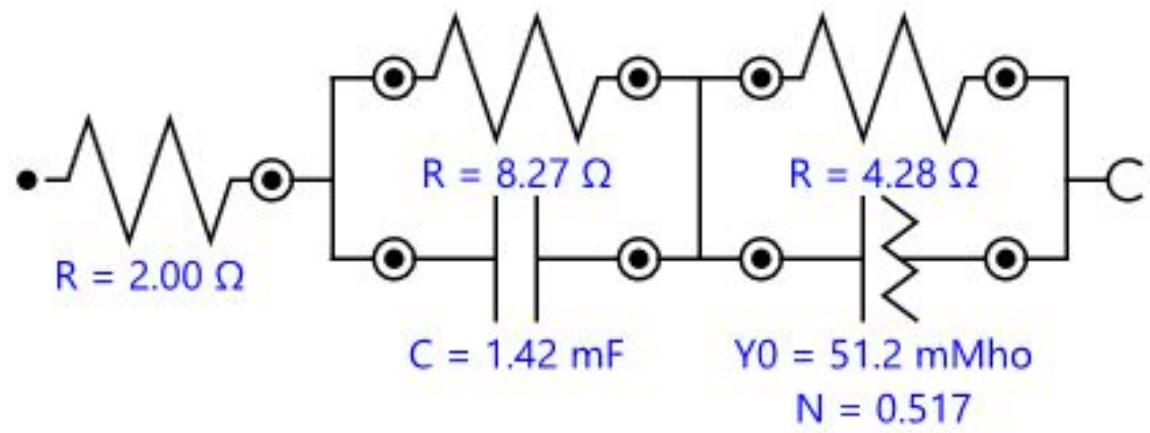
</td></tr>
</table>

Table S4: Equivalent circuit for NF with varying potentials at a frequency of 10^5-1 Hz with a 5 mV ac wave amplitude.

Potential (V)	Equivalent circuit
1.50	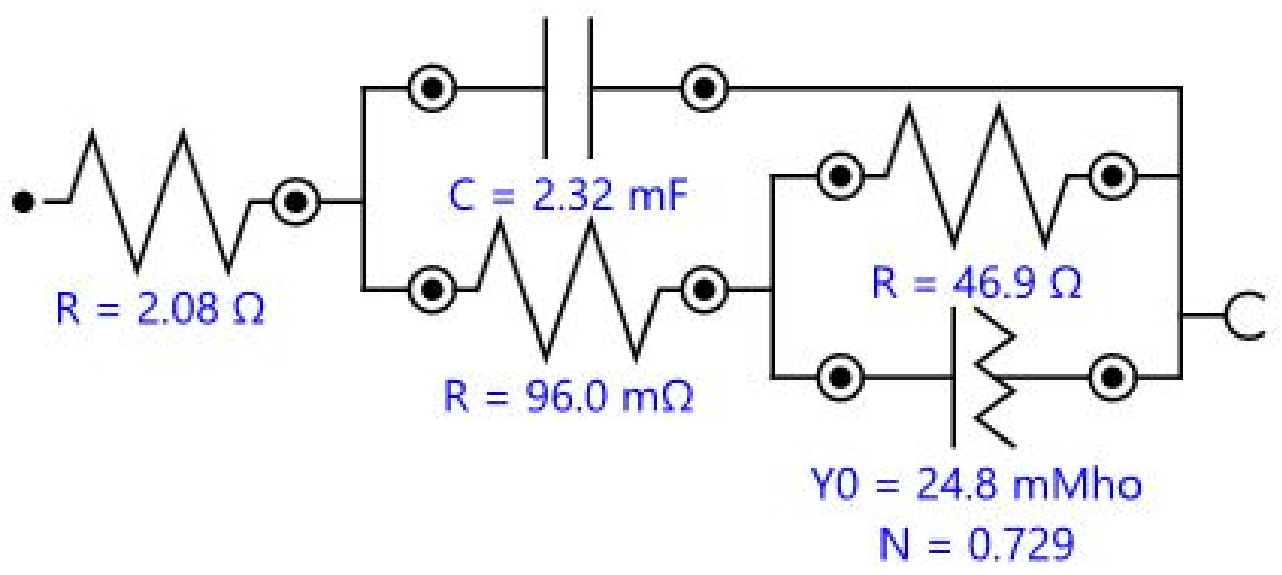
1.52	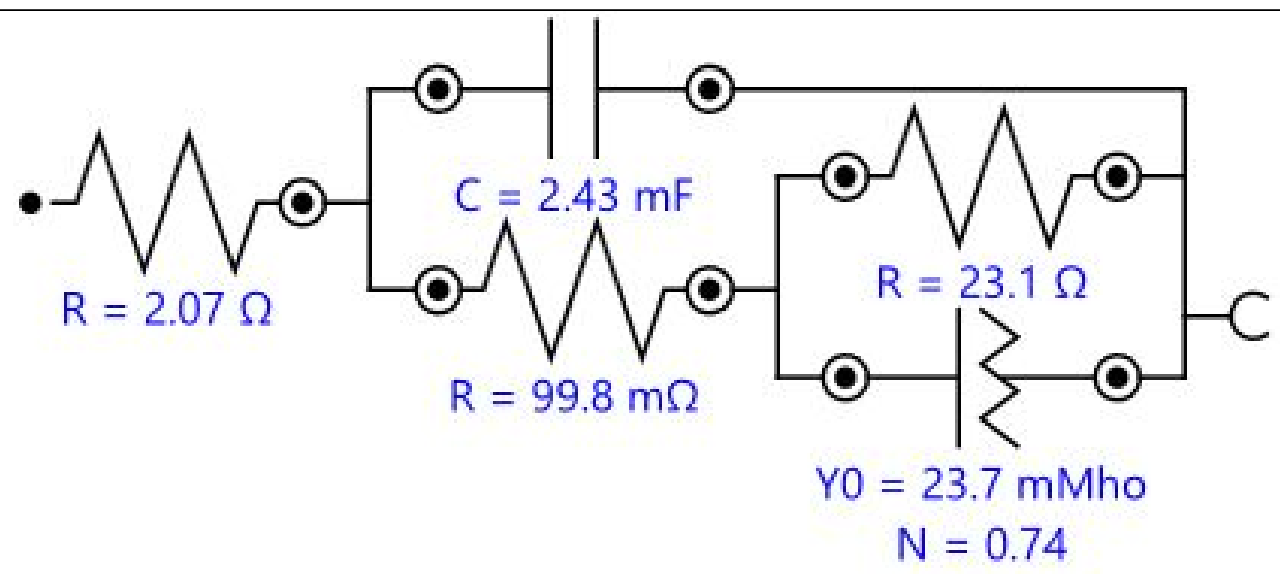
1.54	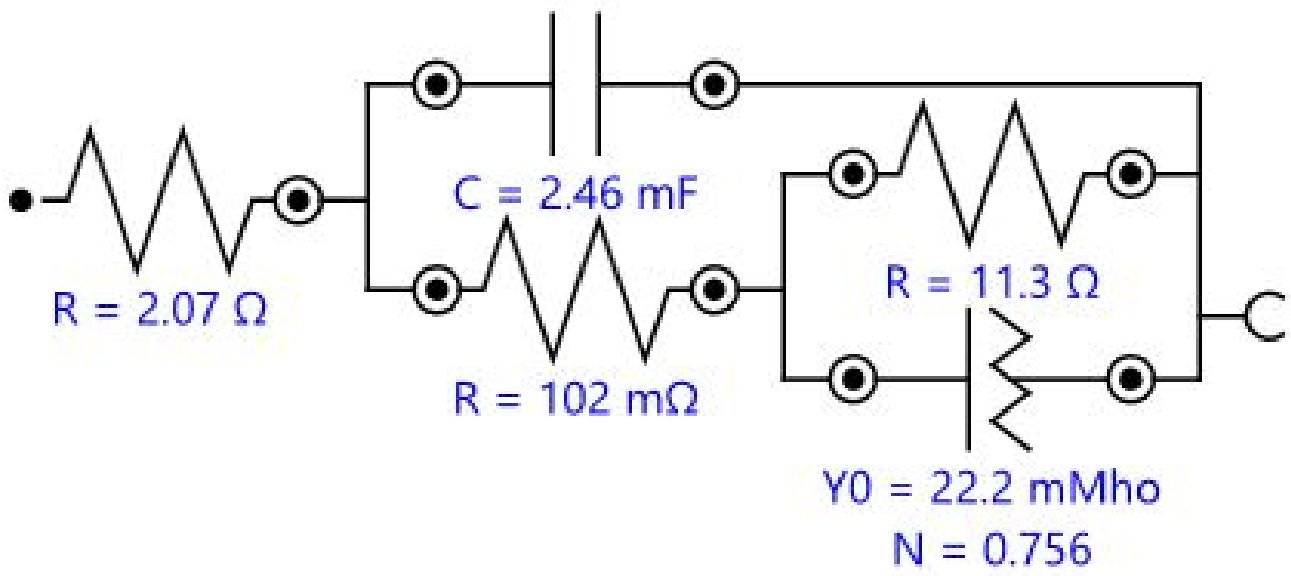
1.57	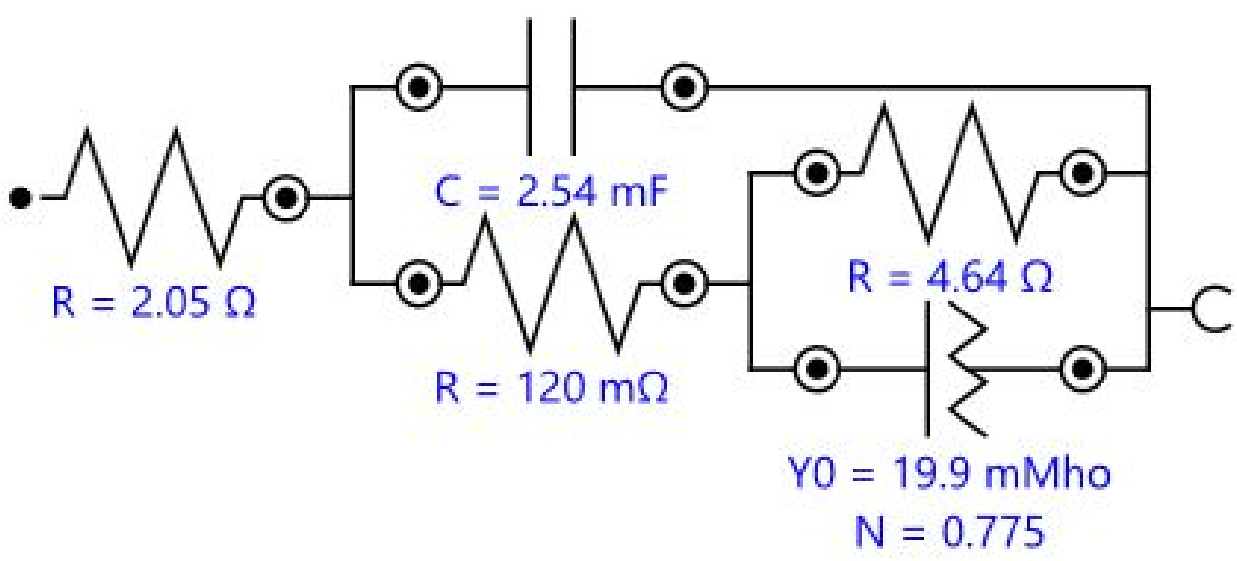
1.60	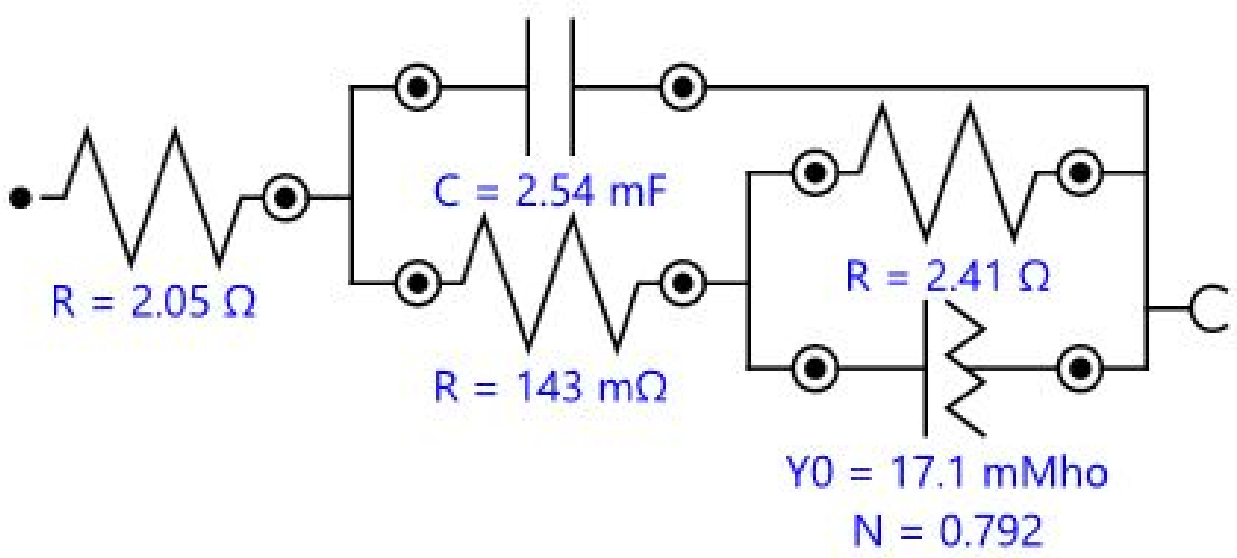

1.62	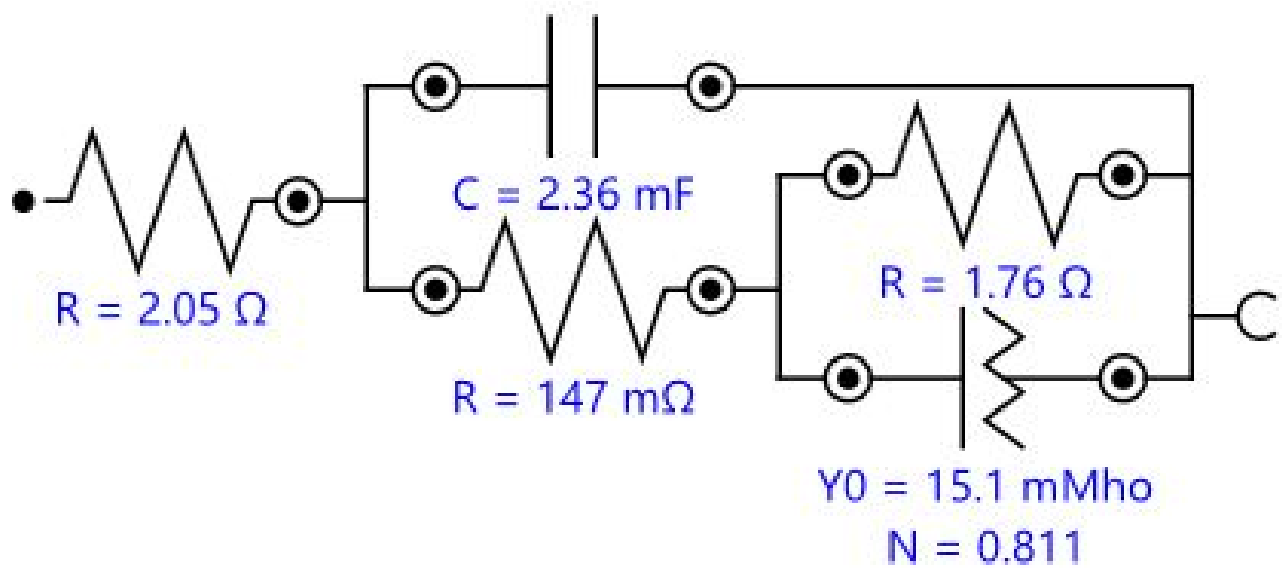
1.64	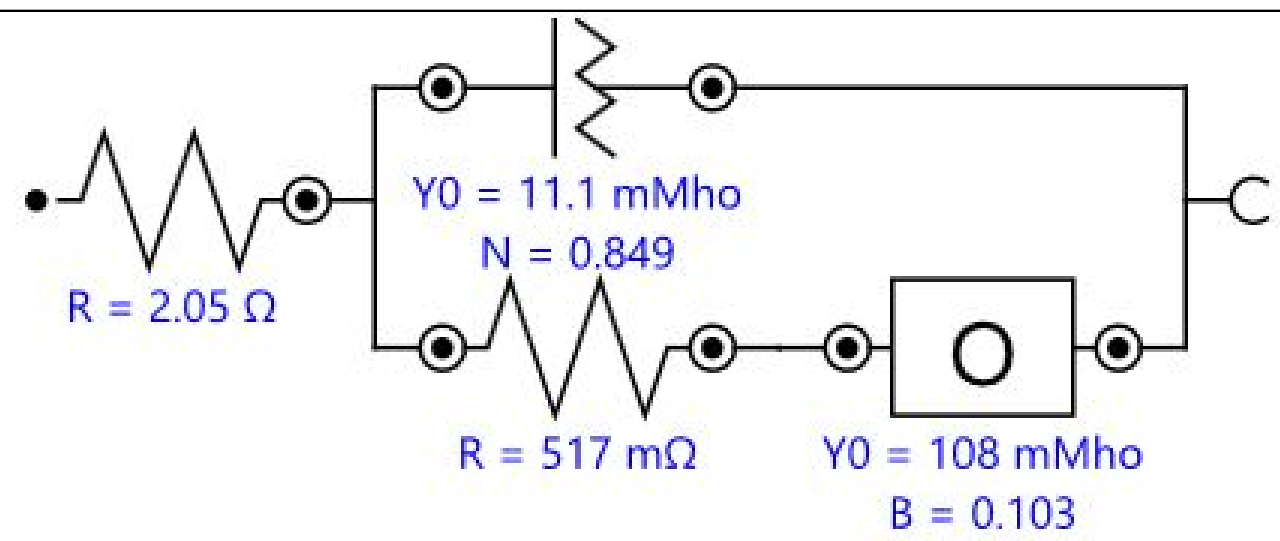
1.67	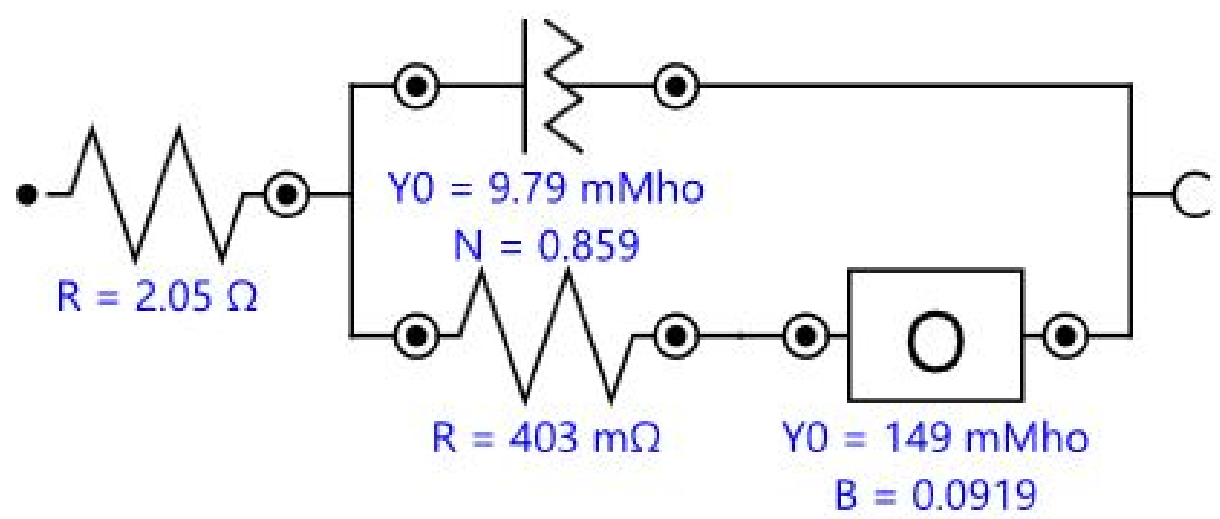
1.70	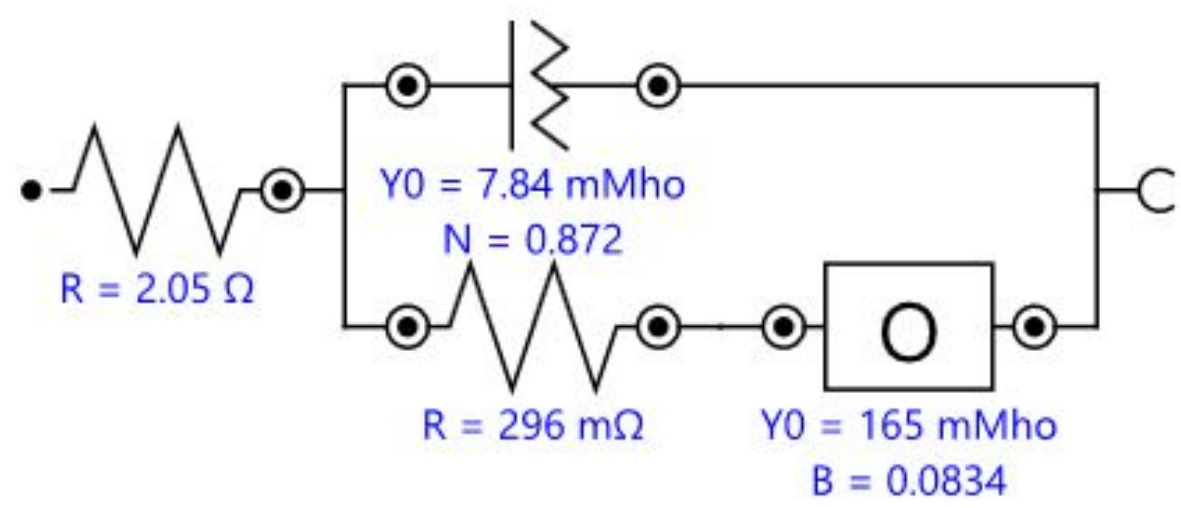
1.72	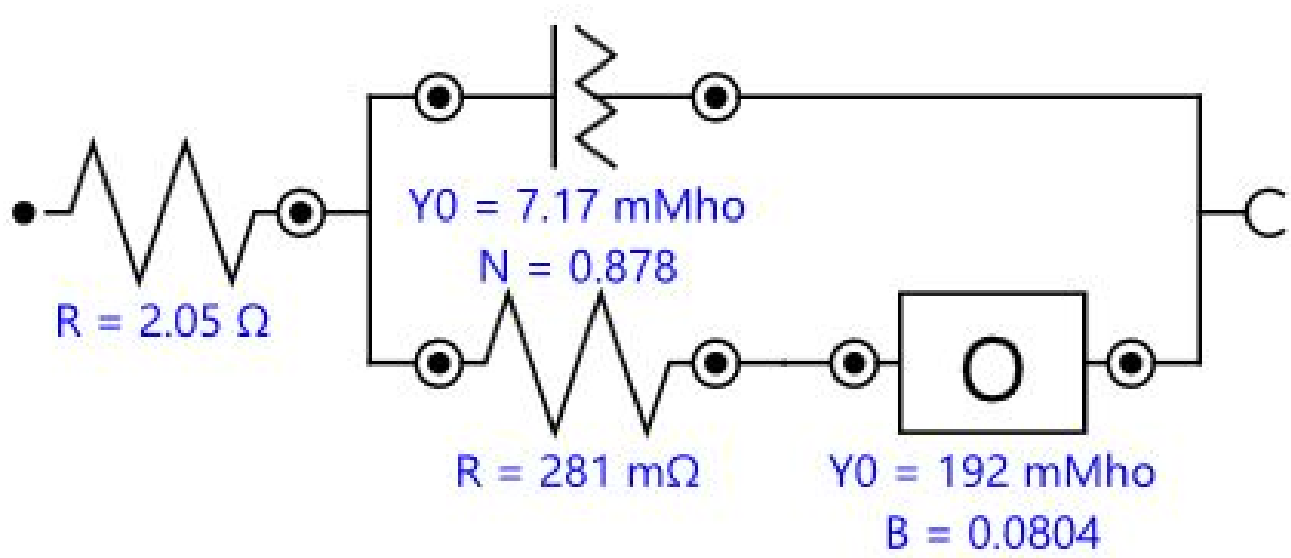

1.74	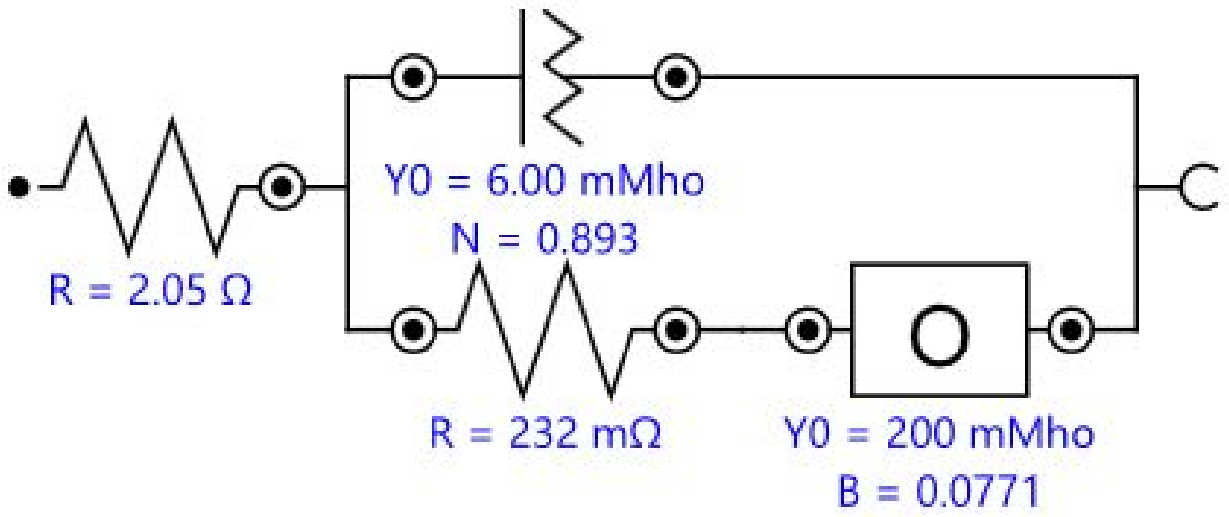Y0 = 6.00 mMho<br>N = 0.893<br>R = 2.05 Ω<br>R = 232 mΩ<br>O<br>Y0 = 200 mMho<br>B = 0.0771

Table S5: Equivalent circuit for NF at 1.70 V with varying lower frequency range and higher frequency kept fixed (10^5 Hz) with a 5 mV ac wave amplitude.

Frequency (Hz)	Equivalent circuit
100	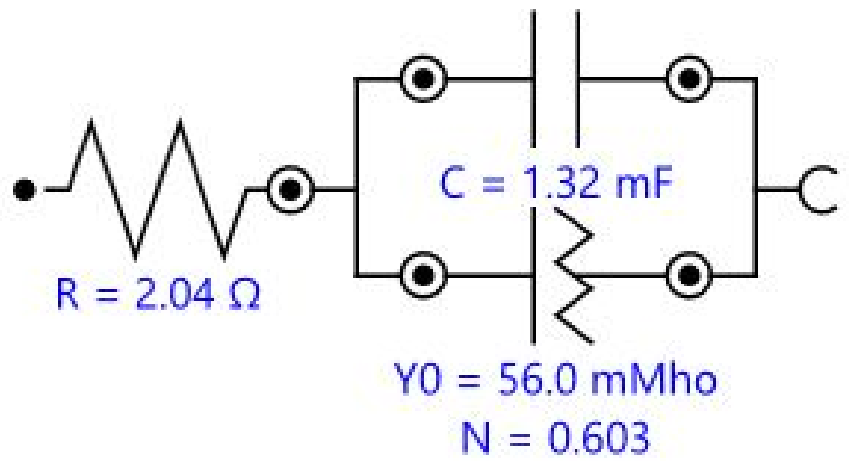
10	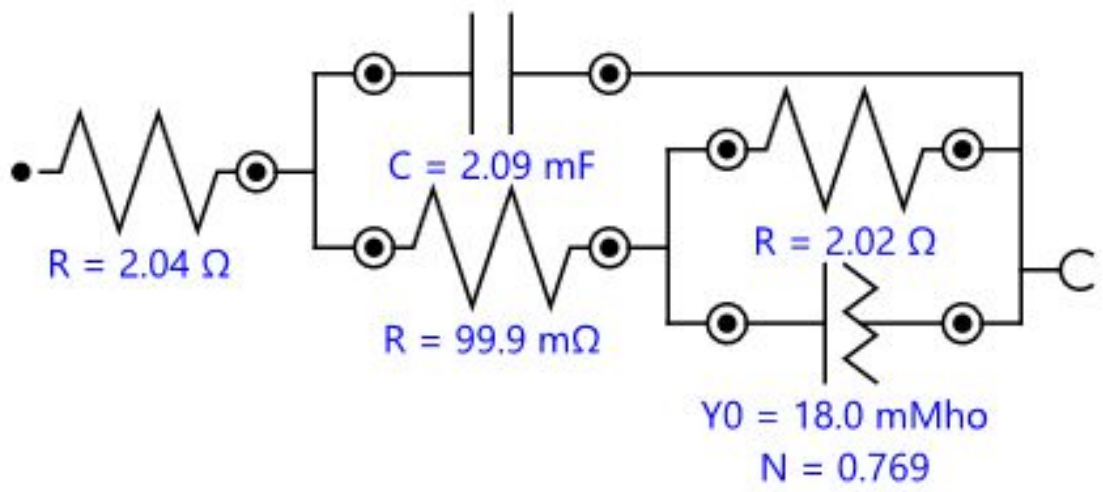
1	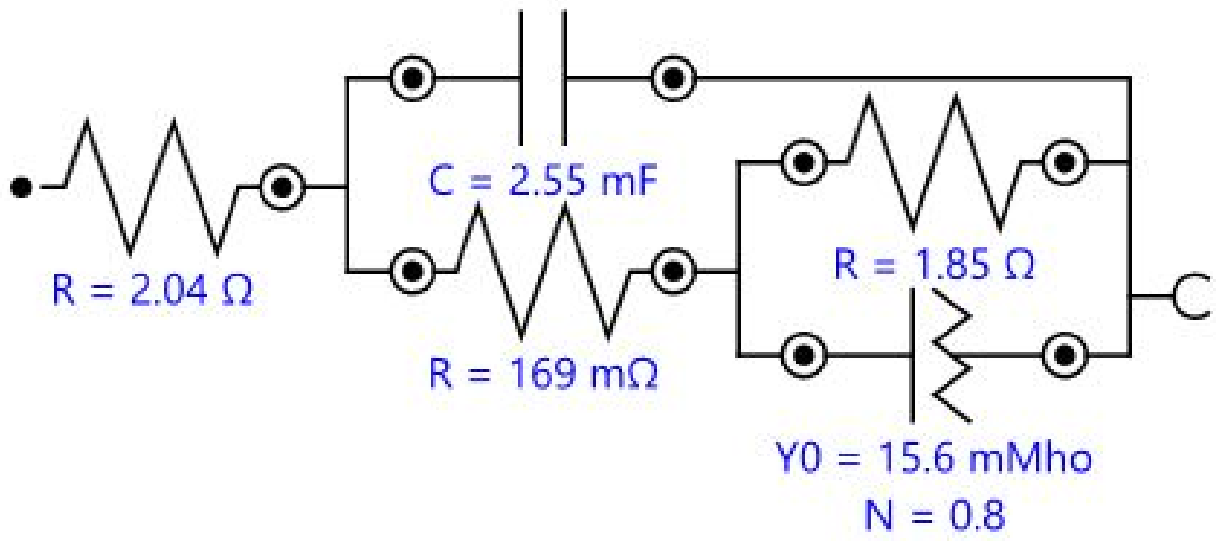
0.1	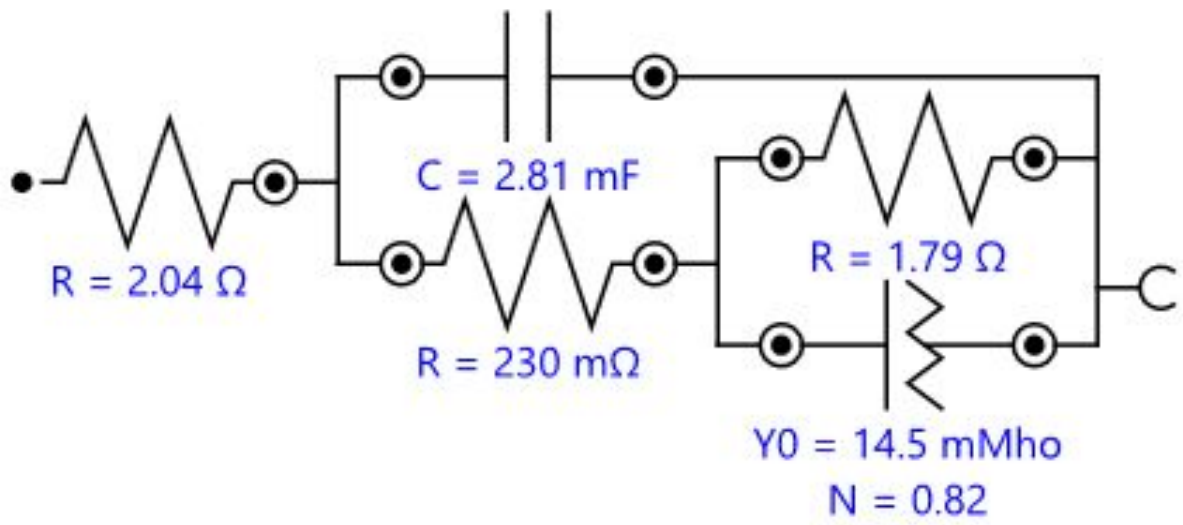
0.01	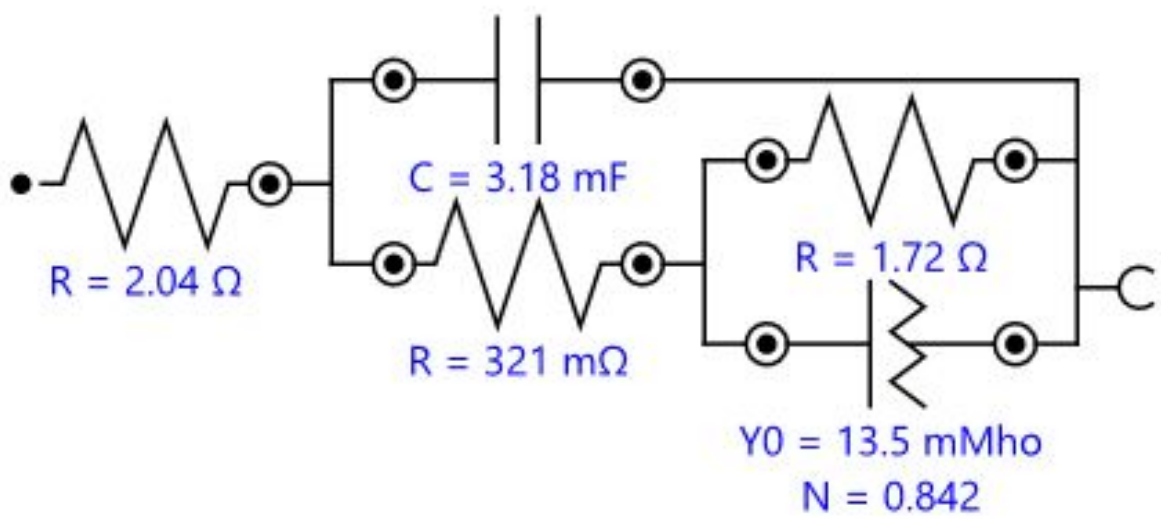

Table S6: Equivalent circuit for NF at 1.70 V with varying amplitude of ac wave at a frequency range of 10^5-1 Hz.

Amplitude (mV)	Equivalent circuit
1	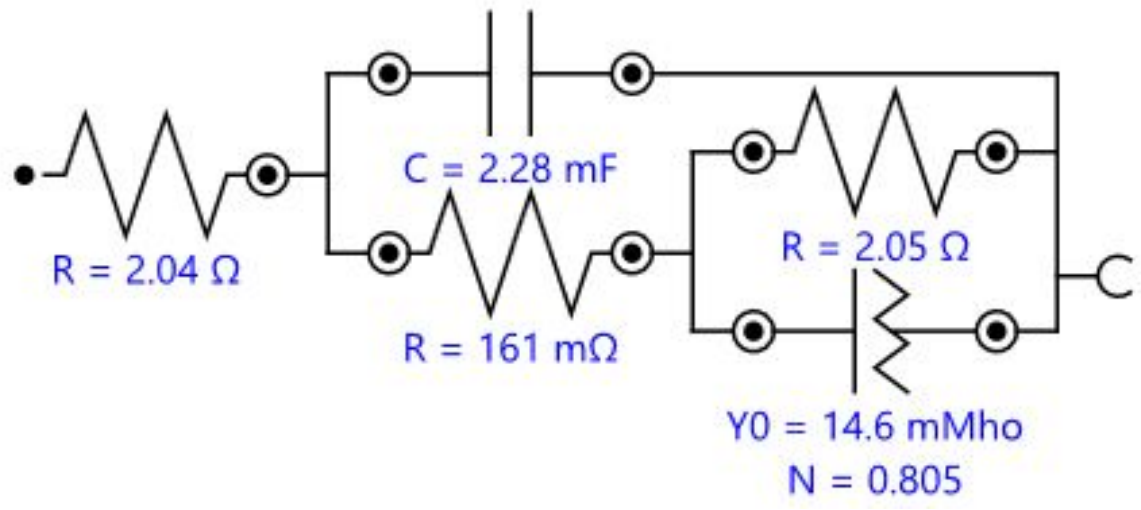
2	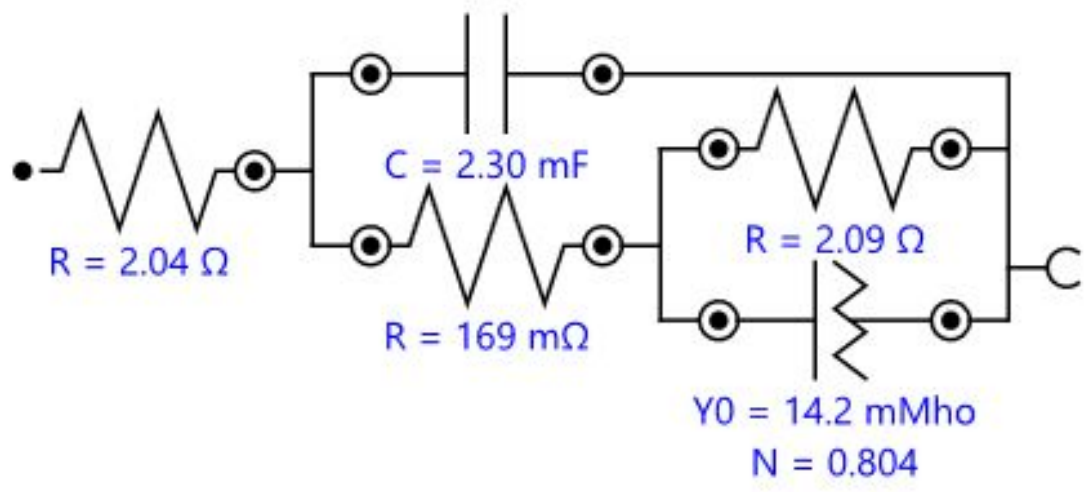
5	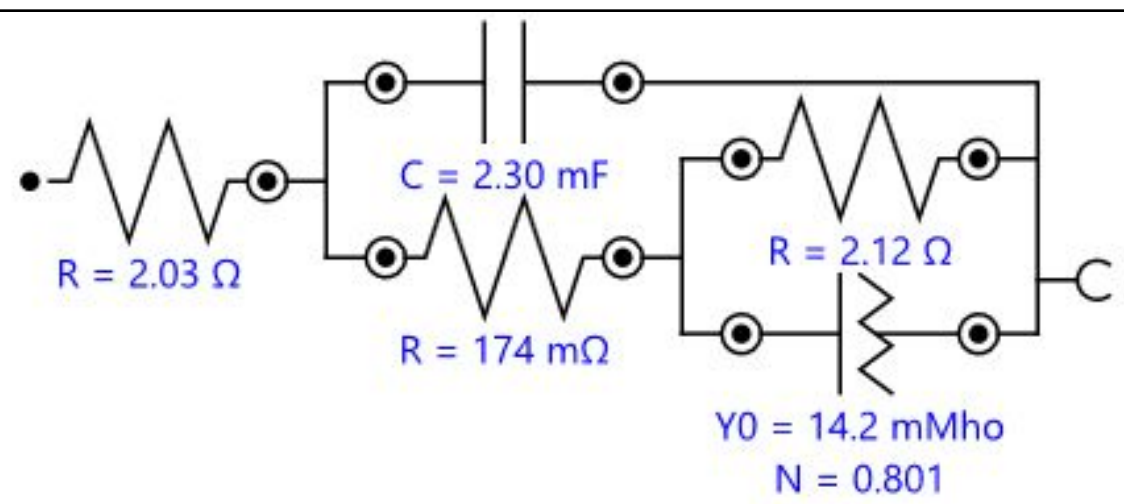
10	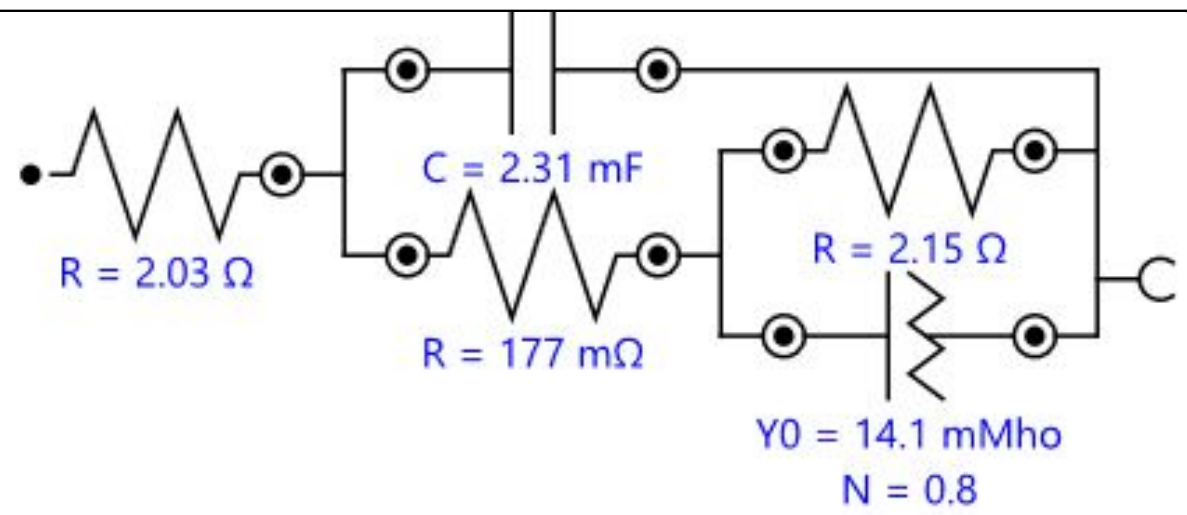

Table S7: Equivalent circuit for various MEA configurations in zero-gap alkaline water electrolyzer taken at 0 V in frequency range of 10^5-1 Hz and at 5 mV amplitude of ac wave.

Electrode configuration	Equivalent circuit
NF	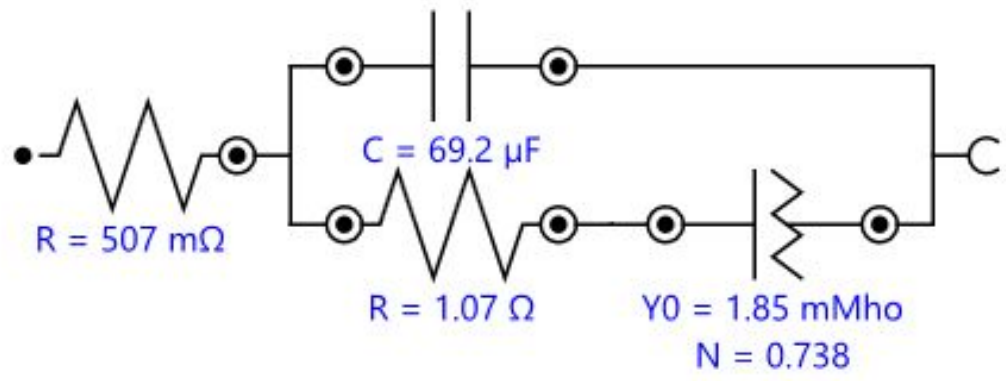
NFM	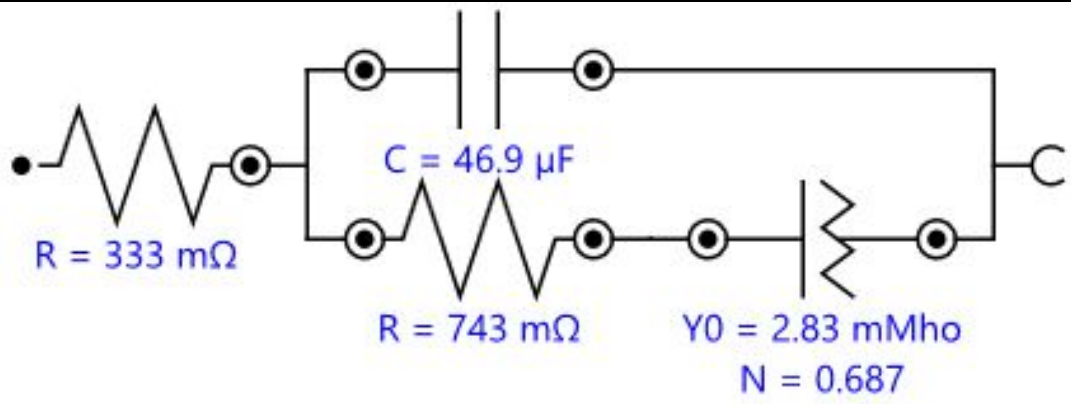
NFNF	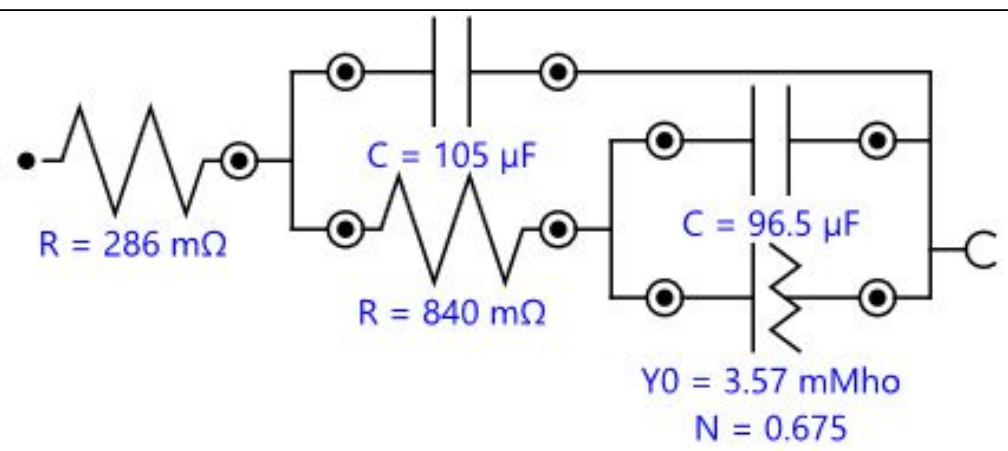
NFNFM	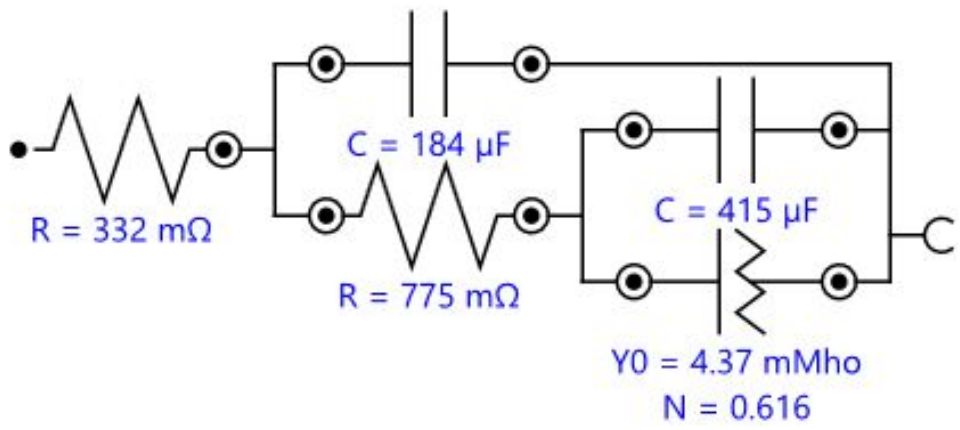

Table S8: Equivalent circuit for NFNFM (10 cm^2) configuration at variable parameters in zero-gap alkaline water electrolyzer.

Parameters	Equivalent circuit
Potential: 0 V<br>Frequency: 10^5-1 Hz<br>Amplitude: 5 mV	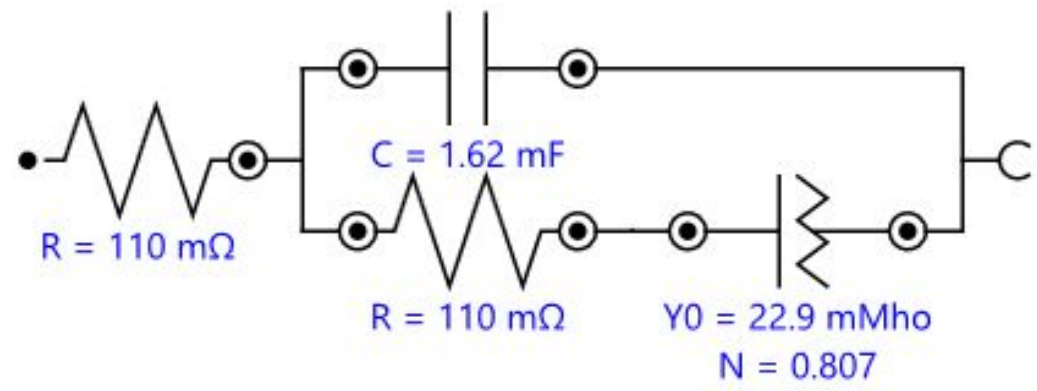
Current: 30 mA<br>Frequency: 10^5-0.1 Hz<br>Amplitude: 5 mA	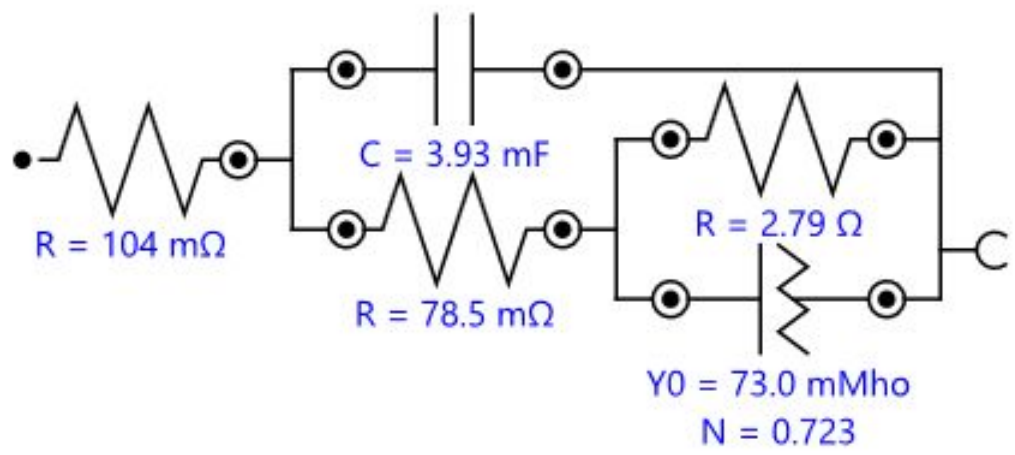
Current: 100 mA<br>Frequency: 10^5-0.1 Hz<br>Amplitude: 5 mA	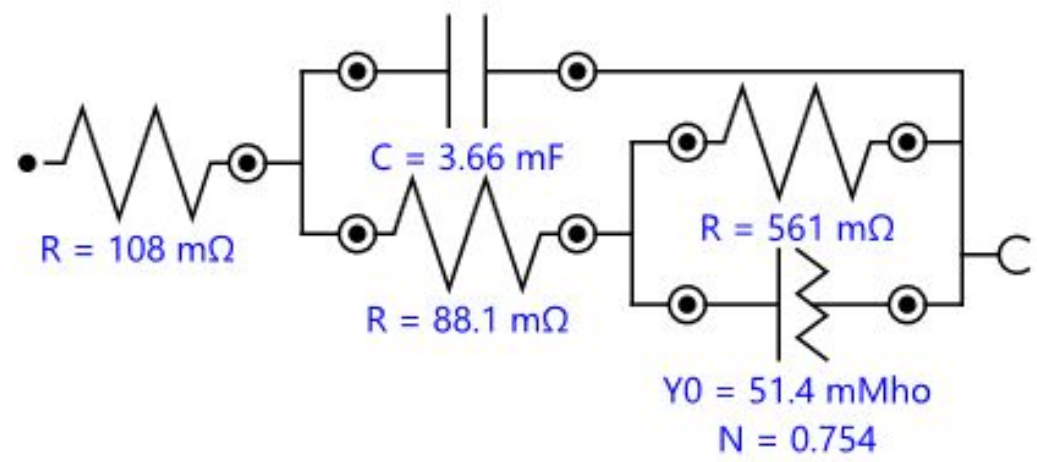
